# Supplementary material for: In-home and remote use of robotic body surrogates by people with profound motor deficits
Source: PLoS One. 2019 Mar 15;14(3):e0212904. doi: 10.1371/journal.pone.0212904 (PMC6420017; doi:10.1371/journal.pone.0212904)
Supplement: S1 Source Code — This includes all custom code used in the described system and related evaluations. (ZIP) [file pone.0212904.s009.zip › robotic_body_surrogates_source_code/hrl-haptic-manip-pkgs.zip!/hrl-haptic-manip-pkgs/hrl_fabric_based_tactile_sensor/arduino_sketchbook/ADS7828.pdf]

Burr-Brown Products from Texas Instruments

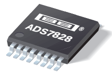

ADS7828

SBAS181C – NOVEMBER 2001 - REVISED MARCH 2005

12-Bit, 8-Channel Sampling ANALOG-TO-DIGITAL CONVERTER with I²C™ Interface

FEATURES

- 8-CHANNEL MULTIPLEXER
- 50kHz SAMPLING RATE
- NO MISSING CODES
- 2.7V TO 5V OPERATION
- INTERNAL 2.5V REFERENCE
- I²C INTERFACE SUPPORTS: Standard, Fast, and High-Speed Modes
- TSSOP-16 PACKAGE

APPLICATIONS

- VOLTAGE-SUPPLY MONITORING
- ISOLATED DATA ACQUISITION
- TRANSDUCER INTERFACES
- BATTERY-OPERATED SYSTEMS
- REMOTE DATA ACQUISITION

DESCRIPTION

The ADS7828 is a single-supply, low-power, 12-bit data acquisition device that features a serial I²C interface and an 8-channel multiplexer. The Analog-to-Digital (A/D) converter features a sample-and-hold amplifier and internal, asynchronous clock. The combination of an I²C serial, 2-wire interface and micropower consumption makes the ADS7828 ideal for applications requiring the A/D converter to be close to the input source in remote locations and for applications requiring isolation. The ADS7828 is available in a TSSOP-16 package.

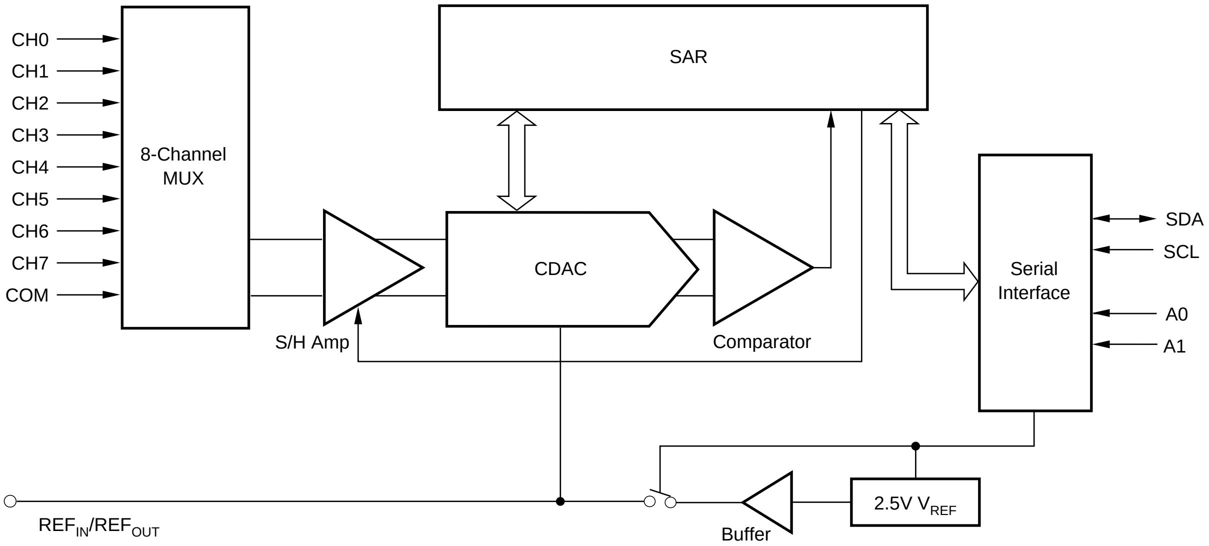

Please be aware that an important notice concerning availability, standard warranty, and use in critical applications of Texas Instruments semiconductor products and disclaimers thereto appears at the end of this data sheet.

I²C is a trademark of Koninklijke Philps Electronics N.V. All other trademarks are the property of their respective owners.

PRODUCTION DATA information is current as of publication date. Products conform to specifications per the terms of Texas Instruments standard warranty. Production processing does not necessarily include testing of all parameters.

Copyright © 2001-2005, Texas Instruments Incorporated

ABSOLUTE MAXIMUM RATINGS(1)

$+V_{DD}$ to GND	–0.3V to +6V
Digital Input Voltage to GND	–0.3V to $+V_{DD}$ + 0.3V
Operating Temperature Range	–40°C to +105°C
Storage Temperature Range	–65°C to +150°C
Junction Temperature (T_J max)	+150°C
TSSOP Package	
Power Dissipation	(T_J max – T_A)/θ_{JA}
θ_{JA} Thermal Impedance	240°C/W
Lead Temperature, Soldering	
Vapor Phase (60s)	+215°C
Infrared (15s)	+220°C

NOTE: (1) Stresses above those listed under *Absolute Maximum Ratings* may cause permanent damage to the device. Exposure to absolute maximum conditions for extended periods may affect device reliability.

ELECTROSTATIC DISCHARGE SENSITIVITY

This integrated circuit can be damaged by ESD. Texas Instruments recommends that all integrated circuits be handled with appropriate precautions. Failure to observe proper handling and installation procedures can cause damage.

ESD damage can range from subtle performance degradation to complete device failure. Precision integrated circuits may be more susceptible to damage because very small parametric changes could cause the device not to meet its published specifications.

PACKAGE/ORDERING INFORMATION(1)

PRODUCT	MAXIMUM INTEGRAL LINEARITY ERROR (LSB)	PACKAGE-LEAD	PACKAGE DESIGNATOR	SPECIFIED TEMPERATURE RANGE	ORDERING NUMBER	TRANSPORT MEDIA, QUANTITY
ADS7828E	±2	TSSOP-16	PW	–40°C to +85°C	ADS7828E/250	Tape and Reel, 250
"	"	"	"	"	ADS7828E/2K5	Tape and Reel, 2500
ADS7828EB	±1	TSSOP-16	PW	–40°C to +85°C	ADS7828EB/250	Tape and Reel, 250
"	"	"	"	"	ADS7828EB/2K5	Tape and Reel, 2500

NOTE: (1) For the most current package and ordering information, see the Package Option Addendum at the end of this data sheet, or see the TI web site at www.ti.com.

PIN CONFIGURATION

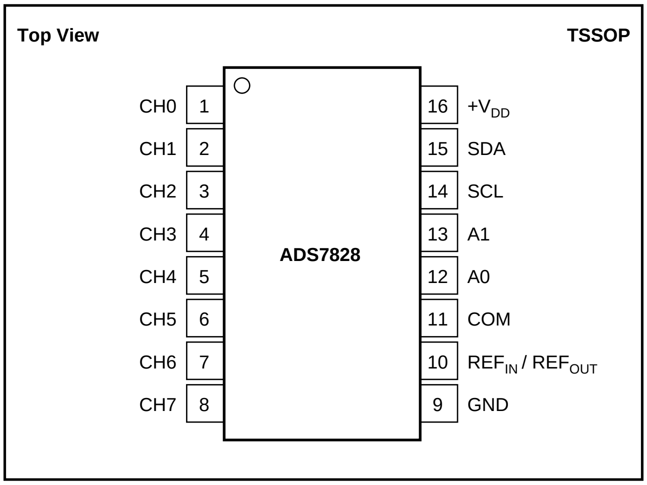

PIN DESCRIPTIONS

PIN	NAME	DESCRIPTION
1	CH0	Analog Input Channel 0
2	CH1	Analog Input Channel 1
3	CH2	Analog Input Channel 2
4	CH3	Analog Input Channel 3
5	CH4	Analog Input Channel 4
6	CH5	Analog Input Channel 5
7	CH6	Analog Input Channel 6
8	CH7	Analog Input Channel 7
9	GND	Analog Ground
10	REF_{IN} / REF_{OUT}	Internal +2.5V Reference, External Reference Input
11	COM	Common to Analog Input Channel
12	A0	Slave Address Bit 0
13	A1	Slave Address Bit 1
14	SCL	Serial Clock
15	SDA	Serial Data
16	$+V_{DD}$	Power Supply, 3.3V Nominal

ELECTRICAL CHARACTERISTICS: +2.7V

At T_A = –40°C to +85°C, $+V_{DD}$ = +2.7V, V_{REF} = +2.5V, SCL Clock Frequency = 3.4MHz (High-Speed Mode), unless otherwise noted.

PARAMETER	CONDITIONS	ADS7828E			ADS7828EB			UNITS
		MIN	TYP	MAX	MIN	TYP	MAX	
**ANALOG INPUT**								
Full-Scale Input Scan	Positive Input - Negative Input	0		V_{REF}	0		V_{REF}	V
Absolute Input Range	Positive Input	–0.2		$+V_{DD}$ + 0.2	–0.2		$+V_{DD}$ + 0.2	V
	Negative Input	–0.2		+0.2	–0.2		+0.2	V
Capacitance			25			25		pF
Leakage Current			±1			±1		µA
**SYSTEM PERFORMANCE**								
No Missing Codes		12			12			Bits
Integral Linearity Error			±1.0	±2		±0.5	±1	LSB[1]
Differential Linearity Error			±1.0			±0.5	–1, +2	LSB
Offset Error			±1.0	±3		±0.75	±2	LSB
Offset Error Match			±0.2	±1		±0.2	±1	LSB
Gain Error			±1.0	±4		±0.75	±3	LSB
Gain Error Match			±0.2	±1		±0.2	±1	LSB
Noise			33			33		µVRMS
Power-Supply Rejection			82			82		dB
**SAMPLING DYNAMICS**								
Throughput Frequency	High Speed Mode: SCL = 3.4MHz			50			50	kHz
	Fast Mode: SCL = 400kHz			8			8	
	Standard Mode, SCL = 100kHz			2			2	kHz
Conversion Time			6			6		µs
**AC ACCURACY**								
Total Harmonic Distortion	V_{IN} = 2.5V_{PP} at 10kHz		–82			–82		dB[2]
Signal-to-Ratio	V_{IN} = 2.5V_{PP} at 10kHz		72			72		dB
Signal-to-(Noise+Distortion) Ratio	V_{IN} = 2.5V_{PP} at 10kHz		71			71		dB
Spurious-Free Dynamic Range	V_{IN} = 2.5V_{PP} at 10kHz		86			86		dB
Isolation Channel-to-Channel			120			120		dB
**VOLTAGE REFERENCE OUTPUT**								
Range		2.475	2.5	2.525	2.475	2.5	2.525	V
Internal Reference Drift			15			15		ppm/°C
Output Impedance	Internal Reference ON		110			110		Ω
	Internal Reference OFF		1			1		GΩ
Quiescent Current	Int. Ref. ON, SCL and SDA pulled HIGH		850			850		µA
**VOLTAGE REFERENCE INPUT**								
Range		0.05		V_{DD}	0.05		V_{DD}	V
Resistance			1			1		GΩ
Current Drain	High Speed Mode: SCL= 3.4MHz		20			20		µA
**DIGITAL INPUT/OUTPUT**								
Logic Family			CMOS			CMOS		
Logic Levels: V_{IH}		$+V_{DD}$ • 0.7		$+V_{DD}$ + 0.5	$+V_{DD}$ • 0.7		$+V_{DD}$ + 0.5	V
V_{IL}		–0.3		$+V_{DD}$ • 0.3	–0.3		$+V_{DD}$ • 0.3	V
V_{OL}	Min. 3mA Sink Current			0.4			0.4	V
Input Leakage: I_{IH}	V_{IH} = $+V_{DD}$ +0.5			10			10	µA
I_{IL}	V_{IL} = -0.3	–10			–10			µA
Data Format			Straight Binary			Straight Binary		
**ADS7828 HARDWARE ADDRESS**			10010			10010		Binary
**POWER-SUPPLY REQUIREMENTS**								
Power-Supply Voltage, $+V_{DD}$	Specified Performance	2.7		3.6	2.7		3.6	V
Quiescent Current	High Speed Mode: SCL = 3.4MHz		225	320		225	320	µA
	Fast Mode: SCL = 400kHz		100			100		µA
	Standard Mode, SCL = 100kHz		60			60		µA
Power Dissipation	High Speed Mode: SCL = 3.4MHz		675	1000		675	1000	µW
	Fast Mode: SCL = 400kHz		300			300		µW
	Standard Mode, SCL = 100kHz		180			180		µW
Power-Down Mode	High Speed Mode: SCL = 3.4MHz		70			70		µA
w/Wrong Address Selected	Fast Mode: SCL = 400kHz		25			25		µA
	Standard Mode, SCL = 100kHz		6			6		µA
Full Power-Down	SCL Pulled HIGH, SDA Pulled HIGH		400	3000		400	3000	nA
**TEMPERATURE RANGE**								
Specified Performance		–40		85	–40		85	°C

NOTES: (1) LSB means Least Significant Bit. With V_{REF} equal to 2.5V, 1LSB is 610µV.
(2) THD measured out to the 9th-harmonic.

ELECTRICAL CHARACTERISTICS: +5V

At T_A = –40°C to +85°C, $+V_{DD}$ = +5.0V, V_{REF} = External +5.0V, SCL Clock Frequency = 3.4MHz (High-Speed Mode), unless otherwise noted.

PARAMETER	CONDITIONS	ADS7828E			ADS7828EB			UNITS
		MIN	TYP	MAX	MIN	TYP	MAX	
**ANALOG INPUT**								
Full-Scale Input Scan	Positive Input - Negative Input	0		V_{REF}	0		V_{REF}	V
Absolute Input Range	Positive Input	–0.2		$+V_{DD}$ + 0.2	–0.2		$+V_{DD}$ + 0.2	V
	Negative Input	–0.2		+0.2	–0.2		+0.2	V
Capacitance			25			25		pF
Leakage Current			±1			±1		µA
**SYSTEM PERFORMANCE**								
No Missing Codes		12			12			Bits
Integral Linearity Error			±1.0	±2		±0.5	±1	LSB(1)
Differential Linearity Error			±1.0			±0.5	–1, +2	LSB
Offset Error			±1.0	±3		±0.75	±2	LSB
Offset Error Match				±1			±1	LSB
Gain Error			±1.0	±3		±0.75	±2	LSB
Gain Error Match				±1			±1	LSB
Noise			33			33		µVRMS
Power-Supply Rejection			82			82		dB
**SAMPLING DYNAMICS**								
Throughput Frequency	High Speed Mode: SCL = 3.4MHz			50			50	kHz
	Fast Mode: SCL = 400kHz			8			8	kHz
	Standard Mode, SCL = 100kHz			2			2	kHz
Conversion Time			6			6		µs
**AC ACCURACY**								
Total Harmonic Distortion	V_{IN} = 2.5V_{PP} at 10kHz		–82			–82		dB(2)
Signal-to-Ratio	V_{IN} = 2.5V_{PP} at 10kHz		72			72		dB
Signal-to-(Noise+Distortion) Ratio	V_{IN} = 2.5V_{PP} at 10kHz		71			71		dB
Spurious-Free Dynamic Range	V_{IN} = 2.5V_{PP} at 10kHz		86			86		dB
Isolation Channel-to-Channel			120			120		dB
**VOLTAGE REFERENCE OUTPUT**								
Range		2.475	2.5	2.525	2.475	2.5	2.525	V
Internal Reference Drift			15			15		ppm/°C
Output Impedance	Internal Reference ON		110			110		Ω
	Internal Reference OFF		1			1		GΩ
Quiescent Current	Int. Ref. ON, SCL and SDA pulled HIGH		1300			1300		µA
**VOLTAGE REFERENCE INPUT**								
Range		0.05		V_{DD}	0.05		V_{DD}	V
Resistance			1			1		GΩ
Current Drain	High Speed Mode: SCL = 3.4MHz		20			20		µA
**DIGITAL INPUT/OUTPUT**								
Logic Family		CMOS			CMOS			
Logic Levels: V_{IH}		$+V_{DD}$ • 0.7		$+V_{DD}$ + 0.5	$+V_{DD}$ • 0.7		$+V_{DD}$ + 0.5	V
V_{IL}		–0.3		$+V_{DD}$ • 0.3	–0.3		$+V_{DD}$ • 0.3	V
V_{OL}	Min. 3mA Sink Current			0.4			0.4	V
Input Leakage: I_{IH}	V_{IH} = $+V_{DD}$ +0.5			10			10	µA
I_{IL}	V_{IL} = -0.3	–10			–10			µA
Data Format			Straight Binary			Straight Binary		
**ADS7828 HARDWARE ADDRESS**			10010			10010		Binary
**POWER-SUPPLY REQUIREMENTS**								
Power-Supply Voltage, $+V_{DD}$	Specified Performance	4.75	5	5.25	4.75	5	5.25	V
Quiescent Current	High Speed Mode: SCL = 3.4MHz		750	1000		750	1000	µA
	Fast Mode: SCL = 400kHz		300			300		µA
	Standard Mode, SCL = 100kHz		150			150		µA
Power Dissipation	High Speed Mode: SCL = 3.4MHz		3.75	5		3.75	5	mW
	Fast Mode: SCL = 400kHz		1.5			1.5		mW
	Standard Mode, SCL = 100kHz		0.75			0.75		mW
Power-Down Mode	High Speed Mode: SCL = 3.4MHz		400			400		µA
w/Wrong Address Selected	Fast Mode: SCL = 400kHz		150			150		µA
	Standard Mode, SCL = 100kHz		35			35		µA
Full Power-Down	SCL Pulled HIGH, SDA Pulled HIGH		400	3000		400	3000	nA
**TEMPERATURE RANGE**								
Specified Performance		–40		+85	–40		+85	°C

NOTES: (1) LSB means Least Significant Bit. With V_{REF} equal to 5.0V, 1LSB is 1.22mV.
(2) THD measured out to the 9th-harmonic.

TIMING DIAGRAM

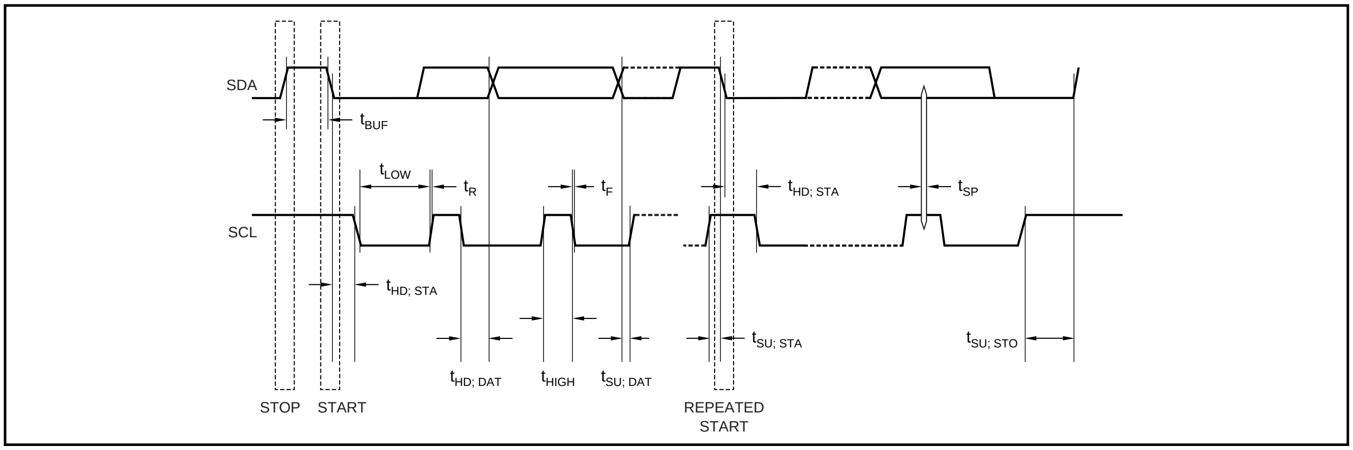

TIMING CHARACTERISTICS(1)

At T_A = –40°C to +85°C, $+V_{DD}$ = +2.7V, unless otherwise noted.

PARAMETER	SYMBOL	CONDITIONS	MIN	MAX	UNITS
SCL Clock Frequency	f_{SCL}	Standard Mode<br>Fast Mode<br>High-Speed Mode, C_B = 100pF max<br>High-Speed Mode, C_B = 400pF max		100<br>400<br>3.4<br>1.7	kHz<br>kHz<br>MHz<br>MHz
Bus Free Time Between a STOP and START Condition	t_{BUF}	Standard Mode<br>Fast Mode	4.7<br>1.3		µs<br>µs
Hold Time (Repeated) START Condition	$t_{HD;STA}$	Standard Mode<br>Fast Mode<br>High-Speed Mode	4.0<br>600<br>160		µs<br>ns<br>ns
LOW Period of the SCL Clock	t_{LOW}	Standard Mode<br>Fast Mode<br>High-Speed Mode, C_B = 100pF max(2)<br>High-Speed Mode, C_B = 400pF max(2)	4.7<br>1.3<br>160<br>320		µs<br>µs<br>ns<br>ns
HIGH Period of the SCL Clock	t_{HIGH}	Standard Mode<br>Fast Mode<br>High-Speed Mode, C_B = 100pF max(2)<br>High-Speed Mode, C_B = 400pF max(2)	4.0<br>600<br>60<br>120		µs<br>ns<br>ns<br>ns
Setup Time for a Repeated START Condition	$t_{SU;STA}$	Standard Mode<br>Fast Mode<br>High-Speed Mode	4.7<br>600<br>160		µs<br>ns<br>ns
Data Setup Time	$t_{SU;DAT}$	Standard Mode<br>Fast Mode<br>High-Speed Mode	250<br>100<br>10		ns<br>ns<br>ns
Data Hold Time	$t_{HD;DAT}$	Standard Mode<br>Fast Mode<br>High-Speed Mode, C_B = 100pF max(2)<br>High-Speed Mode, C_B = 400pF max(2)	0<br>0<br>0(3)<br>0(3)	3.45<br>0.9<br>70<br>150	µs<br>µs<br>ns<br>ns
Rise Time of SCL Signal	t_{RCL}	Standard Mode<br>Fast Mode<br>High-Speed Mode, C_B = 100pF max(2)<br>High-Speed Mode, C_B = 400pF max(2)	 20 + $0.1C_B$<br>10<br>20	1000<br>300<br>40<br>80	ns<br>ns<br>ns<br>ns
Rise Time of SCL Signal After a Repeated START Condition and After an Acknowledge Bit	t_{RCL1}	Standard Mode<br>Fast Mode<br>High-Speed Mode, C_B = 100pF max(2)<br>High-Speed Mode, C_B = 400pF max(2)	 20 + $0.1C_B$<br>10<br>20	1000<br>300<br>80<br>160	ns<br>ns<br>ns<br>ns
Fall Time of SCL Signal	t_{FCL}	Standard Mode<br>Fast Mode<br>High-Speed Mode, C_B = 100pF max(2)<br>High-Speed Mode, C_B = 400pF max(2)	 20 + $0.1C_B$<br>10<br>20	300<br>300<br>40<br>80	ns<br>ns<br>ns<br>ns

NOTES: (1) All values referred to V_{IHMIN} and V_{ILMAX} levels.
(2) For bus line loads C_B between 100pF and 400pF the timing parameters must be linearly interpolated.
(3) A device must internally provide a data hold time to bridge the undefined part between V_{IH} and V_{IL} of the falling edge of the SCLH signal. An input circuit with a threshold as low as possible for the falling edge of the SCLH signal minimizes this hold time.

TIMING CHARACTERISTICS[1] (Cont.)

At T_A = –40°C to +85°C, $+V_{DD}$ = +2.7V, unless otherwise noted.

PARAMETER	SYMBOL	CONDITIONS	MIN	MAX	UNITS
Rise Time of SDA Signal	t_{RDA}	Standard Mode<br>Fast Mode<br>High-Speed Mode, C_B = 100pF max[2]<br>High-Speed Mode, C_B = 400pF max[2]	 20 + $0.1C_B$<br>10<br>20	1000<br>300<br>80<br>160	ns<br>ns<br>ns<br>ns
Fall Time of SDA Signal	t_{FDA}	Standard Mode<br>Fast Mode<br>High-Speed Mode, C_B = 100pF max[2]<br>High-Speed Mode, C_B = 400pF max[2]	 20 + $0.1C_B$<br>10<br>20	300<br>300<br>80<br>160	ns<br>ns<br>ns<br>ns
Setup Time for STOP Condition	$t_{SU; STO}$	Standard Mode<br>Fast Mode<br>High-Speed Mode	4.0<br>600<br>160		µs<br>ns<br>ns
Capacitive Load for SDA and SCL Line	C_B			400	pF
Pulse Width of Spike Suppressed	t_{SP}	Fast Mode<br>High-Speed Mode		50<br>10	ns<br>ns
Noise Margin at the HIGH Level for Each Connected Device (Including Hysteresis)	V_{NH}	Standard Mode<br>Fast Mode<br>High-Speed Mode	$0.2V_{DD}$		V
Noise Margin at the LOW Level for Each Connected Device (Including Hysteresis)	V_{NL}	Standard Mode<br>Fast Mode<br>High-Speed Mode	$0.1V_{DD}$		V

NOTES: (1) All values referred to V_{IHMIN} and V_{ILMAX} levels.
(2) For bus line loads C_B between 100pF and 400pF the timing parameters must be linearly interpolated.
(3) A device must internally provide a data hold time to bridge the undefined part between V_{IH} and V_{IL} of the falling edge of the SCLH signal. An input circuit with a threshold as low as possible for the falling edge of the SCLH signal minimizes this hold time.

TYPICAL CHARACTERISTICS

T_A = +25°C, V_{DD} = +2.7V, V_{REF} = External +2.5V, f_{SAMPLE} = 50kHz, unless otherwise noted.

FREQUENCY SPECTRUM
(4096 Point FFT: f_{IN} = 1kHz, 0dB)

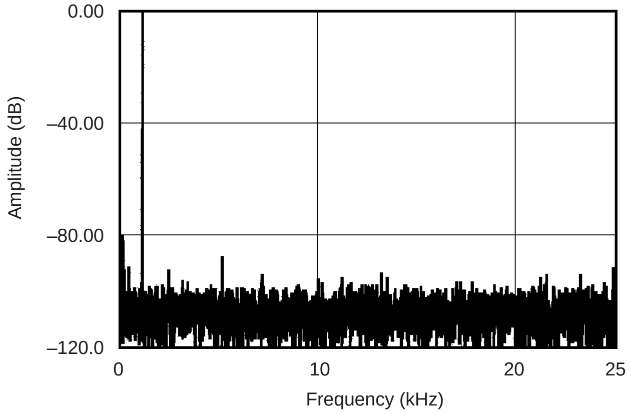

INTEGRAL LINEARITY ERROR vs CODE
(2.5V Internal Reference)

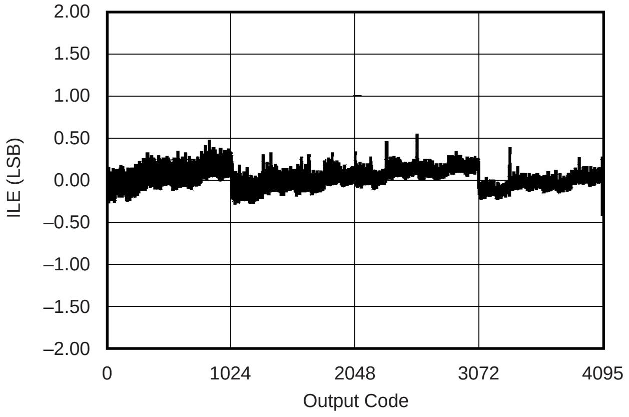

DIFFERENTIAL LINEARITY ERROR vs CODE
(2.5V Internal Reference)

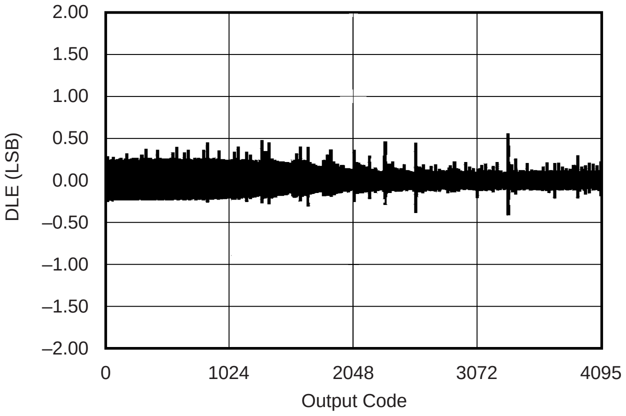

INTEGRAL LINEARITY ERROR vs CODE
(2.5V External Reference)

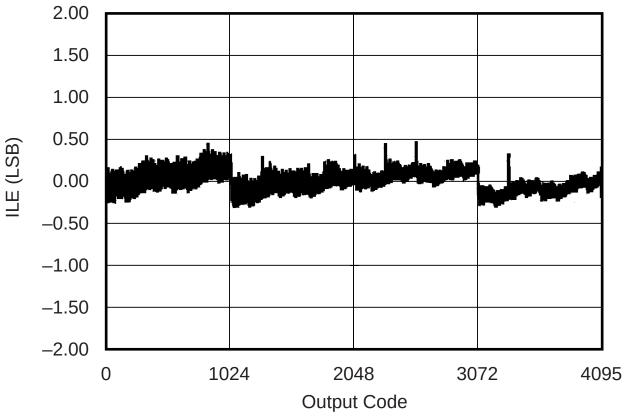

DIFFERENTIAL LINEARITY ERROR vs CODE
(2.5V External Reference)

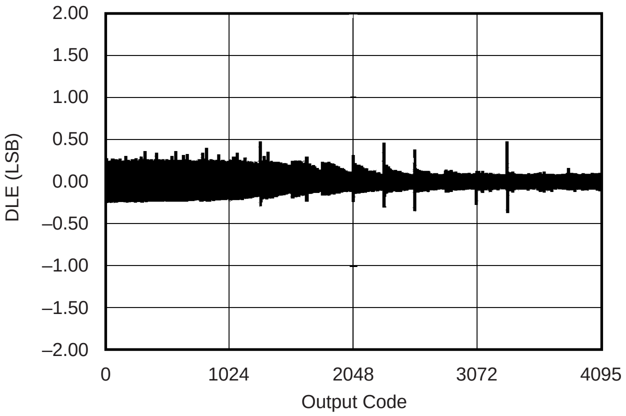

CHANGE IN OFFSET vs TEMPERATURE

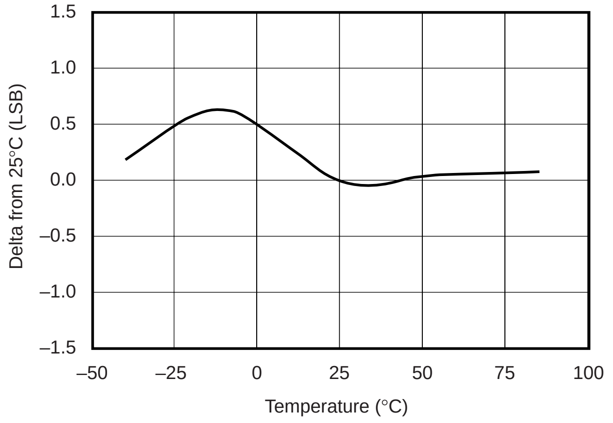

T_A = +25°C, V_{DD} = +2.7V, V_{REF} = External +2.5V, f_{SAMPLE} = 50kHz, unless otherwise noted.

CHANGE IN GAIN vs TEMPERATURE

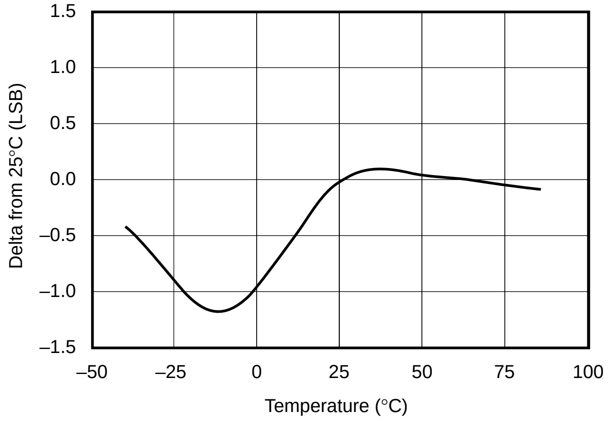

INTERNAL REFERENCE vs TEMPERATURE

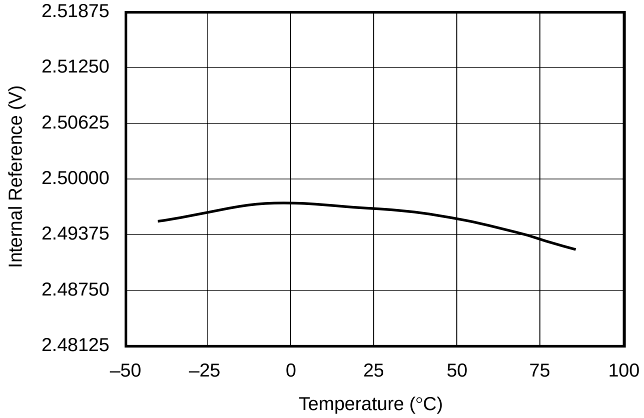

POWER-DOWN SUPPLY CURRENT vs TEMPERATURE

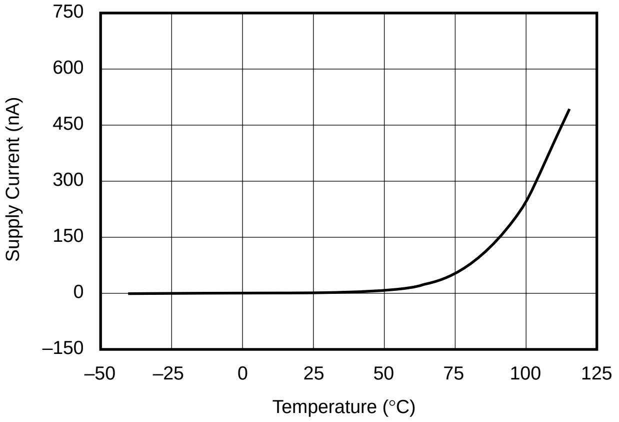

SUPPLY CURRENT vs TEMPERATURE

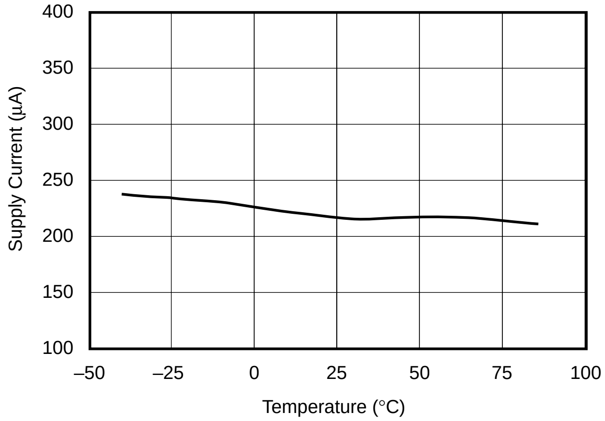

SUPPLY CURRENT vs I^2C BUS RATE

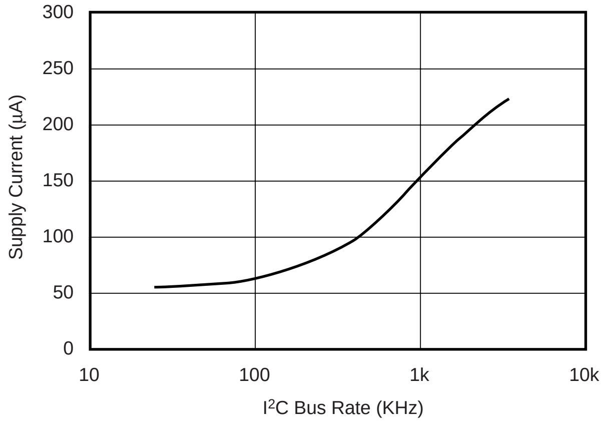

INTERNAL V_{REF} vs TURN-ON TIME

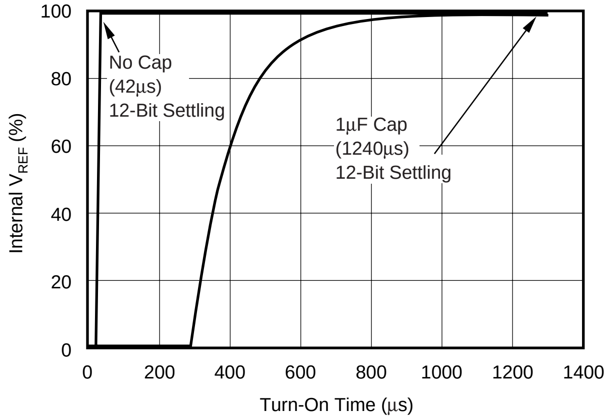

THEORY OF OPERATION

The ADS7828 is a classic Successive Approximation Register (SAR) A/D converter. The architecture is based on capacitive redistribution which inherently includes a sample-and-hold function. The converter is fabricated on a 0.6µ CMOS process.

The ADS7828 core is controlled by an internally generated free-running clock. When the ADS7828 is not performing conversions or being addressed, it keeps the A/D converter core powered off, and the internal clock does not operate.

The simplified diagram of input and output for the ADS7828 is shown in Figure 1.

ANALOG INPUT

When the converter enters the hold mode, the voltage on the selected CHx pin is captured on the internal capacitor array. The input current on the analog inputs depends on the conversion rate of the device. During the sample period, the source must charge the internal sampling capacitor (typically 25pF). After the capacitor has been fully charged, there is no further input current. The amount of charge transfer from the analog source to the converter is a function of conversion rate.

REFERENCE

The ADS7828 can operate with an internal 2.5V reference or an external reference. If a +5V supply is used, an external +5V reference is required in order to provide full dynamic range for a 0V to $+V_{DD}$ analog input. This external reference can be as low as 50mV. When using a +2.7V supply, the internal +2.5V reference will provide full dynamic range for a 0V to $+V_{DD}$ analog input.

As the reference voltage is reduced, the analog voltage weight of each digital output code is reduced. This is often referred to as the LSB (least significant bit) size and is equal to the reference voltage divided by 4096. This means that any offset or gain error inherent in the A/D converter will appear to increase, in terms of LSB size, as the reference voltage is reduced.

The noise inherent in the converter will also appear to increase with lower LSB size. With a 2.5V reference, the internal noise of the converter typically contributes only 0.32LSB peak-to-peak of potential error to the output code. When the external reference is 50mV, the potential error contribution from the internal noise will be 50 times larger—16LSBs. The errors due to the internal noise are Gaussian in nature and can be reduced by averaging consecutive conversion results.

DIGITAL INTERFACE

The ADS7828 supports the I^2C serial bus and data transmission protocol, in all three defined modes: standard, fast, and high-speed. A device that sends data onto the bus is defined as a transmitter, and a device receiving data as a receiver. The device that controls the message is called a “master.” The devices that are controlled by the master are “slaves.” The bus must be controlled by a master device that generates the serial clock (SCL), controls the bus access, and generates the START and STOP conditions. The ADS7828 operates as a slave on the I^2C bus. Connections to the bus are made via the open-drain I/O lines SDA and SCL.

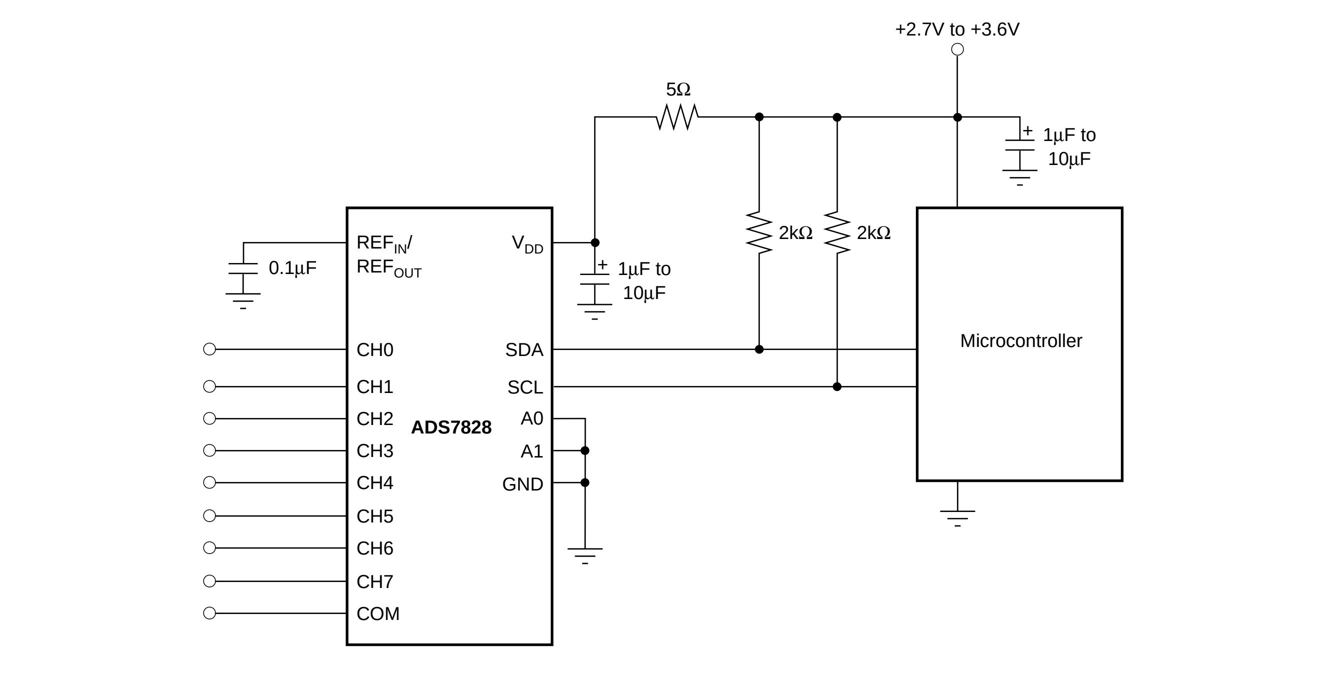

FIGURE 1. Simplified I/O of the ADS7828.

The following bus protocol has been defined (as shown in Figure 2):

- Data transfer may be initiated only when the bus is not busy.
- During data transfer, the data line must remain stable whenever the clock line is HIGH. Changes in the data line while the clock line is HIGH will be interpreted as control signals.

Accordingly, the following bus conditions have been defined:

Bus Not Busy: Both data and clock lines remain HIGH.

Start Data Transfer: A change in the state of the data line, from HIGH to LOW, while the clock is HIGH, defines a START condition.

Stop Data Transfer: A change in the state of the data line, from LOW to HIGH, while the clock line is HIGH, defines the STOP condition.

Data Valid: The state of the data line represents valid data, when, after a START condition, the data line is stable for the duration of the HIGH period of the clock signal. There is one clock pulse per bit of data.

Each data transfer is initiated with a START condition and terminated with a STOP condition. The number of data bytes transferred between START and STOP conditions is not limited and is determined by the master device. The information is transferred byte-wise and each receiver acknowledges with a ninth-bit.

Within the I^2C bus specifications a standard mode (100kHz clock rate), a fast mode (400kHz clock rate), and a high-speed mode (3.4MHz clock rate) are defined. The ADS7828 works in all three modes.

Acknowledge: Each receiving device, when addressed, is obliged to generate an acknowledge after the reception of each byte. The master device must generate an extra clock pulse that is associated with this acknowledge bit.

A device that acknowledges must pull down the SDA line during the acknowledge clock pulse in such a way that the SDA line is stable LOW during the HIGH period of the acknowledge clock pulse. Of course, setup and hold times must be taken into account. A master must signal an end of data to the slave by not generating an acknowledge bit on the last byte that has been clocked out of the slave. In this case, the slave must leave the data line HIGH to enable the master to generate the STOP condition.

Figure 2 details how data transfer is accomplished on the I^2C bus. Depending upon the state of the R/$\overline{W}$ bit, two types of data transfer are possible:

1. **Data transfer from a master transmitter to a slave receiver.** The first byte transmitted by the master is the slave address. Next follows a number of data bytes. The slave returns an acknowledge bit after the slave address and each received byte.
2. **Data transfer from a slave transmitter to a master receiver.** The first byte, the slave address, is transmitted by the master. The slave then returns an acknowledge bit. Next, a number of data bytes are transmitted by the slave to the master. The master returns an acknowledge bit after all received bytes other than the last byte. At the end of the last received byte, a not-acknowledge is returned.

The master device generates all of the serial clock pulses and the START and STOP conditions. A transfer is ended with a STOP condition or a repeated START condition. Since a repeated START condition is also the beginning of the next serial transfer, the bus will not be released.

The ADS7828 may operate in the following two modes:

- **Slave Receiver Mode:** Serial data and clock are received through SDA and SCL. After each byte is received, an acknowledge bit is transmitted. START and STOP conditions are recognized as the beginning and end of a serial transfer. Address recognition is performed by hardware after reception of the slave address and direction bit.
- **Slave Transmitter Mode:** The first byte (the slave address) is received and handled as in the slave receiver mode. However, in this mode the direction bit will indicate that the transfer direction is reversed. Serial data is transmitted on SDA by the ADS7828 while the serial clock is input on SCL. START and STOP conditions are recognized as the beginning and end of a serial transfer.

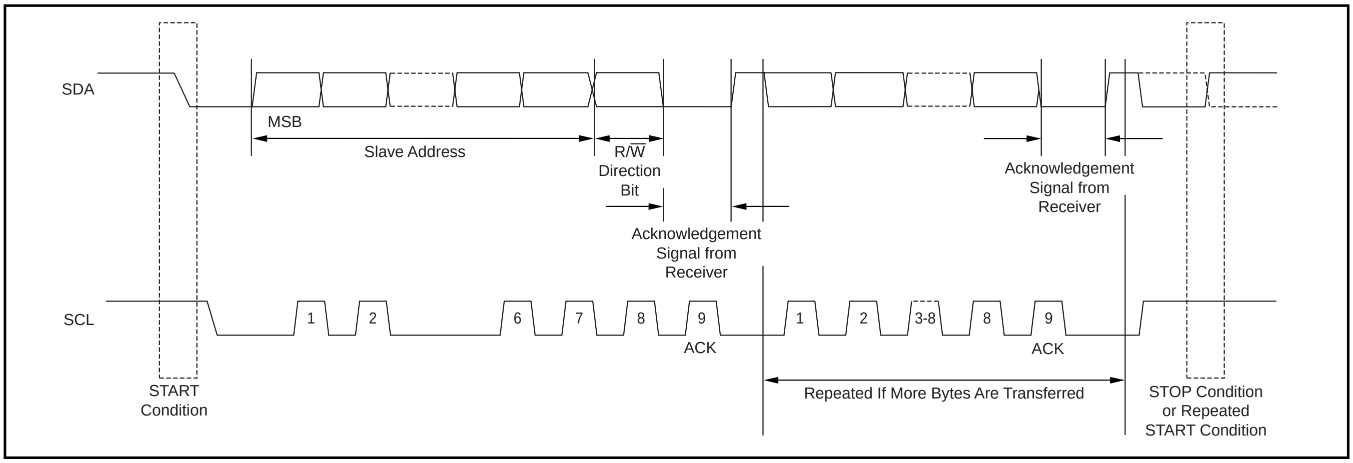

FIGURE 2. Basic Operation of the ADS7828.

ADDRESS BYTE

MSB	6	5	4	3	2	1	LSB
1	0	0	1	0	A1	A0	R/$\overline{W}$

The address byte is the first byte received following the START condition from the master device. The first five bits (MSBs) of the slave address are factory pre-set to 10010. The next two bits of the address byte are the device select bits, A1 and A0. Input pins (A1-A0) on the ADS7828 determine these two bits of the device address for a particular ADS7828. A maximum of four devices with the same pre-set code can therefore be connected on the same bus at one time.

The A1-A0 Address Inputs can be connected to V_{DD} or digital ground. The device address is set by the state of these pins upon power-up of the ADS7828.

The last bit of the address byte (R/$\overline{W}$) defines the operation to be performed. When set to a '1' a read operation is selected; when set to a '0' a write operation is selected. Following the START condition the ADS7828 monitors the SDA bus, checking the device type identifier being transmitted. Upon receiving the 10010 code, the appropriate device select bits, and the R/$\overline{W}$ bit, the slave device outputs an acknowledge signal on the SDA line.

COMMAND BYTE

MSB	6	5	4	3	2	1	LSB
SD	C2	C1	C0	PD1	PD0	X	X

The ADS7828 operating mode is determined by a command byte which is illustrated above.

SD: Single-Ended/Differential Inputs
  0: Differential Inputs
  1: Single-Ended Inputs

C2 - C0: Channel Selections
PD1 - 0: Power-Down Selection
  X: Unused

See Table I for a power-down selection summary.

See Table II for a channel selection control summary.

PD1	PD0	DESCRIPTION
0	0	Power Down Between A/D Converter Conversions
0	1	Internal Reference OFF and A/D Converter ON
1	0	Internal Reference ON and A/D Converter OFF
1	1	Internal Reference ON and A/D Converter ON

TABLE I. Power-Down Selection

CHANNEL SELECTION CONTROL												
SD	C2	C1	C0	CH0	CH1	CH2	CH3	CH4	CH5	CH6	CH7	COM
0	0	0	0	+IN	–IN	—	—	—	—	—	—	—
0	0	0	1	—	—	+IN	–IN	—	—	—	—	—
0	0	1	0	—	—	—	—	+IN	–IN	—	—	—
0	0	1	1	—	—	—	—	—	—	+IN	–IN	—
0	1	0	0	–IN	+IN	—	—	—	—	—	—	—
0	1	0	1	—	—	–IN	+IN	—	—	—	—	—
0	1	1	0	—	—	—	—	–IN	+IN	—	—	—
0	1	1	1	—	—	—	—	—	—	–IN	+IN	—
1	0	0	0	+IN	—	—	—	—	—	—	—	–IN
1	0	0	1	—	—	+IN	—	—	—	—	—	–IN
1	0	1	0	—	—	—	—	+IN	—	—	—	–IN
1	0	1	1	—	—	—	—	—	—	+IN	—	–IN
1	1	0	0	—	+IN	—	—	—	—	—	—	–IN
1	1	0	1	—	—	—	+IN	—	—	—	—	–IN
1	1	1	0	—	—	—	—	—	+IN	—	—	–IN
1	1	1	1	—	—	—	—	—	—	—	+IN	–IN

TABLE II. Channel Selection Control Addressed by Command Byte.

INITIATING CONVERSION

Provided the master has write-addressed it, the ADS7828 turns on the A/D converter's section and begins conversions when it receives BIT 4 of the command byte shown in the Command Byte. If the command byte is correct, the ADS7828 will return an ACK condition.

READING DATA

Data can be read from the ADS7828 by read-addressing the part (LSB of address byte set to 1) and receiving the transmitted bytes. Converted data can only be read from the ADS7828 once a conversion has been initiated as described in the preceding section.

Each 12-bit data word is returned in two bytes, as shown below, where D11 is the MSB of the data word, and D0 is the LSB. Byte 0 is sent first, followed by Byte 1.

	MSB	6	5	4	3	2	1	LSB
**BYTE 0**	0	0	0	0	D11	D10	D9	D8
**BYTE 1**	D7	D6	D5	D4	D3	D2	D1	D0

READING IN F/S MODE

Figure 3 describes the interaction between the master and the slave ADS7828 in Fast or Standard (F/S) mode. At the end of reading conversion data the ADS7828 can be issued a repeated START condition by the master to secure bus operation for subsequent conversions of the A/D converter. This would be the most efficient way to perform continuous conversions.

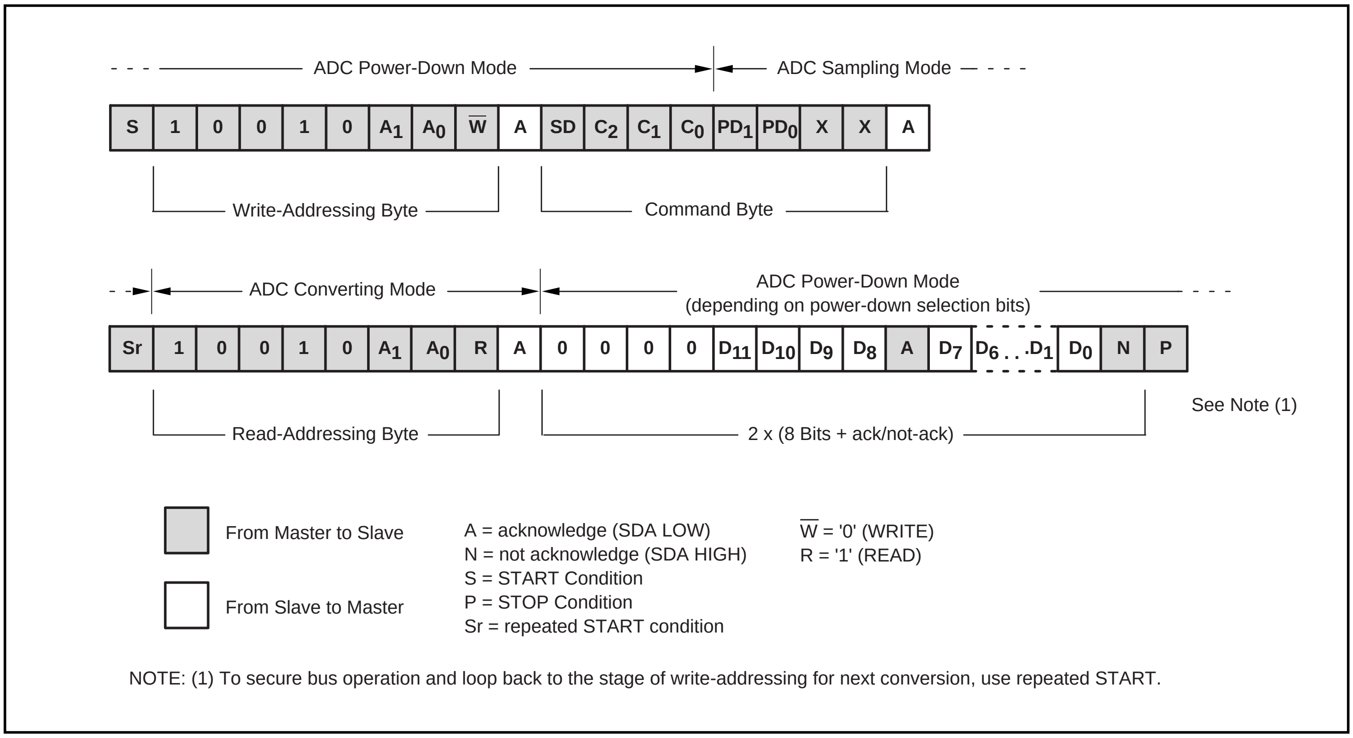

FIGURE 3. Typical Read Sequence in F/S Mode.

READING IN HS MODE

High Speed (HS) mode is fast enough that codes can be read out one at a time. In HS mode, there is not enough time for a single conversion to complete between the reception of a repeated START condition and the read-addressing byte, so the ADS7828 stretches the clock after the read-addressing byte has been fully received, holding it LOW until the conversion is complete.

See Figure 4 for a typical read sequence for HS mode. Included in the read sequence is the shift from F/S to HS modes. It may be desirable to remain in HS mode after reading a conversion; to do this, issue a repeated START instead of a STOP at the end of the read sequence, since a STOP causes the part to return to F/S mode.

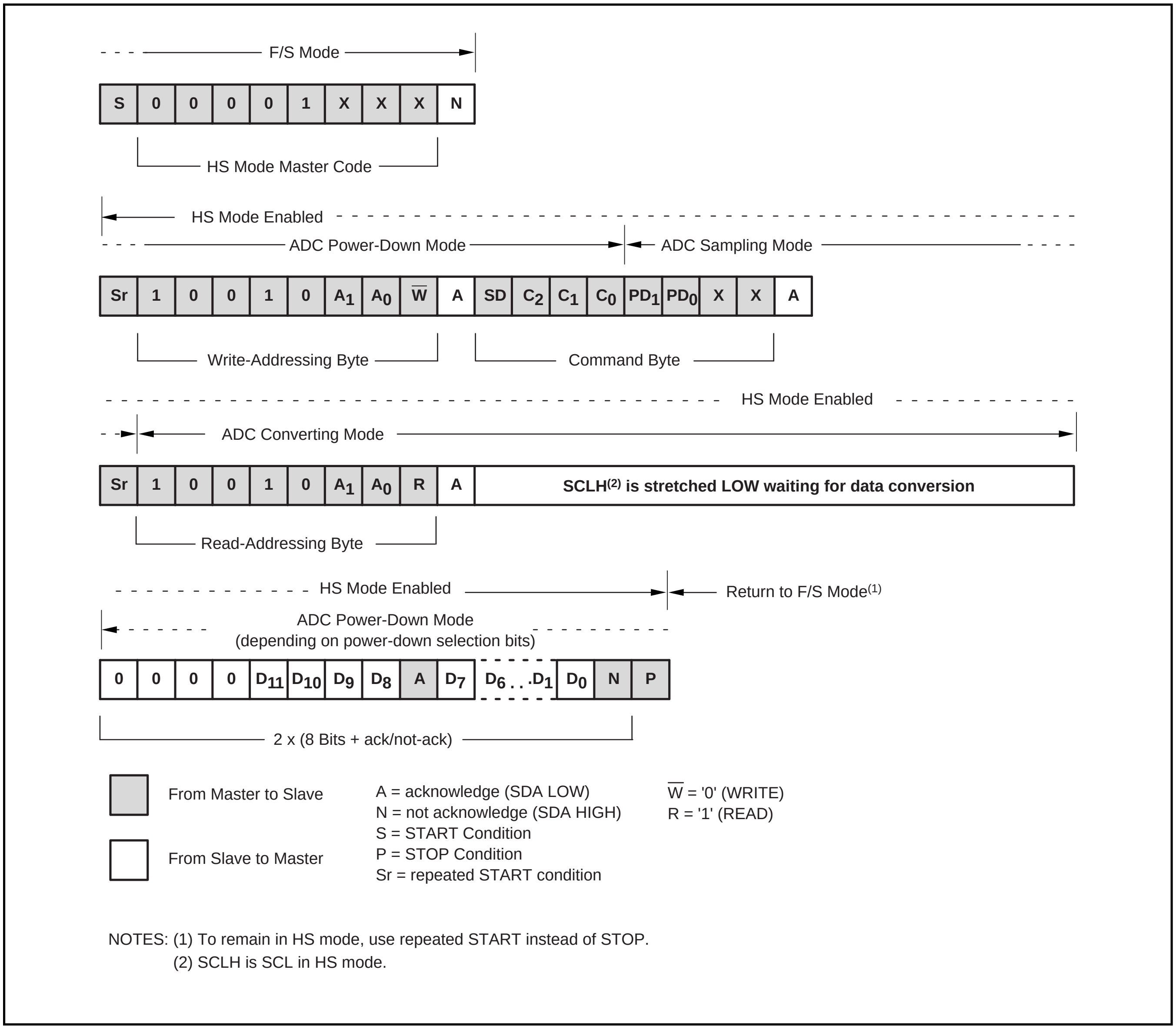

FIGURE 4. Typical Read Sequence in HS Mode.

READING WITH REFERENCE ON/OFF

The internal reference defaults to off when the ADS7828 power is on. To turn the internal reference on or off, see Table I. If the reference (internal or external) is constantly turned on and off, a proper amount of settling time must be added before a normal conversion cycle can be started. The exact amount of settling time needed varies depending on the configuration.

See Figure 5 for an example of the proper internal reference turn-on sequence before issuing the typical read sequences required for the F/S mode when an internal reference is used.

When using an internal reference, there are three things that must be done:

1) In order to use the internal reference, the PD1 bit of Command Byte must always be set to logic ‘1’ for each sample conversion that is issued by the sequence, as shown in Figure 3.

2) In order to achieve 12-bit accuracy conversion when using the internal reference, the internal reference settling time must be considered, as shown in the *Internal V_{REF} vs Turn-On Time* Typical Characteristic plot. If the PD1 bit has been set to logic ‘0’ while using the ADS7828, then the settling time must be reconsidered after PD1 is set to logic ‘1’. In other words, whenever the internal reference is turned on after it has been turned off, the settling time must be long enough to get 12-bit accuracy conversion.

3) When the internal reference is off, it is not turned on until both the first Command Byte with PD1 = ‘1’ is sent *and then* a STOP condition or repeated START condition is issued. (The actual turn-on time occurs once the STOP or repeated START condition is issued.) Any Command Byte with PD1 = ‘1’ issued after the internal reference is turned on serves only to keep the internal reference on. Otherwise, the internal reference would be turned off by any Command Byte with PD1 = ‘0’.

The example in Figure 5 can be generalized for a HS mode conversion cycle by simply swapping the timing of the conversion cycle.

If using an external reference, PD1 must be set to ‘0’, and the external reference must be settled. The typical sequence in Figure 3 or Figure 4 can then be used.

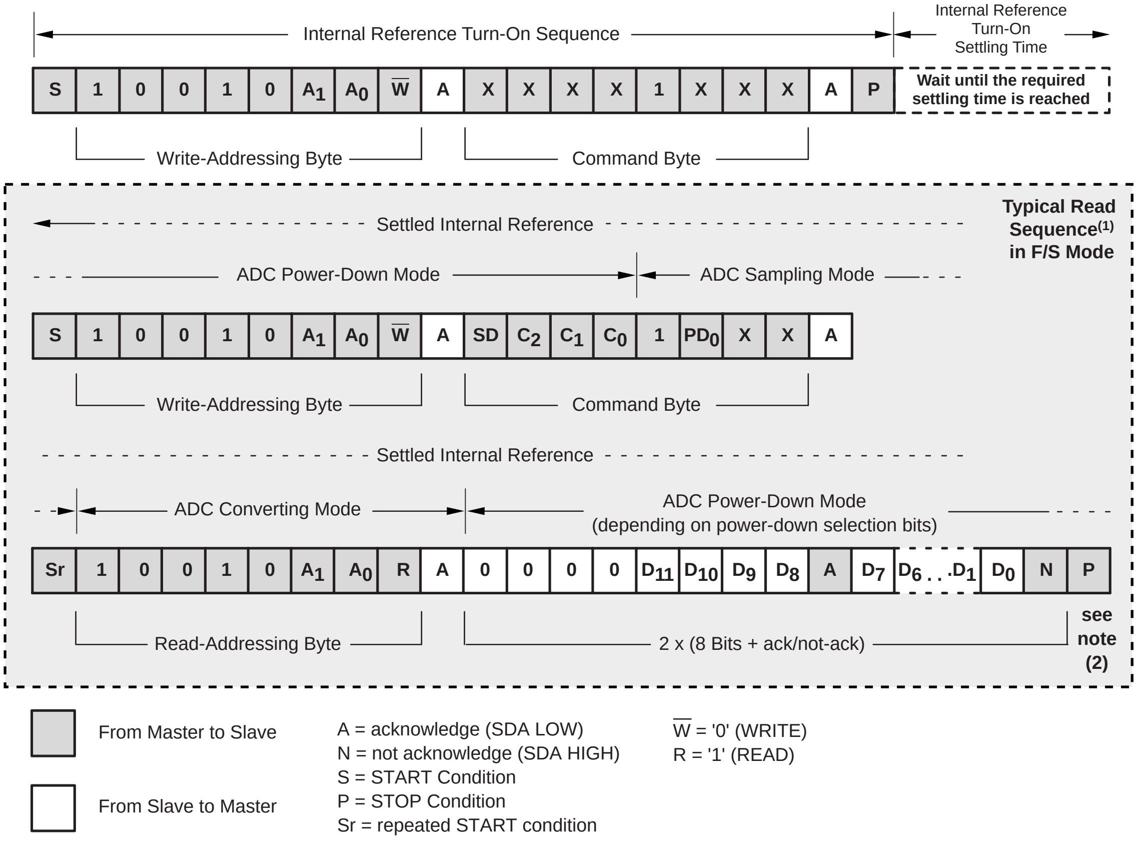

NOTES: (1) Typical read sequences can be reused after the internal reference is settled.
(2) To secure bus operation and loop back to the stage of write-addressing for next conversion, use repeated START.

FIGURE 5. Internal Reference Turn-On Sequence and Typical Read Sequence (F/S mode shown).

LAYOUT

For optimum performance, care should be taken with the physical layout of the ADS7828 circuitry. The basic SAR architecture is sensitive to glitches or sudden changes on the power supply, reference, ground connections, and digital inputs that occur just prior to latching the output of the analog comparator. Therefore, during any single conversion for an “n-bit” SAR converter, there are n “windows” in which large external transient voltages can easily affect the conversion result. Such glitches might originate from switching power supplies, nearby digital logic, and high-power devices.

With this in mind, power to the ADS7828 should be clean and well-bypassed. A 0.1μF ceramic bypass capacitor should be placed as close to the device as possible. A 1μF to 10μF capacitor may also be needed if the impedance of the connection between $+V_{DD}$ and the power supply is high.

The ADS7828 architecture offers no inherent rejection of noise or voltage variation in regards to using an external reference input. This is of particular concern when the reference input is tied to the power supply. Any noise and ripple from the supply will appear directly in the digital results. While high-frequency noise can be filtered out, voltage variation due to line frequency (50Hz or 60Hz) can be difficult to remove.

The GND pin should be connected to a clean ground point. In many cases, this will be the “analog” ground. Avoid connections that are too near the grounding point of a microcontroller or digital signal processor. The ideal layout will include an analog ground plane dedicated to the converter and associated analog circuitry.

PACKAGING INFORMATION

Orderable Device	Status (1)	Package Type	Package Drawing	Pins	Package Qty	Eco Plan (2)	Lead/Ball Finish	MSL Peak Temp (3)
ADS7828E/250	ACTIVE	TSSOP	PW	16	250	Green (RoHS & no Sb/Br)	CU NIPDAU	Level-2-260C-1 YEAR
ADS7828E/250G4	ACTIVE	TSSOP	PW	16	250	Green (RoHS & no Sb/Br)	CU NIPDAU	Level-2-260C-1 YEAR
ADS7828E/2K5	ACTIVE	TSSOP	PW	16	2500	Green (RoHS & no Sb/Br)	CU NIPDAU	Level-2-260C-1 YEAR
ADS7828E/2K5G4	ACTIVE	TSSOP	PW	16	2500	Green (RoHS & no Sb/Br)	CU NIPDAU	Level-1-260C-UNLIM
ADS7828EB/250	ACTIVE	TSSOP	PW	16	250	Green (RoHS & no Sb/Br)	CU NIPDAU	Level-1-260C-UNLIM
ADS7828EB/250G4	ACTIVE	TSSOP	PW	16	250	Green (RoHS & no Sb/Br)	CU NIPDAU	Level-1-260C-UNLIM
ADS7828EB/2K5	ACTIVE	TSSOP	PW	16	2500	Green (RoHS & no Sb/Br)	CU NIPDAU	Level-2-260C-1 YEAR
ADS7828EB/2K5G4	ACTIVE	TSSOP	PW	16	2500	Green (RoHS & no Sb/Br)	CU NIPDAU	Level-2-260C-1 YEAR

(1) The marketing status values are defined as follows:
ACTIVE: Product device recommended for new designs.
LIFEBUY: TI has announced that the device will be discontinued, and a lifetime-buy period is in effect.
NRND: Not recommended for new designs. Device is in production to support existing customers, but TI does not recommend using this part in a new design.
PREVIEW: Device has been announced but is not in production. Samples may or may not be available.
OBSOLETE: TI has discontinued the production of the device.

(2) Eco Plan - The planned eco-friendly classification: Pb-Free (RoHS), Pb-Free (RoHS Exempt), or Green (RoHS & no Sb/Br) - please check http://www.ti.com/productcontent for the latest availability information and additional product content details.
TBD: The Pb-Free/Green conversion plan has not been defined.
Pb-Free (RoHS): TI's terms "Lead-Free" or "Pb-Free" mean semiconductor products that are compatible with the current RoHS requirements for all 6 substances, including the requirement that lead not exceed 0.1% by weight in homogeneous materials. Where designed to be soldered at high temperatures, TI Pb-Free products are suitable for use in specified lead-free processes.
Pb-Free (RoHS Exempt): This component has a RoHS exemption for either 1) lead-based flip-chip solder bumps used between the die and package, or 2) lead-based die adhesive used between the die and leadframe. The component is otherwise considered Pb-Free (RoHS compatible) as defined above.
Green (RoHS & no Sb/Br): TI defines "Green" to mean Pb-Free (RoHS compatible), and free of Bromine (Br) and Antimony (Sb) based flame retardants (Br or Sb do not exceed 0.1% by weight in homogeneous material)

(3) MSL, Peak Temp. -- The Moisture Sensitivity Level rating according to the JEDEC industry standard classifications, and peak solder temperature.

Important Information and Disclaimer: The information provided on this page represents TI's knowledge and belief as of the date that it is provided. TI bases its knowledge and belief on information provided by third parties, and makes no representation or warranty as to the accuracy of such information. Efforts are underway to better integrate information from third parties. TI has taken and continues to take reasonable steps to provide representative and accurate information but may not have conducted destructive testing or chemical analysis on incoming materials and chemicals. TI and TI suppliers consider certain information to be proprietary, and thus CAS numbers and other limited information may not be available for release.

In no event shall TI's liability arising out of such information exceed the total purchase price of the TI part(s) at issue in this document sold by TI to Customer on an annual basis.

PW (R-PDSO-G)** **PLASTIC SMALL-OUTLINE PACKAGE**

14 PINS SHOWN

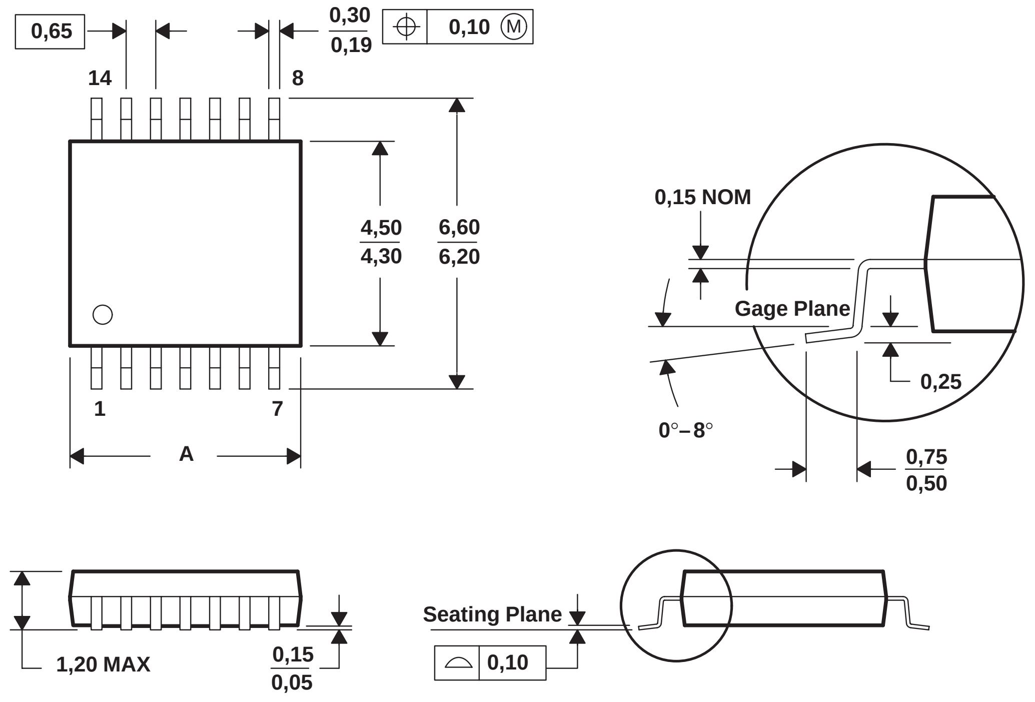

PINS ** / DIM	8	14	16	20	24	28
A MAX	3,10	5,10	5,10	6,60	7,90	9,80
A MIN	2,90	4,90	4,90	6,40	7,70	9,60

4040064/F 01/97

NOTES: A. All linear dimensions are in millimeters.
B. This drawing is subject to change without notice.
C. Body dimensions do not include mold flash or protrusion not to exceed 0,15.
D. Falls within JEDEC MO-153

IMPORTANT NOTICE

Texas Instruments Incorporated and its subsidiaries (TI) reserve the right to make corrections, modifications, enhancements, improvements, and other changes to its products and services at any time and to discontinue any product or service without notice. Customers should obtain the latest relevant information before placing orders and should verify that such information is current and complete. All products are sold subject to TI's terms and conditions of sale supplied at the time of order acknowledgment.

TI warrants performance of its hardware products to the specifications applicable at the time of sale in accordance with TI's standard warranty. Testing and other quality control techniques are used to the extent TI deems necessary to support this warranty. Except where mandated by government requirements, testing of all parameters of each product is not necessarily performed.

TI assumes no liability for applications assistance or customer product design. Customers are responsible for their products and applications using TI components. To minimize the risks associated with customer products and applications, customers should provide adequate design and operating safeguards.

TI does not warrant or represent that any license, either express or implied, is granted under any TI patent right, copyright, mask work right, or other TI intellectual property right relating to any combination, machine, or process in which TI products or services are used. Information published by TI regarding third-party products or services does not constitute a license from TI to use such products or services or a warranty or endorsement thereof. Use of such information may require a license from a third party under the patents or other intellectual property of the third party, or a license from TI under the patents or other intellectual property of TI.

Reproduction of information in TI data books or data sheets is permissible only if reproduction is without alteration and is accompanied by all associated warranties, conditions, limitations, and notices. Reproduction of this information with alteration is an unfair and deceptive business practice. TI is not responsible or liable for such altered documentation.

Resale of TI products or services with statements different from or beyond the parameters stated by TI for that product or service voids all express and any implied warranties for the associated TI product or service and is an unfair and deceptive business practice. TI is not responsible or liable for any such statements.

Following are URLs where you can obtain information on other Texas Instruments products and application solutions:

**Products**		**Applications**	
Amplifiers	amplifier.ti.com	Audio	www.ti.com/audio
Data Converters	dataconverter.ti.com	Automotive	www.ti.com/automotive
DSP	dsp.ti.com	Broadband	www.ti.com/broadband
Interface	interface.ti.com	Digital Control	www.ti.com/digitalcontrol
Logic	logic.ti.com	Military	www.ti.com/military
Power Mgmt	power.ti.com	Optical Networking	www.ti.com/opticalnetwork
Microcontrollers	microcontroller.ti.com	Security	www.ti.com/security
Low Power Wireless	www.ti.com/lpw	Telephony	www.ti.com/telephony
		Video & Imaging	www.ti.com/video
		Wireless	www.ti.com/wireless

Mailing Address: Texas Instruments
Post Office Box 655303 Dallas, Texas 75265

Copyright © 2006, Texas Instruments Incorporated
