# Supplementary material for: In-home and remote use of robotic body surrogates by people with profound motor deficits
Source: PLoS One. 2019 Mar 15;14(3):e0212904. doi: 10.1371/journal.pone.0212904 (PMC6420017; doi:10.1371/journal.pone.0212904)
Supplement: S1 Source Code — This includes all custom code used in the described system and related evaluations. (ZIP) [file pone.0212904.s009.zip › robotic_body_surrogates_source_code/hrl-haptic-manip-pkgs.zip!/hrl-haptic-manip-pkgs/hrl_fabric_based_tactile_sensor/arduino_sketchbook/AN4077.pdf]

MMA845xQ Design Checklist and Board Mounting Guidelines

by: Kimberly Tuck
Applications Engineer

1.0 Introduction

This document is intended to assist customers with the design-in of the MMA845xQ 3-axis low-g consumer grade accelerometers. This document points out the similarities of the pinout for these devices with a recommended layout for implementing one board compatible for all devices. Guidelines for board mounting to a PCB are given with recommendations for I^2C communication and speeds.

1.1 Key Words

Accelerometer, Board Mounting, Solder Paste, Printed Circuit Board (PCB), I^2C Communication, Pull-up Resistor, Pull-down Resistor, Sensor, I/O pins, Non Solder Mask Defined, Solder Mask, Land Pattern, Stencil, Halogen Free Package, RoHS Compliant, Level translator, QFN, Bypass Capacitor, MMA8450Q, MMA8451Q, MMA8452Q, MMA8453Q

TABLE OF CONTENTS

1.0 Introduction 1
1.1 Key Words 1
2.0 MMA845xQ Consumer 3-axis Accelerometer 3 x 3 x 1 mm 2
2.1 Brief Product Sensitivity and g-range Descriptions 3
2.1.1 MMA8450Q 3
2.1.2 MMA8451Q 3
2.1.3 MMA8452Q 3
2.1.4 MMA8453Q Note: No HPF Data 3
3.0 Pin Connections to the MMA845XQ 3
4.0 Board Layout 5
4.1 Power Supply Decoupling 5
4.2 Level Translators 5
4.3 I/O Pins 5
4.4 Sensor Placement 6
5.0 I^2C Communication 6
6.0 Offset Calibration 6
7.0 Mounting Guidelines for the Quad Flat No Lead (QFN) Package 6
7.1 Overview of Soldering Considerations 6
7.2 Halogen Content 6
7.3 PCB Mounting Recommendations 7

© 2010-2012 Freescale Semiconductor, Inc. All rights reserved.

2.0 MMA845xQ Consumer 3-axis Accelerometer 3 x 3 x 1 mm

The MMA8451, 2, 3Q has a selectable dynamic range of ±2g, ±4g, ±8g. The device has 8 different output data rates, selectable high pass filter cut-off frequencies, and high pass filtered data. The available resolution of the data and the embedded features is dependant on the specific device.

Note: The MMA8450Q has a different memory map and has a slightly different pin-out configuration.

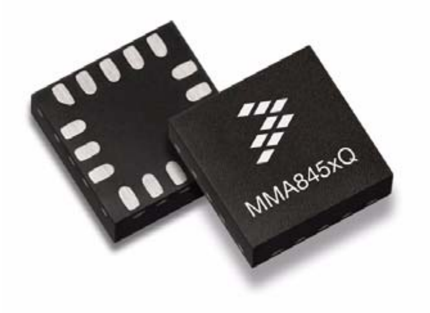

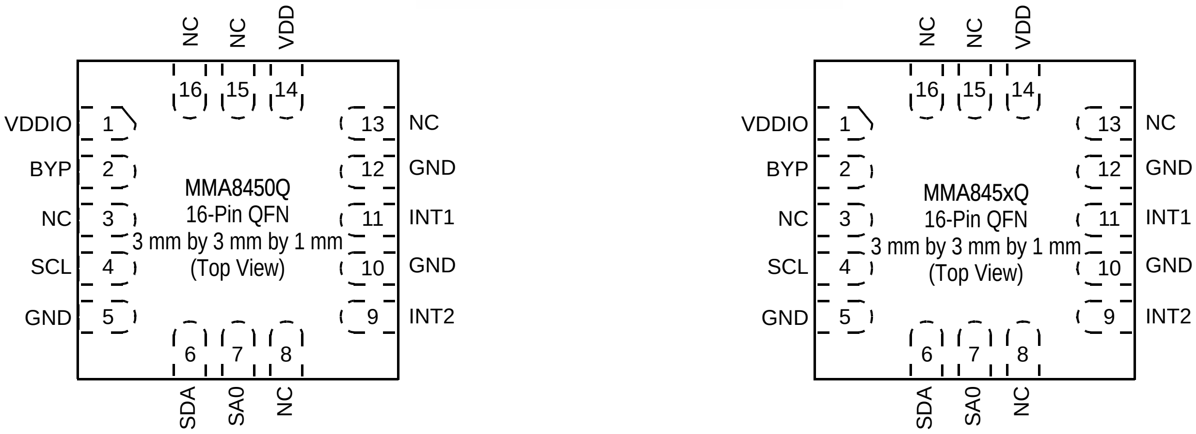

Figure 1. Device Pinouts for the MMA8450Q and the MMA845xQ

2.1 Brief Product Sensitivity and g-range Descriptions

2.1.1 MMA8450Q

1. **12-bit data**
   2g (1024 counts/g = 1 mg/LSB) **4g** (512 counts/g = 2 mg/LSB) **8g** (256 counts/g = 3.9 mg/LSB)
2. **8-bit data**
   2g (64 counts/g = 15.6 mg/LSB) **4g** (32 counts/g =31.25 mg/LSB) **8g** (16 counts/g = 62.5 mg/LSB)
   Embedded 32 sample FIFO (Fill and Circular Modes) Regular Data Only- no HPF in FIFO

2.1.2 MMA8451Q

1. **14-bit data**
   2g (4096 counts/g = 0.25 mg/LSB) **4g** (2048 counts/g = 0.5 mg/LSB) **8g** (1024 counts/g = 1 mg/LSB)
2. **8-bit data**
   2g (64 counts/g = 15.6 mg/LSB) **4g** (32 counts/g = 31.25mg/LSB) **8g** (16 counts/g = 62.5 mg/LSB)
   Embedded 32 sample FIFO (Fill, Circular and Trigger Modes) Regular Data and HPF Data in FIFO

2.1.3 MMA8452Q

1. **12-bit data**
   2g (1024 counts/g = 1 mg/LSB) **4g** (512 counts/g = 2 mg/LSB) **8g** (256 counts/g = 3.9 mg/LSB)
2. **8-bit data**
   2g (64 counts/g = 15.6 mg/LSB) **4g** (32 counts/g = 31.2 5 mg/LSB) **8g** (16 counts/g = 62.5 mg/LSB)

2.1.4 MMA8453Q Note: No HPF Data

1. **10-bit data**
   2g (256 counts/g 3.9mg/LSB) **4g** (128 counts/g = 7.8mg/LSB) **8g** (64 counts/g= 15.6 mg/LSB)
2. **8-bit data**
   2g (64 counts/g = 15.6 mg/LSB) **4g** (32 counts/g = 31.25 mg/LSB) **8g** (16 counts/g = 62.5 mg/LSB)

3.0 Pin Connections to the MMA845XQ

Table 1 describes each pin of the MMA8450Q, the MMA8451, 2, 3Q with recommended connections.

Table 1. Pinout Comparison MMA8450Q, MMA8451,2,3Q

Pin#	MMA8450Q	Recommendations for MMA8450Q	MMA8451Q MMA8452Q MMA8453Q	Recommendation for MMA8451, 2, 3Q
1	VDDIO	Connect to Pin 14 (1.71 V - 1.89 V)	VDDIO	1.62 V - 3.6 V
2	NC	NC, GND or Bypass Cap to GND	BYPASS	0.1μF Bypass Cap to Ground
3	NC	Leave unconnected or GND	NC	MUST leave unconnected
4	SCL	Pull-up Resistor (4.7 kΩ typical) required on I^2C bus	SCL	Pull-up Resistor (4.7 kΩ typical) required on I^2C bus
5	GND	Connect to Ground	GND	Connect to Ground
6	SDA	Pull-up Resistor (4.7 kΩ typical) required on I^2C bus	SDA	Pull-up Resistor (4.7 kΩ typical) required on I^2C bus
7	SA0	Connect to Ground for I^2C Address $1C. Connect to VDD for I^2C Address $1D	SA0	Connect to Ground for I^2C Address $1C. Connect to VDD for I^2C Address $1D.
8	EN	Connect to MCU	NC	Leave unconnected, connect to VDD or GND
9	INT2	Connect to MCU interrupt	INT2	Connect to MCU interrupt
10	GND	Connect to Ground	GND	Connect to Ground
11	INT1	Connect to MCU interrupt	INT1	Connect to MCU interrupt
12	GND	Connect to Ground	GND	Connect to Ground
13	GND	Connect to Ground	NC	Leave unconnected, connect to VDD or GND
14	VDD	Connect to Pin 1 (1.71 V - 1.89 V)	VDD	Analog Input 1.95 V - 3.6 V
15	NC	Leave unconnected, connect to VDD or GND	NC	Leave unconnected, connect to VDD or GND
16	NC	Leave unconnected, connect to VDD or GND	NC	Leave unconnected, connect to VDD or GND

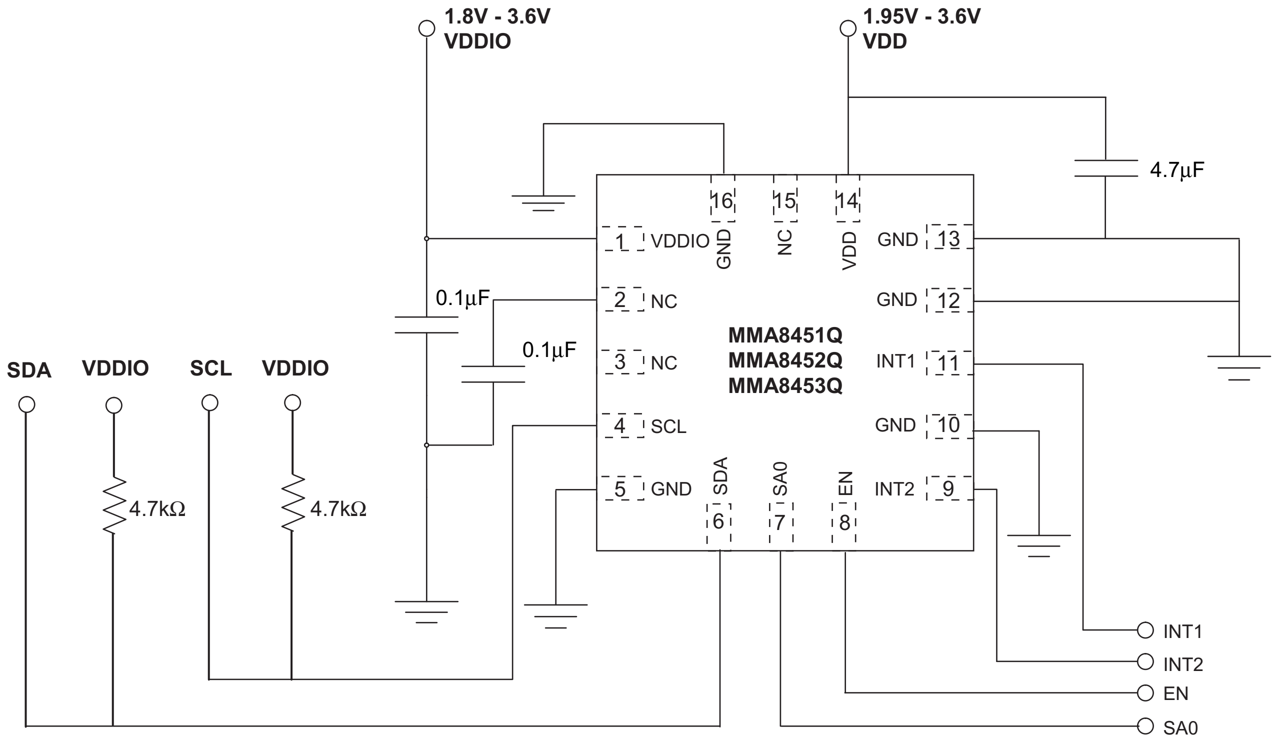

Figure 2. Recommended Schematic for the MMA8451, 2, 3Q

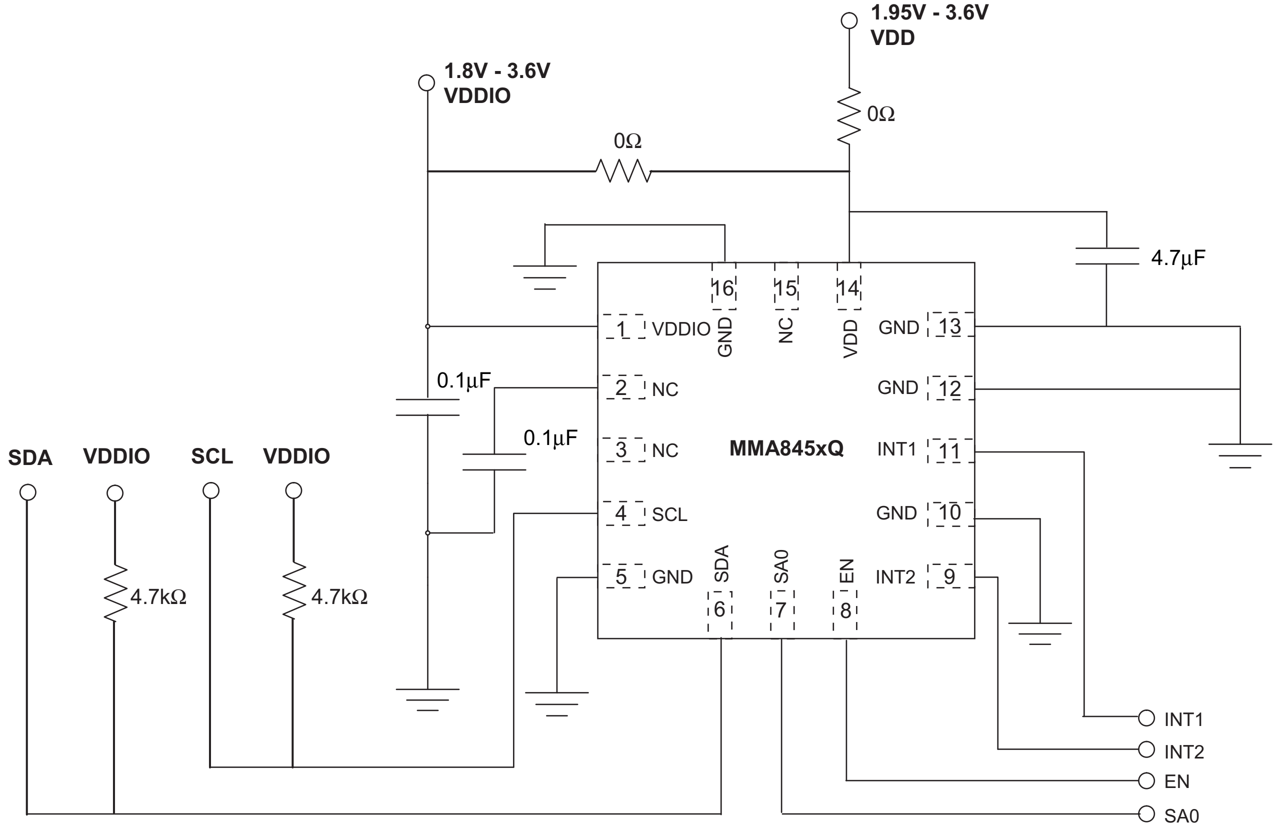

Figure 3. Schematic for either MMA8450Q, or MMA8451, 2, 3Q

4.0 Board Layout

The following are some important considerations for board layout of the MMA845xQ accelerometers to ensure the best performance. Level translators may be required only if the MCU/processor is operating at a higher voltage. Bypass capacitors are recommended on the input voltage pins to ensure a clean signal to the sensor for optimal noise performance. Figure 4 is an example of Freescale's MMA8450Q evaluation board. This is a four (4) layer board. The board was designed to be able to access every pin on both the MCU and on the accelerometer for quick debug. The board has 32Mbit Flash which can be used for data logging and to perform algorithm analysis. The board has an LED and a switch for general purpose functions and a resistor, "R3", that can be cut to measure current drain from the accelerometer. This board was designed as small as possible so that it would be non invasive when mounted on other boards for different applications. The board can plug into an interface board to the PC or to a battery board. The flat back of the board is great for mounting the board with the battery pack onto moving objects to log data. The flat back also allows the board to be easily mounted into other designs. All the accelerometer pins are accessible through the board, bypassing the MC9S08QE8 microcontroller, if another external processor/MCU will be used.

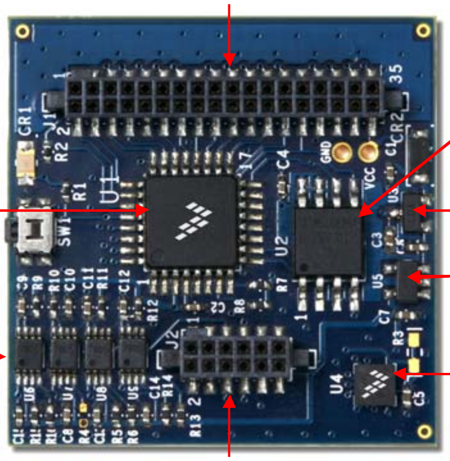

Figure 4. MMA8450Q Example Evaluation Board

4.1 Power Supply Decoupling

A 4.7 μF capacitor is recommended to filter noise in case the input supply is noisy, and a 0.1 μF bypass capacitor is recommended to filter out high frequency noise. Place these capacitors as close to the part as possible. The caps should be placed between power and ground as shown in the schematics.

4.2 Level Translators

As shown from the MMA8450Q Evaluation board in Figure 4, level translators are required because the MCU is operating at 3.3 V and the operating voltage of the MMA8450Q is limited to 1.8 V. The SCL, SDA, SA0, EN, INT1 and INT2 signals will shift through the level translator when communicating back and for to the MCU. Level translators on this board are not required for the other family members of the MMA845xQ (MMA8451, 2, 3Q) since the operating voltage can be regulated at 3.3 V which matches the voltage of the MCU on this board.

4.3 I/O Pins

Pin 7 (SA0: Address Pin), Pin 9 (INT2), Pin 11 (INT1), Pin 6 (SDA), Pin 4 (SCL) and Pin 8 (EN) should all use pull-up/down resistors to ensure they maintain the state of the pin. The interrupt pins can be configured to be push-pull or open drain and can be either Active High or Active Low. If a pull-up resistor is used on the interrupt pins with the open drain setting, then the accelerometer should be configured to Active Low. If a pull-down resistor is used, then the interrupt pin should be configured to be Active High. This is done in Register 0x2C (MMA8451, 2, 3Q). Bit 0 sets the configuration of the interrupt pins to be open drain or push pull-and Bit 1 sets the polarity, either Active High or Active Low. Note the MMA8450Q register map is different and the interrupt configuration settings are held in Register 0x3A.

4.4 Sensor Placement

Sensor placement is very important and is often overlooked. The MEMS sensor inside the package is very sensitive to stresses. Small deflections inside the MEMS sensor on the order of 10 nm correspond to a change in acceleration of 1g. Care must be taken to ensure that the package is not stressed by holes or components on the PCB placed too closely to the accelerometer. It is important to place the sensor where it is NOT vulnerable to be pushed or otherwise affected directly by the user's hands. Also avoid bending the PCB containing the sensor as the PCB stress is transferred to the accelerometer. Temperature can also be an issue. It is good to avoid placing the sensor near components that may have large temperature variations, or that are constantly very hot as this will affect the offset of the sensor. For optimal motion detection, place the sensor away from the center of the device. This will ensure better acceleration readings and make them more significant to detect smaller motions, from a higher moment of inertia than if placed right on the center of movement.

5.0 I^2C Communication

The MMA845xQ, I^2C communication protocol follows the Philips Semiconductors (now NXP Semiconductors) standard. In this interface, only two bus lines are required: a serial data line (SDA) and a serial clock line (SCL). Serial, 8-bit oriented bidirectional data transfers can be made at up to 100 kbit/s in the standard mode and up to 400 kbit/s in the fast mode. The maximum allowable bus capacitance is 400 pF. Both SDA and SCL are bidirectional lines, connected to a positive supply voltage via a pull-up resistor. The recommended value is between 1k - 4.7k. The maximum I^2C speed with 1k pull-up resistor is 4.75 MHz and with 4.7k pull-up is 2.25 MHz. The accelerometer is always considered the slave and the MCU is always considered the master.

The benefits of the I^2C communication interface is that many ICs can be added to this bus. The only limitation is the bus capacitance. The simple 2-wire serial I^2C-bus minimizes interconnections so ICs have fewer pins and there are not as many PCB traces, resulting in smaller and less expensive PCBs. Each device is recognized by a unique address (whether it is a microcontroller, memory or an accelerometer). The MMA845xQ has an extra address bit to allow for two different addresses available using Pin 7 (SA0). When Pin 7 is high, the 7-bit I^2C address is 0x1D. When Pin 7 is low, the 7-bit I^2C address is 0x1C.

6.0 Offset Calibration

After the accelerometer has been mounted onto a PCB there may be a small offset shift. If additional calibration is required there are three registers to zero out the offset.

7.0 Mounting Guidelines for the Quad Flat No Lead (QFN) Package

Surface mount board layout is a critical portion of the total design. The footprint for the surface mount packages must be the correct size to ensure proper solder connection interface between the board and the package. With the correct footprint, the packages will self-align when subjected to a solder reflow process. It is always recommended to design boards with a solder mask layer to avoid bridging and shorting between solder pads.

These guidelines are for soldering and mounting the Quad Flat No-Lead (QFN) package inertial sensors to Printed Circuit Boards (PCBs). The purpose is to minimize the stress on the package after board mounting. The MMA845xQ digital output accelerometers use the QFN package platform. This section describes suggested methods of soldering these devices to the PC board for consumer applications.

7.1 Overview of Soldering Considerations

Information provided here is based on experiments executed on QFN devices. They do not represent exact conditions present at a customer site. Hence, information herein should be used as guidance only and process and design optimizations are recommended to develop an application specific solution. It should be noted that with the proper PCB footprint and solder stencil designs, the package will self-align during the solder reflow process.

7.2 Halogen Content

This package is designed to be Halogen Free, exceeding most industry and customer standards. Halogen Free means that no homogeneous material within the assembly package shall contain chlorine (Cl) in excess of 700 ppm or 0.07% weight/weight or bromine (Br) in excess of 900 ppm or 0.09% weight/weight.

7.3 PCB Mounting Recommendations

1. The PCB land should be designed with Non Solder Mask Defined (NSMD) as shown in Figure 5 and Figure 6
2. No additional via pattern underneath package.
3. PCB land pad is 0.8 mm x 0.3 mm as shown in Figure 5 and Figure 6.
4. Solder mask opening = PCB land pad edge + 0.113 mm larger all around.
5. Stencil opening = PCB land pad -0.015 mm smaller all around = 0.77 mm x 0.27 mm.
6. Stencil thickness is 100 to 125 μm.
7. Do not place any components or vias at a distance less than 5 mm from the package land area. This may cause additional package stress if it is too close to the package land area.
8. Signal traces connected to pads are as symmetric as possible. Put dummy traces on NC pads in order to have same length of exposed trace for all pads.
9. Use a standard pick and place process and equipment. Do not use a hand soldering process.
10. It is recommended to use a no clean solder paste.
11. Do not use a screw down or stacking to fix the PCB into an enclosure because this could bend the PCB putting stress on the package.
12. The PCB should be rated for the multiple lead-free reflow condition with max 260°C temperature.
13. No copper traces on top layer of PCB under the package. This will cause planarity issues with board mount. Freescale QFN sensors are compliant with Restrictions on Hazardous Substances (RoHS), having halide free molding compound (green) and lead-free terminations. These terminations are compatible with tin-lead (Sn-Pb) as well as tin-silver-copper (Sn-Ag-Cu) solder paste soldering processes. Reflow profiles applicable to those processes can be used successfully for soldering the devices.

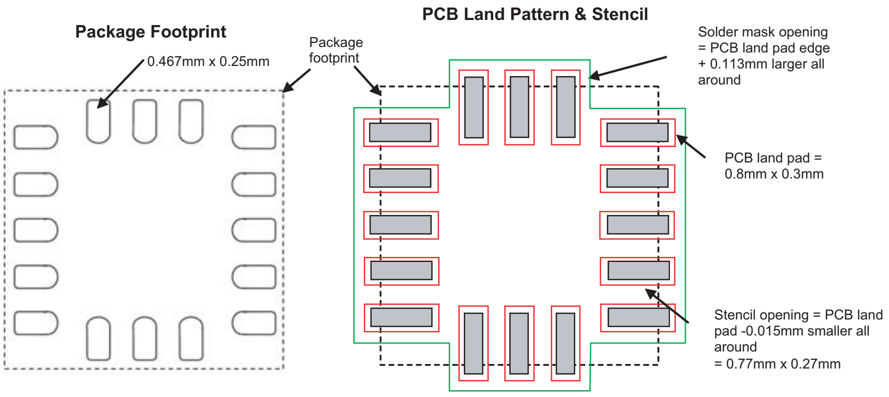

Figure 5. Recommended PCB Land Pad, Solder Mask and Stencil Opening Near Package Footprint

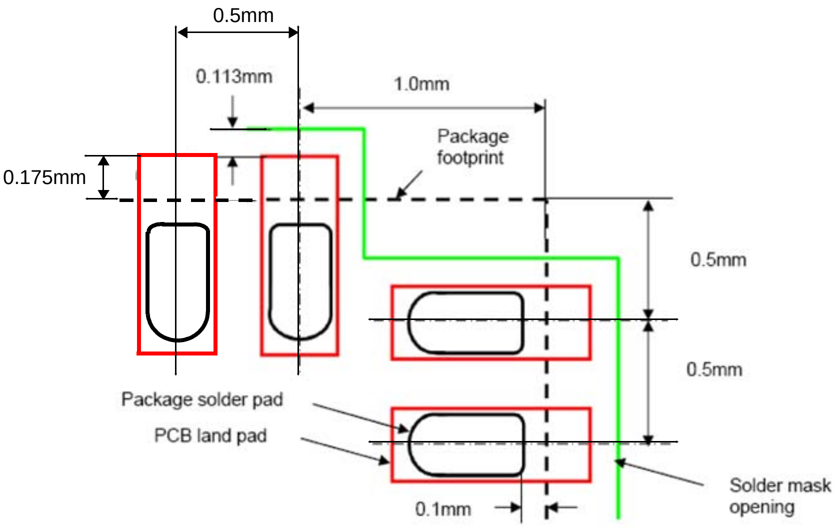

Note: 0.5 mm separation is recommended on both the horizontal and vertical fields.

Figure 6. Detailed Dimensions

Table 2. Revision History

Revision number	Revision date	Description of changes
2	10/2012	• In Figure 6. Detailed Dimensions on page 7, changed dimension from 1.75mm to 0.175.

How to Reach Us:

Home Page:
freescale.com

Web Support:
freescale.com/support

Information in this document is provided solely to enable system and software implementers to use Freescale products. There are no express or implied copyright licenses granted hereunder to design or fabricate any integrated circuits based on the information in this document.

Freescale reserves the right to make changes without further notice to any products herein. Freescale makes no warranty, representation, or guarantee regarding the suitability of its products for any particular purpose, nor does Freescale assume any liability arising out of the application or use of any product or circuit, and specifically disclaims any and all liability, including without limitation consequential or incidental damages. “Typical” parameters that may be provided in Freescale data sheets and/or specifications can and do vary in different applications, and actual performance may vary over time. All operating parameters, including “typicals,” must be validated for each customer application by customer’s technical experts. Freescale does not convey any license under its patent rights nor the rights of others. Freescale sells products pursuant to standard terms and conditions of sale, which can be found at the following address: freescale.com/salestermsandconditions.

Freescale and the Freescale logo are trademarks of Freescale Semiconductor, Inc., Reg. U.S. Pat. & Tm. Off. Xtrinsic is a trademark of Freescale Semiconductor, Inc.

All other product or service names are the property of their respective owners.

© 2012 Freescale Semiconductor, Inc. All rights reserved.

AN4077
Rev. 2
10/2012

freescale™
