# Supplementary material for: In-home and remote use of robotic body surrogates by people with profound motor deficits
Source: PLoS One. 2019 Mar 15;14(3):e0212904. doi: 10.1371/journal.pone.0212904 (PMC6420017; doi:10.1371/journal.pone.0212904)
Supplement: S1 Source Code — This includes all custom code used in the described system and related evaluations. (ZIP) [file pone.0212904.s009.zip › robotic_body_surrogates_source_code/hrl-haptic-manip-pkgs.zip!/hrl-haptic-manip-pkgs/hrl_fabric_based_tactile_sensor/arduino_sketchbook/I2C-ADC-Manual.pdf]

I2C-ADC™ I^2C 12-Bit, 8-CH ANALOG-TO-DIGITAL CONVERTER User Manual

GRAVITECH.US

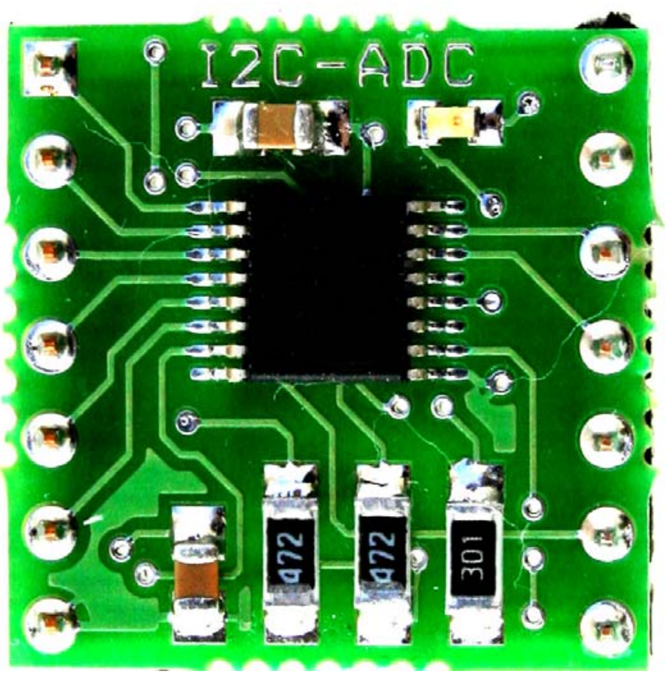

uResearch

GRAVITECH GROUP

Copyright © 2007 MicroResearch
GRAVITECH GROUP WWW.GRAVITECH.US

I2C-ADC™ I²C 12-Bit, 8-CH ANALOG-TO-DIGITAL CONVERTER User Manual

Description

The I2C-ADC board is a 14-pin CMOS device that provides 8-CH, 12-bit of Analog to Digital Converter (ADC) using I²C bus. There are no external components required. Only two signal lines SDA and SCL plus supply voltage and ground are required to be connected. This makes it perfect for embedded systems that require analog-to-digital converter.

This board features innovations that set it apart from other ADC module. Innovations feature like on-board I²C address jumpers, pull-up resistors, power LED and 2.5V reference. The module can be quickly connected directly on to the breadboard. The board is small and compact in size 0.70 x 0.70 inches.

The I2C-ADC is designed base on ADS7828 IC. It is a single-supply, low-power, 12-bit data acquisition device that features a serial I²C interface and an 8-channel multiplexer. The Analog-to-Digital (A/D) converter features a sample-and-hold amplifier and internal, asynchronous clock. The combination of an I²C serial, 2-wire interface and micropower consumption makes the
I2C-ADC ideal for applications requiring the A/D converter to be close to the input source in remote locations and for applications requiring isolation.

Two jumper pins vary the fixed I²C address and allow up to four devices to share the same I²C bus. That is total of 32 channels.

Features

- Stand alone module, no external components required
- On-board I²C address jumpers, pull-up resistors, power LED and 2.5V reference
- Decoupling supply voltage
- Design easy for breadboard
- High quality double sided PCB
- All SMT components
- Small and compact in size 0.70 x 0.70 inches
- Dual row 0.6" width, 0.1" pitch header pins
- Operating power supply voltage range of 2.7V to 5V
- Suitable for 3.3V or 5.0V microcontroller
- 8-Channel multiplexer
- 50kHz sampling rate
- Internal 2.5V reference
- I²C interface supports: Standard, Fast and High-Speed Modes

Applications

- Voltage-supply monitoring
- Isolated data acquisition
- Transducer interfaces
- Battery-operated systems
- Remote data acquisition
- And much more…

* I²C is a trademark of Philips Semiconductors Corporation.

Copyright © 2007 MicroResearch
GRAVITECH GROUP WWW.GRAVITECH.US

I2C-ADC™ I²C 12-Bit, 8-CH ANALOG-TO-DIGITAL CONVERTER User Manual

Pin Configuration

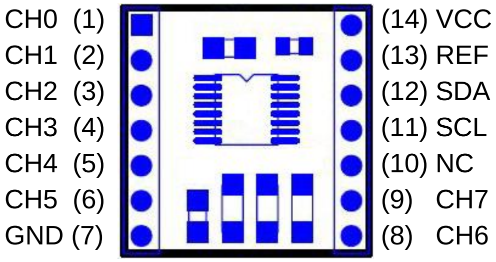

Pin No.	Name	Type	Description
1-6	CH0 – CH5	Input	Analog input channel 0 to 5
7	GND	PWR	Supply ground
8-9	CH6 – CH7	Input	Analog input channel 6 to 7
10	NC	NC	No connect
11	SCL	Input	Serial clock line
12	SDA	I/O	Serial data line
13	REF	Input	External reference input
14	VCC	PWR	Supply voltage

Interfaces

Power:

The I2C-ADC board needs an external 2.7VDC – 5.0VDC supply.

- **VCC:** is an input power 2.7VDC – 5.0VDC to I2C-ADC board.
- **GND:** is a common ground for every pin. This pin MUST be connected to ground of the external power supply.

Analog input pins:

There are eight analog input pins. When the converter enters the hold mode, the voltage on the selected CHx pin is captured on the internal capacitor array. During the sample period, the source must charge the internal sampling capacitor (typically 25pF). The amount of charge transfer from the analog source to the converter is a function of conversion rate.

Copyright © 2007 MicroResearch
GRAVITECH GROUP WWW.GRAVITECH.US

I2C-ADC™ I²C 12-Bit, 8-CH ANALOG-TO-DIGITAL CONVERTER User Manual

REF pin:

The I2C-ADC can operate with an internal 2.5V reference or an external reference. If a +5V supply is used, an external +5V reference is required in order to provide full dynamic range for a 0V to +VDD analog input. This external reference can be as low as 50mV.

I²C pins:

The I2C-ADC operates as a slave on the I²C bus. Only two signal lines SDA and SCL are required for I²C bus. Please refer to I²C specification for more information.

Module Configuration

I²C address:

Default address shipped from the manufacture is 0x90 for write and 0x91 for read.

A1 = 0
A0 = 0

MSB	6	5	4	3	2	1	LSB
1	0	0	1	0	A1	A0	R/$\overline{W}$

The address can be easily change by solder the bridge between the corresponding address pin to VCC or GND at the bottom of the module. These four combinations allow up to four devices to share the same I²C bus. That is total of 32 channels.

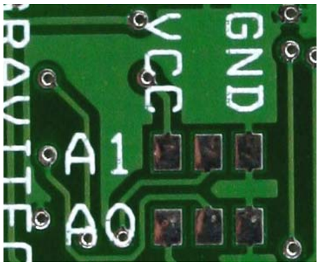

Power-on LED:

The green LED on the module is illuminating when the power applied. The power-on LED is enabled from the manufacture. It can be disabling for light sensitive or low current requirement application by remove the solder bridge on “PW” at the bottom of the module.

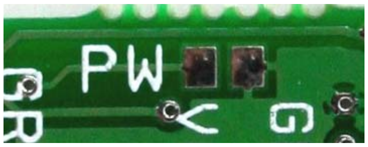

I²C pull-up resistors:

I²C bus specification required to have pull-up resistors on SDA and SCL pin. I2C-ADC come with these two pull-up resistors enabled from the manufacture. It can be disabling when connect to I²C bus that already have pull-up resistors by remove the solder bridge on “PU” at the bottom of the module.

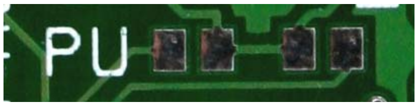

Copyright © 2007 MicroResearch
GRAVITECH GROUP WWW.GRAVITECH.US

Internal/External reference:

The I2C-ADC can operate with an internal 2.5V reference by solder the bridge between the middle pad of EX/IN row to “IN”. An external reference can be select by solder the bridge between the middle pad of EX/IN row to “EX”. It may NOT exceed the supply voltage to the module. The device shipped with an internal reference option from the manufacture.

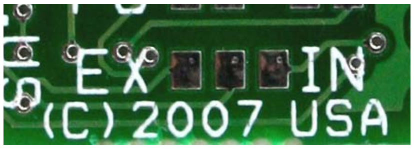

Below is the default setting from the manufacture.

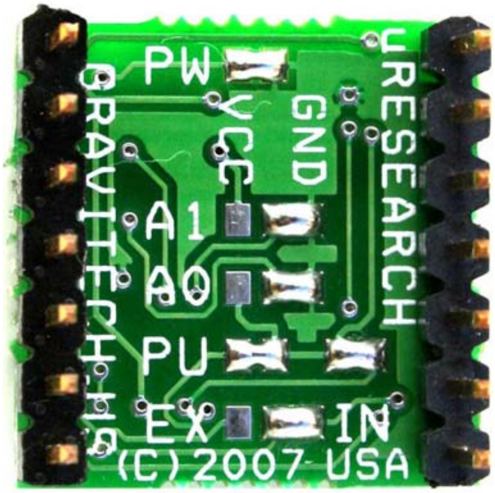

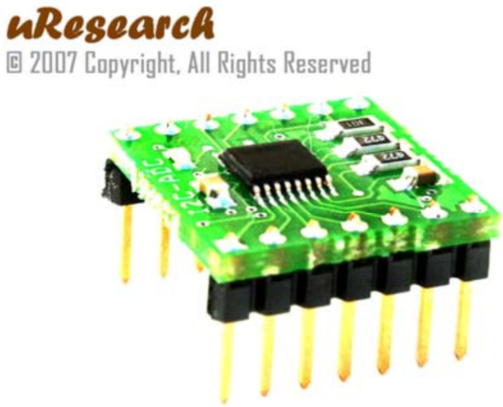

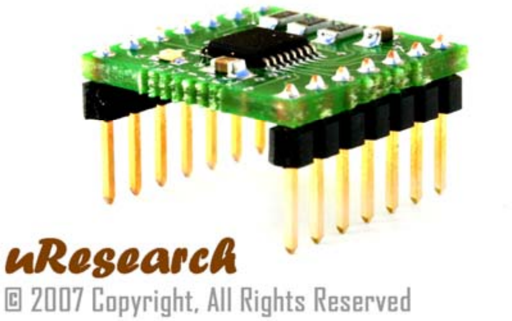

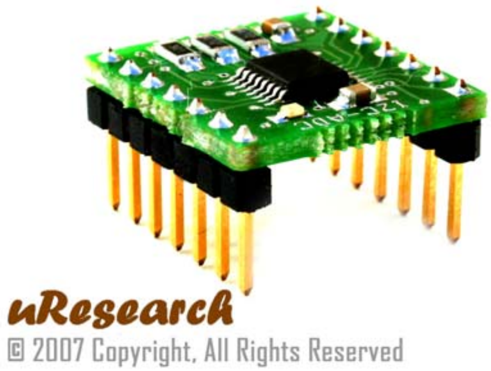

Notes

Contact Us

We maintain a website where you can get information on our products, obtain literature and download support files. Visit us online at:

WWW.GRAVITECH.US

Use our online Forum or e-mail your technical support questions to support@gravitech.us. We try to respond to your questions the same day.

For sales questions or to place and order, direct your e-mails to sales@gravitech.us. Refer to our website for product pricing, shipping rates, payment instructions, and for other info we need to complete your order.

Disclaimer: MicroResearch reserves the right to modify its products or literature, or to discontinue any product at any time without prior notice. The customer is responsible for determining the suitability of any device for any application developed using MicroResearch components.

Copyright © 2007 MicroResearch

GRAVITECH GROUP WWW.GRAVITECH.US
