# Supplementary material for: In-home and remote use of robotic body surrogates by people with profound motor deficits
Source: PLoS One. 2019 Mar 15;14(3):e0212904. doi: 10.1371/journal.pone.0212904 (PMC6420017; doi:10.1371/journal.pone.0212904)
Supplement: S1 Source Code — This includes all custom code used in the described system and related evaluations. (ZIP) [file pone.0212904.s009.zip › robotic_body_surrogates_source_code/hrl-haptic-manip-pkgs.zip!/hrl-haptic-manip-pkgs/hrl_haptic_mpc/docs/html/annotated.html]

HRL Haptic MPC: Class List


HRL Haptic MPC

A haptic model predictive controller

- Main Page
- Packages
- Classes

- Class List
- Class Index
- Class Members

Class List

Here are the classes, structs, unions and interfaces with brief descriptions:

|  |  |
| --- | --- |
| hrl\_haptic\_mpc.haptic\_mpc.HapticMPC | Haptic Model Predictive Controller class |
| hrl\_haptic\_mpc.hrl\_arm.HRLArm |  |
| hrl\_haptic\_mpc.hrl\_arm.HRLArmKinematics |  |
| hrl\_haptic\_mpc.haptic\_mpc.MPCData | Container class for the controller parameters |
| hrl\_haptic\_mpc.mpc\_teleop\_rviz.MPCTeleopInteractiveMarkers | RViz teleop interface to the controller |
| hrl\_haptic\_mpc.multiarray\_to\_matrix.MultiArrayConverter | Helper class to convert numpy matrix lists to and from a Float64MultiArray message object |
| hrl\_haptic\_mpc.gen\_sim\_arms.ODESimArm | Simulation Arm client |
| hrl\_haptic\_mpc.pr2\_skin\_client.PR2\_SkinClient | PR2 specific implementation of the TaxelArrayClient class |
| hrl\_haptic\_mpc.robot\_haptic\_state\_node.RobotHapticStateServer | Publisher: publishes all relevant haptic state information on a common interface independent of robot type |
| hrl\_haptic\_mpc.gen\_sim\_arms.RobotSimulatorKDL | KDL for kinematics etc |
| hrl\_haptic\_mpc.sim\_skin\_client.SimSkinClient | Skin client for the Simulation 3DOF arm |
| hrl\_haptic\_mpc.skin\_client.TaxelArrayClient | Provides an interface to process TaxelArrays published on multiple topics, including trimming the data and transforming the reference frame |
| hrl\_haptic\_mpc.urdf\_arm\_darpa\_m3.URDFArm |  |
| hrl\_haptic\_mpc.waypoint\_generator.WaypointGenerator | Takes either a goal pose or a trajectory and passes local goals to the MPC controller to try to make the controller follow it |


All Classes Namespaces Functions Variables

---

Generated on Thu Jan 17 2013 16:21:14 for HRL Haptic MPC by  

 1.7.6.1
