# Supplementary material for: In-home and remote use of robotic body surrogates by people with profound motor deficits
Source: PLoS One. 2019 Mar 15;14(3):e0212904. doi: 10.1371/journal.pone.0212904 (PMC6420017; doi:10.1371/journal.pone.0212904)
Supplement: S1 Source Code — This includes all custom code used in the described system and related evaluations. (ZIP) [file pone.0212904.s009.zip › robotic_body_surrogates_source_code/hrl-haptic-manip-pkgs.zip!/hrl-haptic-manip-pkgs/hrl_haptic_mpc/docs/html/classes.html]

HRL Haptic MPC: Class Index


HRL Haptic MPC

A haptic model predictive controller

- Main Page
- Packages
- Classes

- Class List
- Class Index
- Class Members

Class Index

H | M | O | P | R | S | T | U | W

|  |  |  |  |  |
| --- | --- | --- | --- | --- |
| H | MPCTeleopInteractiveMarkers (hrl\_haptic\_mpc.mpc\_teleop\_rviz) | R | T |  |
|  | MultiArrayConverter (hrl\_haptic\_mpc.multiarray\_to\_matrix) |  |  |  |
| HapticMPC (hrl\_haptic\_mpc.haptic\_mpc) | O | RobotHapticStateServer (hrl\_haptic\_mpc.robot\_haptic\_state\_node) | TaxelArrayClient (hrl\_haptic\_mpc.skin\_client) |  |
| HRLArm (hrl\_haptic\_mpc.hrl\_arm) |  | RobotSimulatorKDL (hrl\_haptic\_mpc.gen\_sim\_arms) | U |  |
| HRLArmKinematics (hrl\_haptic\_mpc.hrl\_arm) | ODESimArm (hrl\_haptic\_mpc.gen\_sim\_arms) | S |  |  |
| M | P |  | URDFArm (hrl\_haptic\_mpc.urdf\_arm\_darpa\_m3) |  |
|  |  | SimSkinClient (hrl\_haptic\_mpc.sim\_skin\_client) | W |  |
| MPCData (hrl\_haptic\_mpc.haptic\_mpc) | PR2\_SkinClient (hrl\_haptic\_mpc.pr2\_skin\_client) |  |  |  |
|  |  |  | WaypointGenerator (hrl\_haptic\_mpc.waypoint\_generator) |  |
|  |  |  |  |  |

H | M | O | P | R | S | T | U | W


All Classes Namespaces Functions Variables

---

Generated on Thu Jan 17 2013 16:21:14 for HRL Haptic MPC by  

 1.7.6.1
