# Supplementary material for: In-home and remote use of robotic body surrogates by people with profound motor deficits
Source: PLoS One. 2019 Mar 15;14(3):e0212904. doi: 10.1371/journal.pone.0212904 (PMC6420017; doi:10.1371/journal.pone.0212904)
Supplement: S1 Source Code — This includes all custom code used in the described system and related evaluations. (ZIP) [file pone.0212904.s009.zip › robotic_body_surrogates_source_code/hrl-haptic-manip-pkgs.zip!/hrl-haptic-manip-pkgs/hrl_haptic_mpc/docs/html/classhrl__haptic__mpc_1_1gen__sim__arms_1_1_o_d_e_sim_arm-members.html]

HRL Haptic MPC: Member List


HRL Haptic MPC

A haptic model predictive controller

- Main Page
- Packages
- Classes

- Class List
- Class Index
- Class Members

- hrl\_haptic\_mpc
- **gen\_sim\_arms**
- ODESimArm

hrl\_haptic\_mpc.gen\_sim\_arms.ODESimArm Member List

This is the complete list of members for hrl\_haptic\_mpc.gen\_sim\_arms.ODESimArm, including all inherited members.

|  |  |  |
| --- | --- | --- |
| **\_\_init\_\_** (defined in hrl\_haptic\_mpc.gen\_sim\_arms.ODESimArm) | hrl\_haptic\_mpc.gen\_sim\_arms.ODESimArm |  |
| **cep\_marker\_pub** (defined in hrl\_haptic\_mpc.gen\_sim\_arms.ODESimArm) | hrl\_haptic\_mpc.gen\_sim\_arms.ODESimArm |  |
| **ep** (defined in hrl\_haptic\_mpc.gen\_sim\_arms.ODESimArm) | hrl\_haptic\_mpc.gen\_sim\_arms.ODESimArm |  |
| **get\_joint\_velocities** (defined in hrl\_haptic\_mpc.gen\_sim\_arms.ODESimArm) | hrl\_haptic\_mpc.gen\_sim\_arms.ODESimArm |  |
| **impedance\_params\_cb** (defined in hrl\_haptic\_mpc.gen\_sim\_arms.ODESimArm) | hrl\_haptic\_mpc.gen\_sim\_arms.ODESimArm |  |
| **impedance\_pub** (defined in hrl\_haptic\_mpc.gen\_sim\_arms.ODESimArm) | hrl\_haptic\_mpc.gen\_sim\_arms.ODESimArm |  |
| **jep\_cb** (defined in hrl\_haptic\_mpc.gen\_sim\_arms.ODESimArm) | hrl\_haptic\_mpc.gen\_sim\_arms.ODESimArm |  |
| **jep\_pub** (defined in hrl\_haptic\_mpc.gen\_sim\_arms.ODESimArm) | hrl\_haptic\_mpc.gen\_sim\_arms.ODESimArm |  |
| **joint\_names\_list** (defined in hrl\_haptic\_mpc.gen\_sim\_arms.ODESimArm) | hrl\_haptic\_mpc.gen\_sim\_arms.ODESimArm |  |
| **joint\_rates\_cb** (defined in hrl\_haptic\_mpc.gen\_sim\_arms.ODESimArm) | hrl\_haptic\_mpc.gen\_sim\_arms.ODESimArm |  |
| **joint\_states\_cb** (defined in hrl\_haptic\_mpc.gen\_sim\_arms.ODESimArm) | hrl\_haptic\_mpc.gen\_sim\_arms.ODESimArm |  |
| **kd** (defined in hrl\_haptic\_mpc.gen\_sim\_arms.ODESimArm) | hrl\_haptic\_mpc.gen\_sim\_arms.ODESimArm |  |
| **kp** (defined in hrl\_haptic\_mpc.gen\_sim\_arms.ODESimArm) | hrl\_haptic\_mpc.gen\_sim\_arms.ODESimArm |  |
| **publish\_rviz\_markers** (defined in hrl\_haptic\_mpc.gen\_sim\_arms.ODESimArm) | hrl\_haptic\_mpc.gen\_sim\_arms.ODESimArm |  |
| **q** (defined in hrl\_haptic\_mpc.gen\_sim\_arms.ODESimArm) | hrl\_haptic\_mpc.gen\_sim\_arms.ODESimArm |  |
| **qdot** (defined in hrl\_haptic\_mpc.gen\_sim\_arms.ODESimArm) | hrl\_haptic\_mpc.gen\_sim\_arms.ODESimArm |  |
| **set\_delta\_ep\_ros** (defined in hrl\_haptic\_mpc.gen\_sim\_arms.ODESimArm) | hrl\_haptic\_mpc.gen\_sim\_arms.ODESimArm |  |
| **set\_ep** (defined in hrl\_haptic\_mpc.gen\_sim\_arms.ODESimArm) | hrl\_haptic\_mpc.gen\_sim\_arms.ODESimArm |  |
| **set\_ep\_ros** (defined in hrl\_haptic\_mpc.gen\_sim\_arms.ODESimArm) | hrl\_haptic\_mpc.gen\_sim\_arms.ODESimArm |  |
| **set\_joint\_impedance** (defined in hrl\_haptic\_mpc.gen\_sim\_arms.ODESimArm) | hrl\_haptic\_mpc.gen\_sim\_arms.ODESimArm |  |


All Classes Namespaces Functions Variables

---

Generated on Thu Jan 17 2013 16:21:14 for HRL Haptic MPC by  

 1.7.6.1
