# Supplementary material for: In-home and remote use of robotic body surrogates by people with profound motor deficits
Source: PLoS One. 2019 Mar 15;14(3):e0212904. doi: 10.1371/journal.pone.0212904 (PMC6420017; doi:10.1371/journal.pone.0212904)
Supplement: S1 Source Code — This includes all custom code used in the described system and related evaluations. (ZIP) [file pone.0212904.s009.zip › robotic_body_surrogates_source_code/hrl-haptic-manip-pkgs.zip!/hrl-haptic-manip-pkgs/hrl_haptic_mpc/docs/html/classhrl__haptic__mpc_1_1gen__sim__arms_1_1_robot_simulator_k_d_l-members.html]

HRL Haptic MPC: Member List


HRL Haptic MPC

A haptic model predictive controller

- Main Page
- Packages
- Classes

- Class List
- Class Index
- Class Members

- hrl\_haptic\_mpc
- **gen\_sim\_arms**
- RobotSimulatorKDL

hrl\_haptic\_mpc.gen\_sim\_arms.RobotSimulatorKDL Member List

This is the complete list of members for hrl\_haptic\_mpc.gen\_sim\_arms.RobotSimulatorKDL, including all inherited members.

|  |  |  |
| --- | --- | --- |
| **\_\_init\_\_** (defined in hrl\_haptic\_mpc.gen\_sim\_arms.RobotSimulatorKDL) | hrl\_haptic\_mpc.gen\_sim\_arms.RobotSimulatorKDL |  |
| **arm\_type** (defined in hrl\_haptic\_mpc.gen\_sim\_arms.RobotSimulatorKDL) | hrl\_haptic\_mpc.gen\_sim\_arms.RobotSimulatorKDL |  |
| **clamp\_to\_joint\_limits** (defined in hrl\_haptic\_mpc.gen\_sim\_arms.RobotSimulatorKDL) | hrl\_haptic\_mpc.gen\_sim\_arms.RobotSimulatorKDL |  |
| **create\_right\_chain** (defined in hrl\_haptic\_mpc.gen\_sim\_arms.RobotSimulatorKDL) | hrl\_haptic\_mpc.gen\_sim\_arms.RobotSimulatorKDL |  |
| **create\_solvers** (defined in hrl\_haptic\_mpc.gen\_sim\_arms.RobotSimulatorKDL) | hrl\_haptic\_mpc.gen\_sim\_arms.RobotSimulatorKDL |  |
| **d\_robot** (defined in hrl\_haptic\_mpc.gen\_sim\_arms.RobotSimulatorKDL) | hrl\_haptic\_mpc.gen\_sim\_arms.RobotSimulatorKDL |  |
| **FK\_kdl** (defined in hrl\_haptic\_mpc.gen\_sim\_arms.RobotSimulatorKDL) | hrl\_haptic\_mpc.gen\_sim\_arms.RobotSimulatorKDL |  |
| FK\_vanilla | hrl\_haptic\_mpc.gen\_sim\_arms.RobotSimulatorKDL |  |
| **get\_joint\_limits** (defined in hrl\_haptic\_mpc.gen\_sim\_arms.RobotSimulatorKDL) | hrl\_haptic\_mpc.gen\_sim\_arms.RobotSimulatorKDL |  |
| jacobian | hrl\_haptic\_mpc.gen\_sim\_arms.RobotSimulatorKDL |  |
| **kdl\_to\_list** (defined in hrl\_haptic\_mpc.gen\_sim\_arms.RobotSimulatorKDL) | hrl\_haptic\_mpc.gen\_sim\_arms.RobotSimulatorKDL |  |
| **linkage\_offset\_from\_ground** (defined in hrl\_haptic\_mpc.gen\_sim\_arms.RobotSimulatorKDL) | hrl\_haptic\_mpc.gen\_sim\_arms.RobotSimulatorKDL |  |
| **list\_to\_kdl** (defined in hrl\_haptic\_mpc.gen\_sim\_arms.RobotSimulatorKDL) | hrl\_haptic\_mpc.gen\_sim\_arms.RobotSimulatorKDL |  |
| **max\_jtlim\_arr** (defined in hrl\_haptic\_mpc.gen\_sim\_arms.RobotSimulatorKDL) | hrl\_haptic\_mpc.gen\_sim\_arms.RobotSimulatorKDL |  |
| **min\_jtlim\_arr** (defined in hrl\_haptic\_mpc.gen\_sim\_arms.RobotSimulatorKDL) | hrl\_haptic\_mpc.gen\_sim\_arms.RobotSimulatorKDL |  |
| **n\_jts** (defined in hrl\_haptic\_mpc.gen\_sim\_arms.RobotSimulatorKDL) | hrl\_haptic\_mpc.gen\_sim\_arms.RobotSimulatorKDL |  |
| **plot\_arm** (defined in hrl\_haptic\_mpc.gen\_sim\_arms.RobotSimulatorKDL) | hrl\_haptic\_mpc.gen\_sim\_arms.RobotSimulatorKDL |  |
| **right\_chain** (defined in hrl\_haptic\_mpc.gen\_sim\_arms.RobotSimulatorKDL) | hrl\_haptic\_mpc.gen\_sim\_arms.RobotSimulatorKDL |  |
| **right\_fk** (defined in hrl\_haptic\_mpc.gen\_sim\_arms.RobotSimulatorKDL) | hrl\_haptic\_mpc.gen\_sim\_arms.RobotSimulatorKDL |  |
| **right\_ik\_p** (defined in hrl\_haptic\_mpc.gen\_sim\_arms.RobotSimulatorKDL) | hrl\_haptic\_mpc.gen\_sim\_arms.RobotSimulatorKDL |  |
| **right\_ik\_v** (defined in hrl\_haptic\_mpc.gen\_sim\_arms.RobotSimulatorKDL) | hrl\_haptic\_mpc.gen\_sim\_arms.RobotSimulatorKDL |  |
| **right\_jac** (defined in hrl\_haptic\_mpc.gen\_sim\_arms.RobotSimulatorKDL) | hrl\_haptic\_mpc.gen\_sim\_arms.RobotSimulatorKDL |  |
| **right\_tooltip** (defined in hrl\_haptic\_mpc.gen\_sim\_arms.RobotSimulatorKDL) | hrl\_haptic\_mpc.gen\_sim\_arms.RobotSimulatorKDL |  |
| **within\_joint\_limits** (defined in hrl\_haptic\_mpc.gen\_sim\_arms.RobotSimulatorKDL) | hrl\_haptic\_mpc.gen\_sim\_arms.RobotSimulatorKDL |  |


All Classes Namespaces Functions Variables

---

Generated on Thu Jan 17 2013 16:21:14 for HRL Haptic MPC by  

 1.7.6.1
