# Supplementary material for: In-home and remote use of robotic body surrogates by people with profound motor deficits
Source: PLoS One. 2019 Mar 15;14(3):e0212904. doi: 10.1371/journal.pone.0212904 (PMC6420017; doi:10.1371/journal.pone.0212904)
Supplement: S1 Source Code — This includes all custom code used in the described system and related evaluations. (ZIP) [file pone.0212904.s009.zip › robotic_body_surrogates_source_code/hrl-haptic-manip-pkgs.zip!/hrl-haptic-manip-pkgs/hrl_haptic_mpc/docs/html/classhrl__haptic__mpc_1_1gen__sim__arms_1_1_robot_simulator_k_d_l.html]

HRL Haptic MPC: hrl\_haptic\_mpc.gen\_sim\_arms.RobotSimulatorKDL Class Reference


HRL Haptic MPC

A haptic model predictive controller

- Main Page
- Packages
- Classes

- Class List
- Class Index
- Class Members

All Classes Namespaces Functions Variables

- hrl\_haptic\_mpc
- **gen\_sim\_arms**
- RobotSimulatorKDL

Public Member Functions |
Public Attributes

hrl\_haptic\_mpc.gen\_sim\_arms.RobotSimulatorKDL Class Reference

KDL for kinematics etc.
More...

List of all members.

|  |  |
| --- | --- |
| Public Member Functions | |
| def | **\_\_init\_\_** |
| def | **create\_right\_chain** |
| def | **create\_solvers** |
| def | **FK\_kdl** |
| def | FK\_vanilla |
|  | returns point in torso lift link. |
| def | **kdl\_to\_list** |
| def | **list\_to\_kdl** |
| def | jacobian |
|  | compute Jacobian at point pos. |
| def | **clamp\_to\_joint\_limits** |
| def | **within\_joint\_limits** |
| def | **get\_joint\_limits** |
| def | **plot\_arm** |
| Public Attributes | |
|  | **arm\_type** |
|  | **d\_robot** |
|  | **right\_chain** |
|  | **right\_fk** |
|  | **right\_ik\_v** |
|  | **right\_ik\_p** |
|  | **right\_jac** |
|  | **right\_tooltip** |
|  | **min\_jtlim\_arr** |
|  | **max\_jtlim\_arr** |
|  | **linkage\_offset\_from\_ground** |
|  | **n\_jts** |

---

## Detailed Description

KDL for kinematics etc.

---

## Member Function Documentation

|  |  |  |  |
| --- | --- | --- | --- |
| def hrl\_haptic\_mpc.gen\_sim\_arms.RobotSimulatorKDL.FK\_vanilla | ( |  | *self*, |
|  |  |  | *q*, |
|  |  |  | *link\_number* = `None` |
|  | ) |  |  |

returns point in torso lift link.

|  |  |  |  |
| --- | --- | --- | --- |
| def hrl\_haptic\_mpc.gen\_sim\_arms.RobotSimulatorKDL.jacobian | ( |  | *self*, |
|  |  |  | *q*, |
|  |  |  | *pos* = `None` |
|  | ) |  |  |

compute Jacobian at point pos.

p is in the ground coord frame.

---

The documentation for this class was generated from the following file:

- /home/jdh/ros\_sandbox/hrl\_haptic\_manipulation\_in\_clutter/hrl\_haptic\_mpc/src/hrl\_haptic\_mpc/gen\_sim\_arms.py


All Classes Namespaces Functions Variables

---

Generated on Thu Jan 17 2013 16:21:14 for HRL Haptic MPC by  

 1.7.6.1
