# Supplementary material for: In-home and remote use of robotic body surrogates by people with profound motor deficits
Source: PLoS One. 2019 Mar 15;14(3):e0212904. doi: 10.1371/journal.pone.0212904 (PMC6420017; doi:10.1371/journal.pone.0212904)
Supplement: S1 Source Code — This includes all custom code used in the described system and related evaluations. (ZIP) [file pone.0212904.s009.zip › robotic_body_surrogates_source_code/hrl-haptic-manip-pkgs.zip!/hrl-haptic-manip-pkgs/hrl_haptic_mpc/docs/html/classhrl__haptic__mpc_1_1haptic__mpc_1_1_haptic_m_p_c-members.html]

HRL Haptic MPC: Member List


HRL Haptic MPC

A haptic model predictive controller

- Main Page
- Packages
- Classes

- Class List
- Class Index
- Class Members

- hrl\_haptic\_mpc
- **haptic\_mpc**
- HapticMPC

hrl\_haptic\_mpc.haptic\_mpc.HapticMPC Member List

This is the complete list of members for hrl\_haptic\_mpc.haptic\_mpc.HapticMPC, including all inherited members.

|  |  |  |
| --- | --- | --- |
| \_\_init\_\_ | hrl\_haptic\_mpc.haptic\_mpc.HapticMPC |  |
| **allowable\_contact\_force** (defined in hrl\_haptic\_mpc.haptic\_mpc.HapticMPC) | hrl\_haptic\_mpc.haptic\_mpc.HapticMPC |  |
| contactForceTransformationMatrix | hrl\_haptic\_mpc.haptic\_mpc.HapticMPC |  |
| contactStiffnessMatrix | hrl\_haptic\_mpc.haptic\_mpc.HapticMPC |  |
| **currently\_in\_deadzone** (defined in hrl\_haptic\_mpc.haptic\_mpc.HapticMPC) | hrl\_haptic\_mpc.haptic\_mpc.HapticMPC |  |
| **deadzone\_angle** (defined in hrl\_haptic\_mpc.haptic\_mpc.HapticMPC) | hrl\_haptic\_mpc.haptic\_mpc.HapticMPC |  |
| **deadzone\_distance** (defined in hrl\_haptic\_mpc.haptic\_mpc.HapticMPC) | hrl\_haptic\_mpc.haptic\_mpc.HapticMPC |  |
| **delta\_q\_des\_pub** (defined in hrl\_haptic\_mpc.haptic\_mpc.HapticMPC) | hrl\_haptic\_mpc.haptic\_mpc.HapticMPC |  |
| deltaForceBounds | hrl\_haptic\_mpc.haptic\_mpc.HapticMPC |  |
| deltaQpJepGen | hrl\_haptic\_mpc.haptic\_mpc.HapticMPC |  |
| **desired\_joint\_angles** (defined in hrl\_haptic\_mpc.haptic\_mpc.HapticMPC) | hrl\_haptic\_mpc.haptic\_mpc.HapticMPC |  |
| **enable\_mpc\_srv** (defined in hrl\_haptic\_mpc.haptic\_mpc.HapticMPC) | hrl\_haptic\_mpc.haptic\_mpc.HapticMPC |  |
| enableHapticMPC | hrl\_haptic\_mpc.haptic\_mpc.HapticMPC |  |
| **end\_effector\_orient\_quat** (defined in hrl\_haptic\_mpc.haptic\_mpc.HapticMPC) | hrl\_haptic\_mpc.haptic\_mpc.HapticMPC |  |
| **end\_effector\_pos** (defined in hrl\_haptic\_mpc.haptic\_mpc.HapticMPC) | hrl\_haptic\_mpc.haptic\_mpc.HapticMPC |  |
| **estimate\_contact\_stiffness** (defined in hrl\_haptic\_mpc.haptic\_mpc.HapticMPC) | hrl\_haptic\_mpc.haptic\_mpc.HapticMPC |  |
| **frequency** (defined in hrl\_haptic\_mpc.haptic\_mpc.HapticMPC) | hrl\_haptic\_mpc.haptic\_mpc.HapticMPC |  |
| gain\_lock | hrl\_haptic\_mpc.haptic\_mpc.HapticMPC |  |
| getDesiredJointAngles | hrl\_haptic\_mpc.haptic\_mpc.HapticMPC |  |
| getJointAngles | hrl\_haptic\_mpc.haptic\_mpc.HapticMPC |  |
| getJointStiffness | hrl\_haptic\_mpc.haptic\_mpc.HapticMPC |  |
| getMPCData | hrl\_haptic\_mpc.haptic\_mpc.HapticMPC |  |
| getSkinData | hrl\_haptic\_mpc.haptic\_mpc.HapticMPC |  |
| goal\_lock | hrl\_haptic\_mpc.haptic\_mpc.HapticMPC |  |
| **goal\_orient\_quat** (defined in hrl\_haptic\_mpc.haptic\_mpc.HapticMPC) | hrl\_haptic\_mpc.haptic\_mpc.HapticMPC |  |
| **goal\_pos** (defined in hrl\_haptic\_mpc.haptic\_mpc.HapticMPC) | hrl\_haptic\_mpc.haptic\_mpc.HapticMPC |  |
| **goal\_sub** (defined in hrl\_haptic\_mpc.haptic\_mpc.HapticMPC) | hrl\_haptic\_mpc.haptic\_mpc.HapticMPC |  |
| **goal\_velocity\_for\_hand** (defined in hrl\_haptic\_mpc.haptic\_mpc.HapticMPC) | hrl\_haptic\_mpc.haptic\_mpc.HapticMPC |  |
| goalCallback | hrl\_haptic\_mpc.haptic\_mpc.HapticMPC |  |
| goalMotionForHand | hrl\_haptic\_mpc.haptic\_mpc.HapticMPC |  |
| goalOrientationInQuat | hrl\_haptic\_mpc.haptic\_mpc.HapticMPC |  |
| initComms | hrl\_haptic\_mpc.haptic\_mpc.HapticMPC |  |
| initControlParametersFromServer | hrl\_haptic\_mpc.haptic\_mpc.HapticMPC |  |
| initMPCData | hrl\_haptic\_mpc.haptic\_mpc.HapticMPC |  |
| initRobot | hrl\_haptic\_mpc.haptic\_mpc.HapticMPC |  |
| **Jc** (defined in hrl\_haptic\_mpc.haptic\_mpc.HapticMPC) | hrl\_haptic\_mpc.haptic\_mpc.HapticMPC |  |
| **Je** (defined in hrl\_haptic\_mpc.haptic\_mpc.HapticMPC) | hrl\_haptic\_mpc.haptic\_mpc.HapticMPC |  |
| **jerk\_opt\_weight** (defined in hrl\_haptic\_mpc.haptic\_mpc.HapticMPC) | hrl\_haptic\_mpc.haptic\_mpc.HapticMPC |  |
| **joint\_angles** (defined in hrl\_haptic\_mpc.haptic\_mpc.HapticMPC) | hrl\_haptic\_mpc.haptic\_mpc.HapticMPC |  |
| **joint\_damping** (defined in hrl\_haptic\_mpc.haptic\_mpc.HapticMPC) | hrl\_haptic\_mpc.haptic\_mpc.HapticMPC |  |
| **joint\_limits\_max** (defined in hrl\_haptic\_mpc.haptic\_mpc.HapticMPC) | hrl\_haptic\_mpc.haptic\_mpc.HapticMPC |  |
| **joint\_limits\_min** (defined in hrl\_haptic\_mpc.haptic\_mpc.HapticMPC) | hrl\_haptic\_mpc.haptic\_mpc.HapticMPC |  |
| joint\_names | hrl\_haptic\_mpc.haptic\_mpc.HapticMPC |  |
| **joint\_stiffness** (defined in hrl\_haptic\_mpc.haptic\_mpc.HapticMPC) | hrl\_haptic\_mpc.haptic\_mpc.HapticMPC |  |
| **joint\_velocities** (defined in hrl\_haptic\_mpc.haptic\_mpc.HapticMPC) | hrl\_haptic\_mpc.haptic\_mpc.HapticMPC |  |
| last\_msg\_time | hrl\_haptic\_mpc.haptic\_mpc.HapticMPC |  |
| **ma\_to\_m** (defined in hrl\_haptic\_mpc.haptic\_mpc.HapticMPC) | hrl\_haptic\_mpc.haptic\_mpc.HapticMPC |  |
| **max\_delta\_force\_mag** (defined in hrl\_haptic\_mpc.haptic\_mpc.HapticMPC) | hrl\_haptic\_mpc.haptic\_mpc.HapticMPC |  |
| monitor\_lock | hrl\_haptic\_mpc.haptic\_mpc.HapticMPC |  |
| mpc\_data | hrl\_haptic\_mpc.haptic\_mpc.HapticMPC |  |
| **mpc\_enabled** (defined in hrl\_haptic\_mpc.haptic\_mpc.HapticMPC) | hrl\_haptic\_mpc.haptic\_mpc.HapticMPC |  |
| mpc\_error | hrl\_haptic\_mpc.haptic\_mpc.HapticMPC |  |
| **mpc\_monitor\_sub** (defined in hrl\_haptic\_mpc.haptic\_mpc.HapticMPC) | hrl\_haptic\_mpc.haptic\_mpc.HapticMPC |  |
| **mpc\_state** (defined in hrl\_haptic\_mpc.haptic\_mpc.HapticMPC) | hrl\_haptic\_mpc.haptic\_mpc.HapticMPC |  |
| **mpc\_weights\_pub** (defined in hrl\_haptic\_mpc.haptic\_mpc.HapticMPC) | hrl\_haptic\_mpc.haptic\_mpc.HapticMPC |  |
| **mpc\_weights\_sub** (defined in hrl\_haptic\_mpc.haptic\_mpc.HapticMPC) | hrl\_haptic\_mpc.haptic\_mpc.HapticMPC |  |
| mpcMonitorCallback | hrl\_haptic\_mpc.haptic\_mpc.HapticMPC |  |
| msg | hrl\_haptic\_mpc.haptic\_mpc.HapticMPC |  |
| **opt** (defined in hrl\_haptic\_mpc.haptic\_mpc.HapticMPC) | hrl\_haptic\_mpc.haptic\_mpc.HapticMPC |  |
| **orient\_weight** (defined in hrl\_haptic\_mpc.haptic\_mpc.HapticMPC) | hrl\_haptic\_mpc.haptic\_mpc.HapticMPC |  |
| **pos\_weight** (defined in hrl\_haptic\_mpc.haptic\_mpc.HapticMPC) | hrl\_haptic\_mpc.haptic\_mpc.HapticMPC |  |
| publishDeltaControlValues | hrl\_haptic\_mpc.haptic\_mpc.HapticMPC |  |
| publishDesiredJointAngles | hrl\_haptic\_mpc.haptic\_mpc.HapticMPC |  |
| **q\_des\_pub** (defined in hrl\_haptic\_mpc.haptic\_mpc.HapticMPC) | hrl\_haptic\_mpc.haptic\_mpc.HapticMPC |  |
| **robot\_state\_sub** (defined in hrl\_haptic\_mpc.haptic\_mpc.HapticMPC) | hrl\_haptic\_mpc.haptic\_mpc.HapticMPC |  |
| robotStateCallback | hrl\_haptic\_mpc.haptic\_mpc.HapticMPC |  |
| **skin\_data** (defined in hrl\_haptic\_mpc.haptic\_mpc.HapticMPC) | hrl\_haptic\_mpc.haptic\_mpc.HapticMPC |  |
| start | hrl\_haptic\_mpc.haptic\_mpc.HapticMPC |  |
| **state\_lock** (defined in hrl\_haptic\_mpc.haptic\_mpc.HapticMPC) | hrl\_haptic\_mpc.haptic\_mpc.HapticMPC |  |
| **static\_contact\_stiffness\_estimate** (defined in hrl\_haptic\_mpc.haptic\_mpc.HapticMPC) | hrl\_haptic\_mpc.haptic\_mpc.HapticMPC |  |
| **stopping\_force** (defined in hrl\_haptic\_mpc.haptic\_mpc.HapticMPC) | hrl\_haptic\_mpc.haptic\_mpc.HapticMPC |  |
| **time\_step** (defined in hrl\_haptic\_mpc.haptic\_mpc.HapticMPC) | hrl\_haptic\_mpc.haptic\_mpc.HapticMPC |  |
| **timeout** (defined in hrl\_haptic\_mpc.haptic\_mpc.HapticMPC) | hrl\_haptic\_mpc.haptic\_mpc.HapticMPC |  |
| updateController | hrl\_haptic\_mpc.haptic\_mpc.HapticMPC |  |
| updateWeightsCallback | hrl\_haptic\_mpc.haptic\_mpc.HapticMPC |  |
| **waiting\_for\_no\_errors** (defined in hrl\_haptic\_mpc.haptic\_mpc.HapticMPC) | hrl\_haptic\_mpc.haptic\_mpc.HapticMPC |  |
| **waiting\_to\_resume** (defined in hrl\_haptic\_mpc.haptic\_mpc.HapticMPC) | hrl\_haptic\_mpc.haptic\_mpc.HapticMPC |  |


All Classes Namespaces Functions Variables

---

Generated on Thu Jan 17 2013 16:21:14 for HRL Haptic MPC by  

 1.7.6.1
