# Supplementary material for: In-home and remote use of robotic body surrogates by people with profound motor deficits
Source: PLoS One. 2019 Mar 15;14(3):e0212904. doi: 10.1371/journal.pone.0212904 (PMC6420017; doi:10.1371/journal.pone.0212904)
Supplement: S1 Source Code — This includes all custom code used in the described system and related evaluations. (ZIP) [file pone.0212904.s009.zip › robotic_body_surrogates_source_code/hrl-haptic-manip-pkgs.zip!/hrl-haptic-manip-pkgs/hrl_haptic_mpc/docs/html/classhrl__haptic__mpc_1_1haptic__mpc_1_1_haptic_m_p_c.html]

HRL Haptic MPC: hrl\_haptic\_mpc.haptic\_mpc.HapticMPC Class Reference


HRL Haptic MPC

A haptic model predictive controller

- Main Page
- Packages
- Classes

- Class List
- Class Index
- Class Members

All Classes Namespaces Functions Variables

- hrl\_haptic\_mpc
- **haptic\_mpc**
- HapticMPC

Public Member Functions |
Public Attributes

hrl\_haptic\_mpc.haptic\_mpc.HapticMPC Class Reference

Haptic Model Predictive Controller class.
More...

List of all members.

|  |  |
| --- | --- |
| Public Member Functions | |
| def | \_\_init\_\_ |
|  | Constructor. |
| def | initControlParametersFromServer |
|  | Read parameters from the ROS parameter server and store them. |
| def | initRobot |
|  | Initialise the robot joint limits. |
| def | getSkinData |
|  | getSkinData accessor function. |
| def | getJointAngles |
|  | getJointAngles accessor function. |
| def | getDesiredJointAngles |
|  | getDesiredJointAngles accessor function. |
| def | getJointStiffness |
|  | getJointStiffness accessor function. |
| def | getMPCData |
|  | getMPCData accessor function. |
| def | initMPCData |
|  | Builds the MPC data structure from the individual parameters This is just a convenience data structure to amalgamate the data. |
| def | updateWeightsCallback |
|  | Update the position/orientation weights used by the controller. |
| def | goalCallback |
|  | Store the current trajectory goal. |
| def | mpcMonitorCallback |
|  | Store the state information from the monitor node. |
| def | robotStateCallback |
|  | Store the robot haptic state. |
| def | goalOrientationInQuat |
|  | Interpolate a step towards the given goal orientation. |
| def | goalMotionForHand |
|  | Interpolate a step towards the given goal position. |
| def | deltaQpJepGen |
|  | Main control calculation. |
| def | contactStiffnessMatrix |
|  | Computes the contact stiffness matrix, K\_ci. |
| def | contactForceTransformationMatrix |
|  | Calculates the force transformation matrix from a list of force normals. |
| def | deltaForceBounds |
|  | Compute bounds for delta\_f. |
| def | publishDeltaControlValues |
|  | Publishes a list of Deltas for the desired joint positions. |
| def | publishDesiredJointAngles |
|  | Publish a desired joint position list directly (rather than deltas) |
| def | updateController |
|  | Main control function. |
| def | initComms |
|  | Initialise the ROS communications - init node, subscribe to the robot state and goal pos, publish JEPs. |
| def | enableHapticMPC |
|  | Enable Haptic MPC service handler (default is enabled). |
| def | start |
|  | Start the control loop once the controller is initialised. |
| Public Attributes | |
|  | **opt** |
|  | **state\_lock** |
|  | goal\_lock |
|  | Haptic state lock. |
|  | monitor\_lock |
|  | Goal state lock. |
|  | gain\_lock |
|  | Monitor state lock. |
|  | mpc\_data |
|  | Controller gain state lock. |
|  | msg |
|  | MPCData data structure. |
|  | last\_msg\_time |
|  | Haptic state message. |
|  | **timeout** |
|  | **waiting\_to\_resume** |
|  | **waiting\_for\_no\_errors** |
|  | **mpc\_state** |
|  | mpc\_error |
|  | Current MPC state. |
|  | joint\_names |
|  | Current MPC errors. |
|  | **joint\_angles** |
|  | **desired\_joint\_angles** |
|  | **joint\_velocities** |
|  | **joint\_stiffness** |
|  | **joint\_damping** |
|  | **end\_effector\_pos** |
|  | **end\_effector\_orient\_quat** |
|  | **skin\_data** |
|  | **Jc** |
|  | **Je** |
|  | **time\_step** |
|  | **goal\_pos** |
|  | **goal\_orient\_quat** |
|  | **orient\_weight** |
|  | **pos\_weight** |
|  | **deadzone\_distance** |
|  | **deadzone\_angle** |
|  | **currently\_in\_deadzone** |
|  | **mpc\_enabled** |
|  | **ma\_to\_m** |
|  | **allowable\_contact\_force** |
|  | **max\_delta\_force\_mag** |
|  | **stopping\_force** |
|  | **goal\_velocity\_for\_hand** |
|  | **static\_contact\_stiffness\_estimate** |
|  | **estimate\_contact\_stiffness** |
|  | **jerk\_opt\_weight** |
|  | **frequency** |
|  | **joint\_limits\_max** |
|  | **joint\_limits\_min** |
|  | **robot\_state\_sub** |
|  | **goal\_sub** |
|  | **delta\_q\_des\_pub** |
|  | **q\_des\_pub** |
|  | **mpc\_monitor\_sub** |
|  | **mpc\_weights\_sub** |
|  | **mpc\_weights\_pub** |
|  | **enable\_mpc\_srv** |

---

## Detailed Description

Haptic Model Predictive Controller class.

---

## Constructor & Destructor Documentation

|  |  |  |  |
| --- | --- | --- | --- |
| def hrl\_haptic\_mpc.haptic\_mpc.HapticMPC.\_\_init\_\_ | ( |  | *self*, |
|  |  |  | *opt*, |
|  |  |  | *node\_name* = `"haptic_mpc"` |
|  | ) |  |  |

Constructor.

**Parameters:**
:   |  |  |
    | --- | --- |
    | opt | optparse options. Should be created using the helper utility script for reliability. |
    | node\_name | Name used for the ROS node. |

---

## Member Function Documentation

|  |  |  |  |
| --- | --- | --- | --- |
| def hrl\_haptic\_mpc.haptic\_mpc.HapticMPC.contactForceTransformationMatrix | ( |  | *self*, |
|  |  |  | *n\_l* |
|  | ) |  |  |

Calculates the force transformation matrix from a list of force normals.

**Parameters:**
:   |  |  |
    | --- | --- |
    | n\_l | List of force normals |

**Returns:**
:   List of transformation matrices (rotations)

|  |  |  |  |
| --- | --- | --- | --- |
| def hrl\_haptic\_mpc.haptic\_mpc.HapticMPC.contactStiffnessMatrix | ( |  | *self*, |
|  |  |  | *n\_l*, |
|  |  |  | *k\_default*, |
|  |  |  | *k\_est\_min* = `100.0`, |
|  |  |  | *k\_est\_max* = `100000.0` |
|  | ) |  |  |

Computes the contact stiffness matrix, K\_ci.

**Parameters:**
:   |  |  |
    | --- | --- |
    | n\_l | List of normal forces #param k\_default Default stiffness, N/m. Default is typically 200.0-1000.0 |

|  |  |  |  |
| --- | --- | --- | --- |
| def hrl\_haptic\_mpc.haptic\_mpc.HapticMPC.deltaForceBounds | ( |  | *self*, |
|  |  |  | *f\_n*, |
|  |  |  | *max\_pushing\_force*, |
|  |  |  | *max\_pulling\_force*, |
|  |  |  | *max\_pushing\_force\_increase*, |
|  |  |  | *max\_pushing\_force\_decrease*, |
|  |  |  | *min\_decrease\_when\_over\_max\_force*, |
|  |  |  | *max\_decrease\_when\_over\_max\_force* |
|  | ) |  |  |

Compute bounds for delta\_f.

**Return values:**
:   |  |  |
    | --- | --- |
    | delta\_f\_min | Minimum bound |
    | delta\_f\_max | Maximum bound |

|  |  |  |  |
| --- | --- | --- | --- |
| def hrl\_haptic\_mpc.haptic\_mpc.HapticMPC.deltaQpJepGen | ( |  | *self*, |
|  |  |  | *mpc\_dat* |
|  | ) |  |  |

Main control calculation.

**Parameters:**
:   |  |  |
    | --- | --- |
    | mpc\_dat | An MPCData class, populated with relevant control parameters and current data |

**Returns:**
:   A list of joint equilibrium point deltas, ie, a desired increment to the current joint position(s).

|  |  |  |  |  |  |
| --- | --- | --- | --- | --- | --- |
| def hrl\_haptic\_mpc.haptic\_mpc.HapticMPC.enableHapticMPC | ( |  | *req* | ) |  |

Enable Haptic MPC service handler (default is enabled).

|  |  |  |  |  |  |
| --- | --- | --- | --- | --- | --- |
| def hrl\_haptic\_mpc.haptic\_mpc.HapticMPC.getDesiredJointAngles | ( |  | *self* | ) |  |

getDesiredJointAngles accessor function.

**Returns:**
:   A copy of the desired joint angles list, ie, the current arm controller setpoint.

|  |  |  |  |  |  |
| --- | --- | --- | --- | --- | --- |
| def hrl\_haptic\_mpc.haptic\_mpc.HapticMPC.getJointAngles | ( |  | *self* | ) |  |

getJointAngles accessor function.

**Returns:**
:   A copy of the buffered joint angles list.

|  |  |  |  |  |  |
| --- | --- | --- | --- | --- | --- |
| def hrl\_haptic\_mpc.haptic\_mpc.HapticMPC.getJointStiffness | ( |  | *self* | ) |  |

getJointStiffness accessor function.

**Returns:**
:   A copy of the joint stiffness parameters used by the controller.

|  |  |  |  |  |  |
| --- | --- | --- | --- | --- | --- |
| def hrl\_haptic\_mpc.haptic\_mpc.HapticMPC.getMPCData | ( |  | *self* | ) |  |

getMPCData accessor function.

Returns a copy of the control data structure.

|  |  |  |  |  |  |
| --- | --- | --- | --- | --- | --- |
| def hrl\_haptic\_mpc.haptic\_mpc.HapticMPC.getSkinData | ( |  | *self* | ) |  |

getSkinData accessor function.

**Returns:**
:   A copy of the skin\_data dictionary, containing the latest taxel data.

|  |  |  |  |
| --- | --- | --- | --- |
| def hrl\_haptic\_mpc.haptic\_mpc.HapticMPC.goalCallback | ( |  | *self*, |
|  |  |  | *msg* |
|  | ) |  |  |

Store the current trajectory goal.

The controller will always attempt a linear path to this.

**Parameters:**
:   |  |  |
    | --- | --- |
    | msg | A PoseStamped message |

|  |  |  |  |
| --- | --- | --- | --- |
| def hrl\_haptic\_mpc.haptic\_mpc.HapticMPC.goalMotionForHand | ( |  | *self*, |
|  |  |  | *x\_h*, |
|  |  |  | *x\_g*, |
|  |  |  | *dist\_g* |
|  | ) |  |  |

Interpolate a step towards the given goal position.

**Returns:**
:   A goal delta in position as a numpy matrix.

**Parameters:**
:   |  |  |
    | --- | --- |
    | x\_h | The current hand position as a numpy matrix: [x,y,z] |
    | x\_g | The current goal position as a numpy matrix: [x,y,z] |

|  |  |  |  |
| --- | --- | --- | --- |
| def hrl\_haptic\_mpc.haptic\_mpc.HapticMPC.goalOrientationInQuat | ( |  | *self*, |
|  |  |  | *q\_h\_orient*, |
|  |  |  | *q\_g\_orient*, |
|  |  |  | *max\_ang\_step* |
|  | ) |  |  |

Interpolate a step towards the given goal orientation.

**Parameters:**
:   |  |  |
    | --- | --- |
    | q\_h\_orient | The current hand orientation as a quaternion in list form: [x,y,z,w] |
    | q\_g\_orient | The current goal orientation as a quaternion in list form: [x,y,z,w] |

**Returns:**
:   A desired change in orientation as a delta: TODO: clean this up so it doesn't use the "step" multiplier

|  |  |  |  |  |  |
| --- | --- | --- | --- | --- | --- |
| def hrl\_haptic\_mpc.haptic\_mpc.HapticMPC.initControlParametersFromServer | ( |  | *self* | ) |  |

Read parameters from the ROS parameter server and store them.

|  |  |  |  |  |  |
| --- | --- | --- | --- | --- | --- |
| def hrl\_haptic\_mpc.haptic\_mpc.HapticMPC.initMPCData | ( |  | *self* | ) |  |

Builds the MPC data structure from the individual parameters This is just a convenience data structure to amalgamate the data.

|  |  |  |  |  |  |
| --- | --- | --- | --- | --- | --- |
| def hrl\_haptic\_mpc.haptic\_mpc.HapticMPC.initRobot | ( |  | *self* | ) |  |

Initialise the robot joint limits.

This relies on something (usually the haptic state node) pushing the appropriate joint limits to the param server before execution. NB: All joint limits are specified in degrees and converted to radians here (easier to understand)

|  |  |  |  |
| --- | --- | --- | --- |
| def hrl\_haptic\_mpc.haptic\_mpc.HapticMPC.mpcMonitorCallback | ( |  | *self*, |
|  |  |  | *msg* |
|  | ) |  |  |

Store the state information from the monitor node.

Allows the control to be somewhat stateful. The state and error fields are lists of strings indicating some state.

**Parameters:**
:   |  |  |
    | --- | --- |
    | msg | HapticMpcState message object |

|  |  |  |  |
| --- | --- | --- | --- |
| def hrl\_haptic\_mpc.haptic\_mpc.HapticMPC.publishDeltaControlValues | ( |  | *self*, |
|  |  |  | *ctrl\_data* |
|  | ) |  |  |

Publishes a list of Deltas for the desired joint positions.

**Parameters:**
:   |  |  |
    | --- | --- |
    | ctrl\_data | List of desired joint position deltas to be published |

|  |  |  |  |
| --- | --- | --- | --- |
| def hrl\_haptic\_mpc.haptic\_mpc.HapticMPC.publishDesiredJointAngles | ( |  | *self*, |
|  |  |  | *ctrl\_data* |
|  | ) |  |  |

Publish a desired joint position list directly (rather than deltas)

**Parameters:**
:   |  |  |
    | --- | --- |
    | ctrl\_data | List of desired joint positions to be published (not deltas). |

|  |  |  |  |
| --- | --- | --- | --- |
| def hrl\_haptic\_mpc.haptic\_mpc.HapticMPC.robotStateCallback | ( |  | *self*, |
|  |  |  | *msg* |
|  | ) |  |  |

Store the robot haptic state.

**Parameters:**
:   |  |  |
    | --- | --- |
    | msg | RobotHapticState message object |

|  |  |  |  |  |  |
| --- | --- | --- | --- | --- | --- |
| def hrl\_haptic\_mpc.haptic\_mpc.HapticMPC.start | ( |  | *self* | ) |  |

Start the control loop once the controller is initialised.

|  |  |  |  |  |  |
| --- | --- | --- | --- | --- | --- |
| def hrl\_haptic\_mpc.haptic\_mpc.HapticMPC.updateController | ( |  | *self* | ) |  |

Main control function.

Builds the control data structure and passes it to the control calculation routine

|  |  |  |  |
| --- | --- | --- | --- |
| def hrl\_haptic\_mpc.haptic\_mpc.HapticMPC.updateWeightsCallback | ( |  | *self*, |
|  |  |  | *msg* |
|  | ) |  |  |

Update the position/orientation weights used by the controller.

**Parameters:**
:   |  |  |
    | --- | --- |
    | msg | HapticMpcWeights message object |

---

## Member Data Documentation

hrl\_haptic\_mpc::haptic\_mpc.HapticMPC::joint\_names

Current MPC errors.

Stored as a list of strings

hrl\_haptic\_mpc::haptic\_mpc.HapticMPC::mpc\_error

Current MPC state.

Stored as a list of strings

hrl\_haptic\_mpc::haptic\_mpc.HapticMPC::msg

MPCData data structure.

---

The documentation for this class was generated from the following file:

- /home/jdh/ros\_sandbox/hrl\_haptic\_manipulation\_in\_clutter/hrl\_haptic\_mpc/src/hrl\_haptic\_mpc/haptic\_mpc.py


All Classes Namespaces Functions Variables

---

Generated on Thu Jan 17 2013 16:21:14 for HRL Haptic MPC by  

 1.7.6.1
