# Supplementary material for: In-home and remote use of robotic body surrogates by people with profound motor deficits
Source: PLoS One. 2019 Mar 15;14(3):e0212904. doi: 10.1371/journal.pone.0212904 (PMC6420017; doi:10.1371/journal.pone.0212904)
Supplement: S1 Source Code — This includes all custom code used in the described system and related evaluations. (ZIP) [file pone.0212904.s009.zip › robotic_body_surrogates_source_code/hrl-haptic-manip-pkgs.zip!/hrl-haptic-manip-pkgs/hrl_haptic_mpc/docs/html/classhrl__haptic__mpc_1_1haptic__mpc_1_1_m_p_c_data-members.html]

HRL Haptic MPC: Member List


HRL Haptic MPC

A haptic model predictive controller

- Main Page
- Packages
- Classes

- Class List
- Class Index
- Class Members

- hrl\_haptic\_mpc
- **haptic\_mpc**
- MPCData

hrl\_haptic\_mpc.haptic\_mpc.MPCData Member List

This is the complete list of members for hrl\_haptic\_mpc.haptic\_mpc.MPCData, including all inherited members.

|  |  |  |
| --- | --- | --- |
| **\_\_init\_\_** (defined in hrl\_haptic\_mpc.haptic\_mpc.MPCData) | hrl\_haptic\_mpc.haptic\_mpc.MPCData |  |
| \_\_str\_\_ | hrl\_haptic\_mpc.haptic\_mpc.MPCData |  |
| **control\_point\_joint\_num** (defined in hrl\_haptic\_mpc.haptic\_mpc.MPCData) | hrl\_haptic\_mpc.haptic\_mpc.MPCData |  |
| **delta\_f\_max** (defined in hrl\_haptic\_mpc.haptic\_mpc.MPCData) | hrl\_haptic\_mpc.haptic\_mpc.MPCData |  |
| **delta\_f\_min** (defined in hrl\_haptic\_mpc.haptic\_mpc.MPCData) | hrl\_haptic\_mpc.haptic\_mpc.MPCData |  |
| **dist\_g** (defined in hrl\_haptic\_mpc.haptic\_mpc.MPCData) | hrl\_haptic\_mpc.haptic\_mpc.MPCData |  |
| **f\_l** (defined in hrl\_haptic\_mpc.haptic\_mpc.MPCData) | hrl\_haptic\_mpc.haptic\_mpc.MPCData |  |
| **f\_n** (defined in hrl\_haptic\_mpc.haptic\_mpc.MPCData) | hrl\_haptic\_mpc.haptic\_mpc.MPCData |  |
| **Jc\_l** (defined in hrl\_haptic\_mpc.haptic\_mpc.MPCData) | hrl\_haptic\_mpc.haptic\_mpc.MPCData |  |
| **Je** (defined in hrl\_haptic\_mpc.haptic\_mpc.MPCData) | hrl\_haptic\_mpc.haptic\_mpc.MPCData |  |
| **jep** (defined in hrl\_haptic\_mpc.haptic\_mpc.MPCData) | hrl\_haptic\_mpc.haptic\_mpc.MPCData |  |
| **jerk\_opt\_weight** (defined in hrl\_haptic\_mpc.haptic\_mpc.MPCData) | hrl\_haptic\_mpc.haptic\_mpc.MPCData |  |
| **K\_j** (defined in hrl\_haptic\_mpc.haptic\_mpc.MPCData) | hrl\_haptic\_mpc.haptic\_mpc.MPCData |  |
| **Kc\_l** (defined in hrl\_haptic\_mpc.haptic\_mpc.MPCData) | hrl\_haptic\_mpc.haptic\_mpc.MPCData |  |
| **loc\_l** (defined in hrl\_haptic\_mpc.haptic\_mpc.MPCData) | hrl\_haptic\_mpc.haptic\_mpc.MPCData |  |
| **max\_force\_mag** (defined in hrl\_haptic\_mpc.haptic\_mpc.MPCData) | hrl\_haptic\_mpc.haptic\_mpc.MPCData |  |
| **n\_l** (defined in hrl\_haptic\_mpc.haptic\_mpc.MPCData) | hrl\_haptic\_mpc.haptic\_mpc.MPCData |  |
| **orient\_weight** (defined in hrl\_haptic\_mpc.haptic\_mpc.MPCData) | hrl\_haptic\_mpc.haptic\_mpc.MPCData |  |
| **phi\_curr** (defined in hrl\_haptic\_mpc.haptic\_mpc.MPCData) | hrl\_haptic\_mpc.haptic\_mpc.MPCData |  |
| **position\_weight** (defined in hrl\_haptic\_mpc.haptic\_mpc.MPCData) | hrl\_haptic\_mpc.haptic\_mpc.MPCData |  |
| **q** (defined in hrl\_haptic\_mpc.haptic\_mpc.MPCData) | hrl\_haptic\_mpc.haptic\_mpc.MPCData |  |
| **q\_g\_orient** (defined in hrl\_haptic\_mpc.haptic\_mpc.MPCData) | hrl\_haptic\_mpc.haptic\_mpc.MPCData |  |
| **q\_h\_orient** (defined in hrl\_haptic\_mpc.haptic\_mpc.MPCData) | hrl\_haptic\_mpc.haptic\_mpc.MPCData |  |
| **Rc\_l** (defined in hrl\_haptic\_mpc.haptic\_mpc.MPCData) | hrl\_haptic\_mpc.haptic\_mpc.MPCData |  |
| **stop** (defined in hrl\_haptic\_mpc.haptic\_mpc.MPCData) | hrl\_haptic\_mpc.haptic\_mpc.MPCData |  |
| **time\_step** (defined in hrl\_haptic\_mpc.haptic\_mpc.MPCData) | hrl\_haptic\_mpc.haptic\_mpc.MPCData |  |
| **x\_g** (defined in hrl\_haptic\_mpc.haptic\_mpc.MPCData) | hrl\_haptic\_mpc.haptic\_mpc.MPCData |  |
| **x\_h** (defined in hrl\_haptic\_mpc.haptic\_mpc.MPCData) | hrl\_haptic\_mpc.haptic\_mpc.MPCData |  |


All Classes Namespaces Functions Variables

---

Generated on Thu Jan 17 2013 16:21:14 for HRL Haptic MPC by  

 1.7.6.1
