# Supplementary material for: In-home and remote use of robotic body surrogates by people with profound motor deficits
Source: PLoS One. 2019 Mar 15;14(3):e0212904. doi: 10.1371/journal.pone.0212904 (PMC6420017; doi:10.1371/journal.pone.0212904)
Supplement: S1 Source Code — This includes all custom code used in the described system and related evaluations. (ZIP) [file pone.0212904.s009.zip › robotic_body_surrogates_source_code/hrl-haptic-manip-pkgs.zip!/hrl-haptic-manip-pkgs/hrl_haptic_mpc/docs/html/classhrl__haptic__mpc_1_1haptic__mpc_1_1_m_p_c_data.html]

HRL Haptic MPC: hrl\_haptic\_mpc.haptic\_mpc.MPCData Class Reference


HRL Haptic MPC

A haptic model predictive controller

- Main Page
- Packages
- Classes

- Class List
- Class Index
- Class Members

All Classes Namespaces Functions Variables

- hrl\_haptic\_mpc
- **haptic\_mpc**
- MPCData

Public Member Functions |
Public Attributes

hrl\_haptic\_mpc.haptic\_mpc.MPCData Class Reference

Container class for the controller parameters.
More...

List of all members.

|  |  |
| --- | --- |
| Public Member Functions | |
| def | **\_\_init\_\_** |
| def | \_\_str\_\_ |
|  | String representation of the data structure. |
| Public Attributes | |
|  | **q** |
|  | **x\_h** |
|  | **x\_g** |
|  | **dist\_g** |
|  | **q\_h\_orient** |
|  | **q\_g\_orient** |
|  | **position\_weight** |
|  | **orient\_weight** |
|  | **control\_point\_joint\_num** |
|  | **Kc\_l** |
|  | **Rc\_l** |
|  | **Jc\_l** |
|  | **Je** |
|  | **delta\_f\_min** |
|  | **delta\_f\_max** |
|  | **phi\_curr** |
|  | **K\_j** |
|  | **loc\_l** |
|  | **n\_l** |
|  | **f\_l** |
|  | **f\_n** |
|  | **jerk\_opt\_weight** |
|  | **max\_force\_mag** |
|  | **jep** |
|  | **time\_step** |
|  | **stop** |

---

## Detailed Description

Container class for the controller parameters.

Some of these are parameters rather than control data, but they're small cf. the data which changes at each timestep.

---

## Member Function Documentation

|  |  |  |  |  |  |
| --- | --- | --- | --- | --- | --- |
| def hrl\_haptic\_mpc.haptic\_mpc.MPCData.\_\_str\_\_ | ( |  | *self* | ) |  |

String representation of the data structure.

Useful for debugging.

---

The documentation for this class was generated from the following file:

- /home/jdh/ros\_sandbox/hrl\_haptic\_manipulation\_in\_clutter/hrl\_haptic\_mpc/src/hrl\_haptic\_mpc/haptic\_mpc.py


All Classes Namespaces Functions Variables

---

Generated on Thu Jan 17 2013 16:21:14 for HRL Haptic MPC by  

 1.7.6.1
