# Supplementary material for: In-home and remote use of robotic body surrogates by people with profound motor deficits
Source: PLoS One. 2019 Mar 15;14(3):e0212904. doi: 10.1371/journal.pone.0212904 (PMC6420017; doi:10.1371/journal.pone.0212904)
Supplement: S1 Source Code — This includes all custom code used in the described system and related evaluations. (ZIP) [file pone.0212904.s009.zip › robotic_body_surrogates_source_code/hrl-haptic-manip-pkgs.zip!/hrl-haptic-manip-pkgs/hrl_haptic_mpc/docs/html/classhrl__haptic__mpc_1_1hrl__arm_1_1_h_r_l_arm.html]

HRL Haptic MPC: hrl\_haptic\_mpc.hrl\_arm.HRLArm Class Reference


HRL Haptic MPC

A haptic model predictive controller

- Main Page
- Packages
- Classes

- Class List
- Class Index
- Class Members

All Classes Namespaces Functions Variables

- hrl\_haptic\_mpc
- **hrl\_arm**
- HRLArm

Public Member Functions |
Public Attributes

hrl\_haptic\_mpc.hrl\_arm.HRLArm Class Reference

List of all members.

|  |  |
| --- | --- |
| Public Member Functions | |
| def | **\_\_init\_\_** |
| def | **get\_joint\_velocities** |
| def | **get\_joint\_angles** |
| def | **set\_ep** |
| def | **publish\_rviz\_markers** |
| def | **get\_ep** |
| def | **get\_joint\_impedance** |
| def | **get\_joint\_names** |
| def | **freeze** |
| def | **get\_end\_effector\_pose** |
| Public Attributes | |
|  | **kinematics** |
|  | **ep** |
|  | **kp** |
|  | **kd** |
|  | **q** |
|  | **qdot** |
|  | **joint\_names\_list** |
|  | **lock** |

---

The documentation for this class was generated from the following file:

- /home/jdh/ros\_sandbox/hrl\_haptic\_manipulation\_in\_clutter/hrl\_haptic\_mpc/src/hrl\_haptic\_mpc/hrl\_arm.py


All Classes Namespaces Functions Variables

---

Generated on Thu Jan 17 2013 16:21:14 for HRL Haptic MPC by  

 1.7.6.1
