# Supplementary material for: In-home and remote use of robotic body surrogates by people with profound motor deficits
Source: PLoS One. 2019 Mar 15;14(3):e0212904. doi: 10.1371/journal.pone.0212904 (PMC6420017; doi:10.1371/journal.pone.0212904)
Supplement: S1 Source Code — This includes all custom code used in the described system and related evaluations. (ZIP) [file pone.0212904.s009.zip › robotic_body_surrogates_source_code/hrl-haptic-manip-pkgs.zip!/hrl-haptic-manip-pkgs/hrl_haptic_mpc/docs/html/classhrl__haptic__mpc_1_1hrl__arm_1_1_h_r_l_arm_kinematics-members.html]

HRL Haptic MPC: Member List


HRL Haptic MPC

A haptic model predictive controller

- Main Page
- Packages
- Classes

- Class List
- Class Index
- Class Members

- hrl\_haptic\_mpc
- **hrl\_arm**
- HRLArmKinematics

hrl\_haptic\_mpc.hrl\_arm.HRLArmKinematics Member List

This is the complete list of members for hrl\_haptic\_mpc.hrl\_arm.HRLArmKinematics, including all inherited members.

|  |  |  |
| --- | --- | --- |
| **\_\_init\_\_** (defined in hrl\_haptic\_mpc.hrl\_arm.HRLArmKinematics) | hrl\_haptic\_mpc.hrl\_arm.HRLArmKinematics |  |
| **arm\_config\_to\_points\_list** (defined in hrl\_haptic\_mpc.hrl\_arm.HRLArmKinematics) | hrl\_haptic\_mpc.hrl\_arm.HRLArmKinematics |  |
| **distance\_from\_arm** (defined in hrl\_haptic\_mpc.hrl\_arm.HRLArmKinematics) | hrl\_haptic\_mpc.hrl\_arm.HRLArmKinematics |  |
| **distance\_from\_ee\_along\_arm** (defined in hrl\_haptic\_mpc.hrl\_arm.HRLArmKinematics) | hrl\_haptic\_mpc.hrl\_arm.HRLArmKinematics |  |
| **FK** (defined in hrl\_haptic\_mpc.hrl\_arm.HRLArmKinematics) | hrl\_haptic\_mpc.hrl\_arm.HRLArmKinematics |  |
| **FK\_vanilla** (defined in hrl\_haptic\_mpc.hrl\_arm.HRLArmKinematics) | hrl\_haptic\_mpc.hrl\_arm.HRLArmKinematics |  |
| get\_joint\_limits | hrl\_haptic\_mpc.hrl\_arm.HRLArmKinematics |  |
| IK | hrl\_haptic\_mpc.hrl\_arm.HRLArmKinematics |  |
| **IK** (defined in hrl\_haptic\_mpc.hrl\_arm.HRLArmKinematics) | hrl\_haptic\_mpc.hrl\_arm.HRLArmKinematics |  |
| **IK\_vanilla** (defined in hrl\_haptic\_mpc.hrl\_arm.HRLArmKinematics) | hrl\_haptic\_mpc.hrl\_arm.HRLArmKinematics |  |
| **is\_contact\_at\_joint** (defined in hrl\_haptic\_mpc.hrl\_arm.HRLArmKinematics) | hrl\_haptic\_mpc.hrl\_arm.HRLArmKinematics |  |
| jacobian | hrl\_haptic\_mpc.hrl\_arm.HRLArmKinematics |  |
| **n\_jts** (defined in hrl\_haptic\_mpc.hrl\_arm.HRLArmKinematics) | hrl\_haptic\_mpc.hrl\_arm.HRLArmKinematics |  |
| set\_tooltip | hrl\_haptic\_mpc.hrl\_arm.HRLArmKinematics |  |
| **tooltip\_pos** (defined in hrl\_haptic\_mpc.hrl\_arm.HRLArmKinematics) | hrl\_haptic\_mpc.hrl\_arm.HRLArmKinematics |  |
| **tooltip\_rot** (defined in hrl\_haptic\_mpc.hrl\_arm.HRLArmKinematics) | hrl\_haptic\_mpc.hrl\_arm.HRLArmKinematics |  |


All Classes Namespaces Functions Variables

---

Generated on Thu Jan 17 2013 16:21:14 for HRL Haptic MPC by  

 1.7.6.1
