# Supplementary material for: In-home and remote use of robotic body surrogates by people with profound motor deficits
Source: PLoS One. 2019 Mar 15;14(3):e0212904. doi: 10.1371/journal.pone.0212904 (PMC6420017; doi:10.1371/journal.pone.0212904)
Supplement: S1 Source Code — This includes all custom code used in the described system and related evaluations. (ZIP) [file pone.0212904.s009.zip › robotic_body_surrogates_source_code/hrl-haptic-manip-pkgs.zip!/hrl-haptic-manip-pkgs/hrl_haptic_mpc/docs/html/classhrl__haptic__mpc_1_1hrl__arm_1_1_h_r_l_arm_kinematics.html]

HRL Haptic MPC: hrl\_haptic\_mpc.hrl\_arm.HRLArmKinematics Class Reference


HRL Haptic MPC

A haptic model predictive controller

- Main Page
- Packages
- Classes

- Class List
- Class Index
- Class Members

All Classes Namespaces Functions Variables

- hrl\_haptic\_mpc
- **hrl\_arm**
- HRLArmKinematics

Public Member Functions |
Public Attributes

hrl\_haptic\_mpc.hrl\_arm.HRLArmKinematics Class Reference

List of all members.

|  |  |
| --- | --- |
| Public Member Functions | |
| def | **\_\_init\_\_** |
| def | **FK\_vanilla** |
| def | **FK** |
| def | IK |
|  | Computes IK for the tooltip. |
| def | **IK\_vanilla** |
| def | **IK** |
| def | jacobian |
|  | compute Jacobian at point pos. |
| def | get\_joint\_limits |
|  | return min\_array, max\_array |
| def | set\_tooltip |
|  | define tooltip as a 3x1 np matrix in the wrist coord frame. |
| def | **arm\_config\_to\_points\_list** |
| def | **distance\_from\_ee\_along\_arm** |
| def | **distance\_from\_arm** |
| def | **is\_contact\_at\_joint** |
| Public Attributes | |
|  | **tooltip\_pos** |
|  | **tooltip\_rot** |
|  | **n\_jts** |

---

## Member Function Documentation

|  |  |  |  |
| --- | --- | --- | --- |
| def hrl\_haptic\_mpc.hrl\_arm.HRLArmKinematics.IK | ( |  | *self*, |
|  |  |  | *pos*, |
|  |  |  | *rot*, |
|  |  |  | *q\_guess* = `None` |
|  | ) |  |  |

Computes IK for the tooltip.

The desired location is first transformed back into the last link's frame and IK is performed on that location.

**Parameters:**
:   |  |  |
    | --- | --- |
    | pos | Desired link position (3x1 np matrix) |
    | rot | Desired link rotation (3x3 np matrix) |
    | q\_guess | Estimate of the desired joint angles which seeds the IK solver |

|  |  |  |  |
| --- | --- | --- | --- |
| def hrl\_haptic\_mpc.hrl\_arm.HRLArmKinematics.jacobian | ( |  | *self*, |
|  |  |  | *q*, |
|  |  |  | *pos* = `None` |
|  | ) |  |  |

compute Jacobian at point pos.

|  |  |  |  |
| --- | --- | --- | --- |
| def hrl\_haptic\_mpc.hrl\_arm.HRLArmKinematics.set\_tooltip | ( |  | *self*, |
|  |  |  | *p*, |
|  |  |  | *rot* = `np.matrix(np.eye(3))` |
|  | ) |  |  |

define tooltip as a 3x1 np matrix in the wrist coord frame.

---

The documentation for this class was generated from the following file:

- /home/jdh/ros\_sandbox/hrl\_haptic\_manipulation\_in\_clutter/hrl\_haptic\_mpc/src/hrl\_haptic\_mpc/hrl\_arm.py


All Classes Namespaces Functions Variables

---

Generated on Thu Jan 17 2013 16:21:14 for HRL Haptic MPC by  

 1.7.6.1
