# Supplementary material for: In-home and remote use of robotic body surrogates by people with profound motor deficits
Source: PLoS One. 2019 Mar 15;14(3):e0212904. doi: 10.1371/journal.pone.0212904 (PMC6420017; doi:10.1371/journal.pone.0212904)
Supplement: S1 Source Code — This includes all custom code used in the described system and related evaluations. (ZIP) [file pone.0212904.s009.zip › robotic_body_surrogates_source_code/hrl-haptic-manip-pkgs.zip!/hrl-haptic-manip-pkgs/hrl_haptic_mpc/docs/html/classhrl__haptic__mpc_1_1mpc__teleop__rviz_1_1_m_p_c_teleop_interactive_markers-members.html]

HRL Haptic MPC: Member List


HRL Haptic MPC

A haptic model predictive controller

- Main Page
- Packages
- Classes

- Class List
- Class Index
- Class Members

- hrl\_haptic\_mpc
- **mpc\_teleop\_rviz**
- MPCTeleopInteractiveMarkers

hrl\_haptic\_mpc.mpc\_teleop\_rviz.MPCTeleopInteractiveMarkers Member List

This is the complete list of members for hrl\_haptic\_mpc.mpc\_teleop\_rviz.MPCTeleopInteractiveMarkers, including all inherited members.

|  |  |  |
| --- | --- | --- |
| **\_\_init\_\_** (defined in hrl\_haptic\_mpc.mpc\_teleop\_rviz.MPCTeleopInteractiveMarkers) | hrl\_haptic\_mpc.mpc\_teleop\_rviz.MPCTeleopInteractiveMarkers |  |
| **add\_topic\_pub** (defined in hrl\_haptic\_mpc.mpc\_teleop\_rviz.MPCTeleopInteractiveMarkers) | hrl\_haptic\_mpc.mpc\_teleop\_rviz.MPCTeleopInteractiveMarkers |  |
| **close\_pub** (defined in hrl\_haptic\_mpc.mpc\_teleop\_rviz.MPCTeleopInteractiveMarkers) | hrl\_haptic\_mpc.mpc\_teleop\_rviz.MPCTeleopInteractiveMarkers |  |
| closeGripperHandler | hrl\_haptic\_mpc.mpc\_teleop\_rviz.MPCTeleopInteractiveMarkers |  |
| **current\_goal\_pose** (defined in hrl\_haptic\_mpc.mpc\_teleop\_rviz.MPCTeleopInteractiveMarkers) | hrl\_haptic\_mpc.mpc\_teleop\_rviz.MPCTeleopInteractiveMarkers |  |
| **disable\_pub** (defined in hrl\_haptic\_mpc.mpc\_teleop\_rviz.MPCTeleopInteractiveMarkers) | hrl\_haptic\_mpc.mpc\_teleop\_rviz.MPCTeleopInteractiveMarkers |  |
| disablePps | hrl\_haptic\_mpc.mpc\_teleop\_rviz.MPCTeleopInteractiveMarkers |  |
| **enable\_pub** (defined in hrl\_haptic\_mpc.mpc\_teleop\_rviz.MPCTeleopInteractiveMarkers) | hrl\_haptic\_mpc.mpc\_teleop\_rviz.MPCTeleopInteractiveMarkers |  |
| enablePps | hrl\_haptic\_mpc.mpc\_teleop\_rviz.MPCTeleopInteractiveMarkers |  |
| **goal\_feedback\_rviz\_cb** (defined in hrl\_haptic\_mpc.mpc\_teleop\_rviz.MPCTeleopInteractiveMarkers) | hrl\_haptic\_mpc.mpc\_teleop\_rviz.MPCTeleopInteractiveMarkers |  |
| **goal\_pos\_pub** (defined in hrl\_haptic\_mpc.mpc\_teleop\_rviz.MPCTeleopInteractiveMarkers) | hrl\_haptic\_mpc.mpc\_teleop\_rviz.MPCTeleopInteractiveMarkers |  |
| **goal\_traj\_pub** (defined in hrl\_haptic\_mpc.mpc\_teleop\_rviz.MPCTeleopInteractiveMarkers) | hrl\_haptic\_mpc.mpc\_teleop\_rviz.MPCTeleopInteractiveMarkers |  |
| goalPositionHandler | hrl\_haptic\_mpc.mpc\_teleop\_rviz.MPCTeleopInteractiveMarkers |  |
| goalPositionOrientationHandler | hrl\_haptic\_mpc.mpc\_teleop\_rviz.MPCTeleopInteractiveMarkers |  |
| initComms | hrl\_haptic\_mpc.mpc\_teleop\_rviz.MPCTeleopInteractiveMarkers |  |
| initMarkers | hrl\_haptic\_mpc.mpc\_teleop\_rviz.MPCTeleopInteractiveMarkers |  |
| initMenu | hrl\_haptic\_mpc.mpc\_teleop\_rviz.MPCTeleopInteractiveMarkers |  |
| interactiveMarkerLocationCallback | hrl\_haptic\_mpc.mpc\_teleop\_rviz.MPCTeleopInteractiveMarkers |  |
| **mpc\_weights\_pub** (defined in hrl\_haptic\_mpc.mpc\_teleop\_rviz.MPCTeleopInteractiveMarkers) | hrl\_haptic\_mpc.mpc\_teleop\_rviz.MPCTeleopInteractiveMarkers |  |
| **open\_pub** (defined in hrl\_haptic\_mpc.mpc\_teleop\_rviz.MPCTeleopInteractiveMarkers) | hrl\_haptic\_mpc.mpc\_teleop\_rviz.MPCTeleopInteractiveMarkers |  |
| openGripperHandler | hrl\_haptic\_mpc.mpc\_teleop\_rviz.MPCTeleopInteractiveMarkers |  |
| **opt** (defined in hrl\_haptic\_mpc.mpc\_teleop\_rviz.MPCTeleopInteractiveMarkers) | hrl\_haptic\_mpc.mpc\_teleop\_rviz.MPCTeleopInteractiveMarkers |  |
| **remove\_topic\_pub** (defined in hrl\_haptic\_mpc.mpc\_teleop\_rviz.MPCTeleopInteractiveMarkers) | hrl\_haptic\_mpc.mpc\_teleop\_rviz.MPCTeleopInteractiveMarkers |  |
| **server** (defined in hrl\_haptic\_mpc.mpc\_teleop\_rviz.MPCTeleopInteractiveMarkers) | hrl\_haptic\_mpc.mpc\_teleop\_rviz.MPCTeleopInteractiveMarkers |  |
| start | hrl\_haptic\_mpc.mpc\_teleop\_rviz.MPCTeleopInteractiveMarkers |  |
| stopArmHandler | hrl\_haptic\_mpc.mpc\_teleop\_rviz.MPCTeleopInteractiveMarkers |  |
| **wp\_im** (defined in hrl\_haptic\_mpc.mpc\_teleop\_rviz.MPCTeleopInteractiveMarkers) | hrl\_haptic\_mpc.mpc\_teleop\_rviz.MPCTeleopInteractiveMarkers |  |
| **wp\_menu\_handler** (defined in hrl\_haptic\_mpc.mpc\_teleop\_rviz.MPCTeleopInteractiveMarkers) | hrl\_haptic\_mpc.mpc\_teleop\_rviz.MPCTeleopInteractiveMarkers |  |
| **zero\_forearm\_pub** (defined in hrl\_haptic\_mpc.mpc\_teleop\_rviz.MPCTeleopInteractiveMarkers) | hrl\_haptic\_mpc.mpc\_teleop\_rviz.MPCTeleopInteractiveMarkers |  |
| **zero\_gripper\_left\_link\_pub** (defined in hrl\_haptic\_mpc.mpc\_teleop\_rviz.MPCTeleopInteractiveMarkers) | hrl\_haptic\_mpc.mpc\_teleop\_rviz.MPCTeleopInteractiveMarkers |  |
| **zero\_gripper\_palm\_pub** (defined in hrl\_haptic\_mpc.mpc\_teleop\_rviz.MPCTeleopInteractiveMarkers) | hrl\_haptic\_mpc.mpc\_teleop\_rviz.MPCTeleopInteractiveMarkers |  |
| **zero\_gripper\_pub** (defined in hrl\_haptic\_mpc.mpc\_teleop\_rviz.MPCTeleopInteractiveMarkers) | hrl\_haptic\_mpc.mpc\_teleop\_rviz.MPCTeleopInteractiveMarkers |  |
| **zero\_gripper\_right\_link\_pub** (defined in hrl\_haptic\_mpc.mpc\_teleop\_rviz.MPCTeleopInteractiveMarkers) | hrl\_haptic\_mpc.mpc\_teleop\_rviz.MPCTeleopInteractiveMarkers |  |
| **zero\_pps\_left\_pub** (defined in hrl\_haptic\_mpc.mpc\_teleop\_rviz.MPCTeleopInteractiveMarkers) | hrl\_haptic\_mpc.mpc\_teleop\_rviz.MPCTeleopInteractiveMarkers |  |
| **zero\_pps\_right\_pub** (defined in hrl\_haptic\_mpc.mpc\_teleop\_rviz.MPCTeleopInteractiveMarkers) | hrl\_haptic\_mpc.mpc\_teleop\_rviz.MPCTeleopInteractiveMarkers |  |
| **zero\_upperarm\_pub** (defined in hrl\_haptic\_mpc.mpc\_teleop\_rviz.MPCTeleopInteractiveMarkers) | hrl\_haptic\_mpc.mpc\_teleop\_rviz.MPCTeleopInteractiveMarkers |  |
| zeroSkinHandler | hrl\_haptic\_mpc.mpc\_teleop\_rviz.MPCTeleopInteractiveMarkers |  |


All Classes Namespaces Functions Variables

---

Generated on Thu Jan 17 2013 16:21:14 for HRL Haptic MPC by  

 1.7.6.1
