# Supplementary material for: In-home and remote use of robotic body surrogates by people with profound motor deficits
Source: PLoS One. 2019 Mar 15;14(3):e0212904. doi: 10.1371/journal.pone.0212904 (PMC6420017; doi:10.1371/journal.pone.0212904)
Supplement: S1 Source Code — This includes all custom code used in the described system and related evaluations. (ZIP) [file pone.0212904.s009.zip › robotic_body_surrogates_source_code/hrl-haptic-manip-pkgs.zip!/hrl-haptic-manip-pkgs/hrl_haptic_mpc/docs/html/classhrl__haptic__mpc_1_1mpc__teleop__rviz_1_1_m_p_c_teleop_interactive_markers.html]

HRL Haptic MPC: hrl\_haptic\_mpc.mpc\_teleop\_rviz.MPCTeleopInteractiveMarkers Class Reference


HRL Haptic MPC

A haptic model predictive controller

- Main Page
- Packages
- Classes

- Class List
- Class Index
- Class Members

All Classes Namespaces Functions Variables

- hrl\_haptic\_mpc
- **mpc\_teleop\_rviz**
- MPCTeleopInteractiveMarkers

Public Member Functions |
Public Attributes

hrl\_haptic\_mpc.mpc\_teleop\_rviz.MPCTeleopInteractiveMarkers Class Reference

RViz teleop interface to the controller.
More...

List of all members.

|  |  |
| --- | --- |
| Public Member Functions | |
| def | **\_\_init\_\_** |
| def | interactiveMarkerLocationCallback |
|  | Callback for the interactive marker location. |
| def | goalPositionHandler |
|  | Publishes the current pose of the interactive marker as the goal pose for the MPC. |
| def | goalPositionOrientationHandler |
|  | Publishes the current pose of the interactive marker as the goal pose for the MPC. |
| def | stopArmHandler |
|  | Publishes an empty trajectory message. |
| def | enablePps |
|  | Enable the PR2 PPS sensors by adding the topics to the skin client. |
| def | disablePps |
|  | Enable the PR2 PPS sensors by removing the topics from the skin client. |
| def | openGripperHandler |
|  | Open the PR2 gripper for this arm and enable the PPS sensors (assumes this is running on the PR2). |
| def | closeGripperHandler |
|  | Close the PR2 gripper for this arm and enable the PPS sensors (assumes this is running on the PR2). |
| def | zeroSkinHandler |
|  | Zeroes the PR2 skin (eg, to correct for calibration errors). |
| def | **goal\_feedback\_rviz\_cb** |
| def | initComms |
|  | Initialise all publishers/subscribers. |
| def | initMarkers |
|  | Initialise the interactive marker based on what robot we're running on, and whether we use orientation or just position. |
| def | initMenu |
|  | Initialise the menu used to control the arm behaviour. |
| def | start |
|  | Start the interactive marker server (spin indefinitely). |
| Public Attributes | |
|  | **opt** |
|  | **current\_goal\_pose** |
|  | **goal\_pos\_pub** |
|  | **mpc\_weights\_pub** |
|  | **goal\_traj\_pub** |
|  | **add\_topic\_pub** |
|  | **remove\_topic\_pub** |
|  | **open\_pub** |
|  | **close\_pub** |
|  | **disable\_pub** |
|  | **enable\_pub** |
|  | **zero\_gripper\_pub** |
|  | **zero\_gripper\_left\_link\_pub** |
|  | **zero\_gripper\_right\_link\_pub** |
|  | **zero\_gripper\_palm\_pub** |
|  | **zero\_forearm\_pub** |
|  | **zero\_upperarm\_pub** |
|  | **zero\_pps\_left\_pub** |
|  | **zero\_pps\_right\_pub** |
|  | **server** |
|  | **wp\_im** |
|  | **wp\_menu\_handler** |

---

## Detailed Description

RViz teleop interface to the controller.

Publishes goal poses on the appropriate topics and allows a user to teleop the arm controller.

---

## Member Function Documentation

|  |  |  |  |
| --- | --- | --- | --- |
| def hrl\_haptic\_mpc.mpc\_teleop\_rviz.MPCTeleopInteractiveMarkers.closeGripperHandler | ( |  | *self*, |
|  |  |  | *feedback* |
|  | ) |  |  |

Close the PR2 gripper for this arm and enable the PPS sensors (assumes this is running on the PR2).

|  |  |  |  |  |  |
| --- | --- | --- | --- | --- | --- |
| def hrl\_haptic\_mpc.mpc\_teleop\_rviz.MPCTeleopInteractiveMarkers.disablePps | ( |  | *self* | ) |  |

Enable the PR2 PPS sensors by removing the topics from the skin client.

|  |  |  |  |  |  |
| --- | --- | --- | --- | --- | --- |
| def hrl\_haptic\_mpc.mpc\_teleop\_rviz.MPCTeleopInteractiveMarkers.enablePps | ( |  | *self* | ) |  |

Enable the PR2 PPS sensors by adding the topics to the skin client.

|  |  |  |  |
| --- | --- | --- | --- |
| def hrl\_haptic\_mpc.mpc\_teleop\_rviz.MPCTeleopInteractiveMarkers.goalPositionHandler | ( |  | *self*, |
|  |  |  | *feedback* |
|  | ) |  |  |

Publishes the current pose of the interactive marker as the goal pose for the MPC.

Also sets the orientation weight for the controller to 0.0 (ie, position only).

|  |  |  |  |
| --- | --- | --- | --- |
| def hrl\_haptic\_mpc.mpc\_teleop\_rviz.MPCTeleopInteractiveMarkers.goalPositionOrientationHandler | ( |  | *self*, |
|  |  |  | *feedback* |
|  | ) |  |  |

Publishes the current pose of the interactive marker as the goal pose for the MPC.

Also enables the orientation weight (ie, position and orientation).

|  |  |  |  |  |  |
| --- | --- | --- | --- | --- | --- |
| def hrl\_haptic\_mpc.mpc\_teleop\_rviz.MPCTeleopInteractiveMarkers.initMarkers | ( |  | *self* | ) |  |

Initialise the interactive marker based on what robot we're running on, and whether we use orientation or just position.

|  |  |  |  |  |  |
| --- | --- | --- | --- | --- | --- |
| def hrl\_haptic\_mpc.mpc\_teleop\_rviz.MPCTeleopInteractiveMarkers.initMenu | ( |  | *self* | ) |  |

Initialise the menu used to control the arm behaviour.

|  |  |  |  |
| --- | --- | --- | --- |
| def hrl\_haptic\_mpc.mpc\_teleop\_rviz.MPCTeleopInteractiveMarkers.interactiveMarkerLocationCallback | ( |  | *self*, |
|  |  |  | *feedback* |
|  | ) |  |  |

Callback for the interactive marker location.

Receives and stores the updated pose of the marker in space as the user moves it around.

|  |  |  |  |
| --- | --- | --- | --- |
| def hrl\_haptic\_mpc.mpc\_teleop\_rviz.MPCTeleopInteractiveMarkers.openGripperHandler | ( |  | *self*, |
|  |  |  | *feedback* |
|  | ) |  |  |

Open the PR2 gripper for this arm and enable the PPS sensors (assumes this is running on the PR2).

|  |  |  |  |  |  |
| --- | --- | --- | --- | --- | --- |
| def hrl\_haptic\_mpc.mpc\_teleop\_rviz.MPCTeleopInteractiveMarkers.start | ( |  | *self* | ) |  |

Start the interactive marker server (spin indefinitely).

|  |  |  |  |
| --- | --- | --- | --- |
| def hrl\_haptic\_mpc.mpc\_teleop\_rviz.MPCTeleopInteractiveMarkers.stopArmHandler | ( |  | *self*, |
|  |  |  | *feedback* |
|  | ) |  |  |

Publishes an empty trajectory message.

This has the effect of flushing the stored trajectory and goal pose from the waypoint generator, stopping the controller motion.

|  |  |  |  |
| --- | --- | --- | --- |
| def hrl\_haptic\_mpc.mpc\_teleop\_rviz.MPCTeleopInteractiveMarkers.zeroSkinHandler | ( |  | *self*, |
|  |  |  | *feedback* |
|  | ) |  |  |

Zeroes the PR2 skin (eg, to correct for calibration errors).

---

The documentation for this class was generated from the following file:

- /home/jdh/ros\_sandbox/hrl\_haptic\_manipulation\_in\_clutter/hrl\_haptic\_mpc/src/hrl\_haptic\_mpc/mpc\_teleop\_rviz.py


All Classes Namespaces Functions Variables

---

Generated on Thu Jan 17 2013 16:21:14 for HRL Haptic MPC by  

 1.7.6.1
