# Supplementary material for: In-home and remote use of robotic body surrogates by people with profound motor deficits
Source: PLoS One. 2019 Mar 15;14(3):e0212904. doi: 10.1371/journal.pone.0212904 (PMC6420017; doi:10.1371/journal.pone.0212904)
Supplement: S1 Source Code — This includes all custom code used in the described system and related evaluations. (ZIP) [file pone.0212904.s009.zip › robotic_body_surrogates_source_code/hrl-haptic-manip-pkgs.zip!/hrl-haptic-manip-pkgs/hrl_haptic_mpc/docs/html/classhrl__haptic__mpc_1_1multiarray__to__matrix_1_1_multi_array_converter.html]

HRL Haptic MPC: hrl\_haptic\_mpc.multiarray\_to\_matrix.MultiArrayConverter Class Reference


HRL Haptic MPC

A haptic model predictive controller

- Main Page
- Packages
- Classes

- Class List
- Class Index
- Class Members

All Classes Namespaces Functions Variables

- hrl\_haptic\_mpc
- **multiarray\_to\_matrix**
- MultiArrayConverter

Public Member Functions

hrl\_haptic\_mpc.multiarray\_to\_matrix.MultiArrayConverter Class Reference

Helper class to convert numpy matrix lists to and from a Float64MultiArray message object.
More...

List of all members.

|  |  |
| --- | --- |
| Public Member Functions | |
| def | multiArrayToMatrixList |
|  | Take a Float64 MultiArray message, convert it into a list of numpy matrices. |
| def | matrixListToMultiarray |
|  | Convert a list of 2D numpy matrices to a Float64MultiArray message. |

---

## Detailed Description

Helper class to convert numpy matrix lists to and from a Float64MultiArray message object.

---

## Member Function Documentation

|  |  |  |  |
| --- | --- | --- | --- |
| def hrl\_haptic\_mpc.multiarray\_to\_matrix.MultiArrayConverter.matrixListToMultiarray | ( |  | *self*, |
|  |  |  | *matrix\_list* |
|  | ) |  |  |

Convert a list of 2D numpy matrices to a Float64MultiArray message.

Assumption: Each numpy matrix must be the same size (rows, columns)

---

The documentation for this class was generated from the following file:

- /home/jdh/ros\_sandbox/hrl\_haptic\_manipulation\_in\_clutter/hrl\_haptic\_mpc/src/hrl\_haptic\_mpc/multiarray\_to\_matrix.py


All Classes Namespaces Functions Variables

---

Generated on Thu Jan 17 2013 16:21:14 for HRL Haptic MPC by  

 1.7.6.1
