# Supplementary material for: In-home and remote use of robotic body surrogates by people with profound motor deficits
Source: PLoS One. 2019 Mar 15;14(3):e0212904. doi: 10.1371/journal.pone.0212904 (PMC6420017; doi:10.1371/journal.pone.0212904)
Supplement: S1 Source Code — This includes all custom code used in the described system and related evaluations. (ZIP) [file pone.0212904.s009.zip › robotic_body_surrogates_source_code/hrl-haptic-manip-pkgs.zip!/hrl-haptic-manip-pkgs/hrl_haptic_mpc/docs/html/classhrl__haptic__mpc_1_1pr2__skin__client_1_1_p_r2___skin_client.html]

HRL Haptic MPC: hrl\_haptic\_mpc.pr2\_skin\_client.PR2\_SkinClient Class Reference


HRL Haptic MPC

A haptic model predictive controller

- Main Page
- Packages
- Classes

- Class List
- Class Index
- Class Members

All Classes Namespaces Functions Variables

- hrl\_haptic\_mpc
- **pr2\_skin\_client**
- PR2\_SkinClient

Public Member Functions

hrl\_haptic\_mpc.pr2\_skin\_client.PR2\_SkinClient Class Reference

PR2 specific implementation of the TaxelArrayClient class.
More...

List of all members.

|  |  |
| --- | --- |
| Public Member Functions | |
| def | **\_\_init\_\_** |
| def | getTaxelLocationAndJointList |
|  | Returns a list of taxel locations and a list of joint numbers after which the joint torque will have no effect on the contact force. |

---

## Detailed Description

PR2 specific implementation of the TaxelArrayClient class.

Adds one function which needs to know about PR2 specific parameters. This structure isn't fantastic and will eventually be pulled into the robot haptic state node as it's only used to form the contact jacobians.

---

## Member Function Documentation

|  |  |  |  |
| --- | --- | --- | --- |
| def hrl\_haptic\_mpc.pr2\_skin\_client.PR2\_SkinClient.getTaxelLocationAndJointList | ( |  | *self*, |
|  |  |  | *skin\_data* |
|  | ) |  |  |

Returns a list of taxel locations and a list of joint numbers after which the joint torque will have no effect on the contact force.

**Parameters:**
:   |  |  |
    | --- | --- |
    | skin\_data | Dictionary of TaxelArrays indexed by topic |

**Return values:**
:   |  |  |
    | --- | --- |
    | locations | List of taxel locations where a force is present |
    | joint\_nums | List of joints after which the joint torque will have no effect on the contact force |

**Returns:**
:   These arrays will both be the same length (as the joint number corresponds

---

The documentation for this class was generated from the following file:

- /home/jdh/ros\_sandbox/hrl\_haptic\_manipulation\_in\_clutter/hrl\_haptic\_mpc/src/hrl\_haptic\_mpc/pr2\_skin\_client.py


All Classes Namespaces Functions Variables

---

Generated on Thu Jan 17 2013 16:21:14 for HRL Haptic MPC by  

 1.7.6.1
