# Supplementary material for: In-home and remote use of robotic body surrogates by people with profound motor deficits
Source: PLoS One. 2019 Mar 15;14(3):e0212904. doi: 10.1371/journal.pone.0212904 (PMC6420017; doi:10.1371/journal.pone.0212904)
Supplement: S1 Source Code — This includes all custom code used in the described system and related evaluations. (ZIP) [file pone.0212904.s009.zip › robotic_body_surrogates_source_code/hrl-haptic-manip-pkgs.zip!/hrl-haptic-manip-pkgs/hrl_haptic_mpc/docs/html/classhrl__haptic__mpc_1_1robot__haptic__state__node_1_1_robot_haptic_state_server-members.html]

HRL Haptic MPC: Member List


HRL Haptic MPC

A haptic model predictive controller

- Main Page
- Packages
- Classes

- Class List
- Class Index
- Class Members

- hrl\_haptic\_mpc
- **robot\_haptic\_state\_node**
- RobotHapticStateServer

hrl\_haptic\_mpc.robot\_haptic\_state\_node.RobotHapticStateServer Member List

This is the complete list of members for hrl\_haptic\_mpc.robot\_haptic\_state\_node.RobotHapticStateServer, including all inherited members.

|  |  |  |
| --- | --- | --- |
| \_\_init\_\_ | hrl\_haptic\_mpc.robot\_haptic\_state\_node.RobotHapticStateServer |  |
| **base\_path** (defined in hrl\_haptic\_mpc.robot\_haptic\_state\_node.RobotHapticStateServer) | hrl\_haptic\_mpc.robot\_haptic\_state\_node.RobotHapticStateServer |  |
| **desired\_joint\_angles** (defined in hrl\_haptic\_mpc.robot\_haptic\_state\_node.RobotHapticStateServer) | hrl\_haptic\_mpc.robot\_haptic\_state\_node.RobotHapticStateServer |  |
| **end\_effector\_orient\_cart** (defined in hrl\_haptic\_mpc.robot\_haptic\_state\_node.RobotHapticStateServer) | hrl\_haptic\_mpc.robot\_haptic\_state\_node.RobotHapticStateServer |  |
| **end\_effector\_orient\_quat** (defined in hrl\_haptic\_mpc.robot\_haptic\_state\_node.RobotHapticStateServer) | hrl\_haptic\_mpc.robot\_haptic\_state\_node.RobotHapticStateServer |  |
| **end\_effector\_position** (defined in hrl\_haptic\_mpc.robot\_haptic\_state\_node.RobotHapticStateServer) | hrl\_haptic\_mpc.robot\_haptic\_state\_node.RobotHapticStateServer |  |
| **getMessageHeader** (defined in hrl\_haptic\_mpc.robot\_haptic\_state\_node.RobotHapticStateServer) | hrl\_haptic\_mpc.robot\_haptic\_state\_node.RobotHapticStateServer |  |
| **gripper\_pose\_pub** (defined in hrl\_haptic\_mpc.robot\_haptic\_state\_node.RobotHapticStateServer) | hrl\_haptic\_mpc.robot\_haptic\_state\_node.RobotHapticStateServer |  |
| **inertial\_frame** (defined in hrl\_haptic\_mpc.robot\_haptic\_state\_node.RobotHapticStateServer) | hrl\_haptic\_mpc.robot\_haptic\_state\_node.RobotHapticStateServer |  |
| initCody | hrl\_haptic\_mpc.robot\_haptic\_state\_node.RobotHapticStateServer |  |
| **initComms** (defined in hrl\_haptic\_mpc.robot\_haptic\_state\_node.RobotHapticStateServer) | hrl\_haptic\_mpc.robot\_haptic\_state\_node.RobotHapticStateServer |  |
| initPR2 | hrl\_haptic\_mpc.robot\_haptic\_state\_node.RobotHapticStateServer |  |
| initRobot | hrl\_haptic\_mpc.robot\_haptic\_state\_node.RobotHapticStateServer |  |
| initSim | hrl\_haptic\_mpc.robot\_haptic\_state\_node.RobotHapticStateServer |  |
| **initSimCody** (defined in hrl\_haptic\_mpc.robot\_haptic\_state\_node.RobotHapticStateServer) | hrl\_haptic\_mpc.robot\_haptic\_state\_node.RobotHapticStateServer |  |
| **Jc** (defined in hrl\_haptic\_mpc.robot\_haptic\_state\_node.RobotHapticStateServer) | hrl\_haptic\_mpc.robot\_haptic\_state\_node.RobotHapticStateServer |  |
| **Je** (defined in hrl\_haptic\_mpc.robot\_haptic\_state\_node.RobotHapticStateServer) | hrl\_haptic\_mpc.robot\_haptic\_state\_node.RobotHapticStateServer |  |
| **joint\_angles** (defined in hrl\_haptic\_mpc.robot\_haptic\_state\_node.RobotHapticStateServer) | hrl\_haptic\_mpc.robot\_haptic\_state\_node.RobotHapticStateServer |  |
| **joint\_damping** (defined in hrl\_haptic\_mpc.robot\_haptic\_state\_node.RobotHapticStateServer) | hrl\_haptic\_mpc.robot\_haptic\_state\_node.RobotHapticStateServer |  |
| **joint\_data\_lock** (defined in hrl\_haptic\_mpc.robot\_haptic\_state\_node.RobotHapticStateServer) | hrl\_haptic\_mpc.robot\_haptic\_state\_node.RobotHapticStateServer |  |
| **joint\_limits\_max** (defined in hrl\_haptic\_mpc.robot\_haptic\_state\_node.RobotHapticStateServer) | hrl\_haptic\_mpc.robot\_haptic\_state\_node.RobotHapticStateServer |  |
| **joint\_limits\_min** (defined in hrl\_haptic\_mpc.robot\_haptic\_state\_node.RobotHapticStateServer) | hrl\_haptic\_mpc.robot\_haptic\_state\_node.RobotHapticStateServer |  |
| joint\_names | hrl\_haptic\_mpc.robot\_haptic\_state\_node.RobotHapticStateServer |  |
| **joint\_stiffness** (defined in hrl\_haptic\_mpc.robot\_haptic\_state\_node.RobotHapticStateServer) | hrl\_haptic\_mpc.robot\_haptic\_state\_node.RobotHapticStateServer |  |
| **joint\_velocities** (defined in hrl\_haptic\_mpc.robot\_haptic\_state\_node.RobotHapticStateServer) | hrl\_haptic\_mpc.robot\_haptic\_state\_node.RobotHapticStateServer |  |
| **ma\_to\_m** (defined in hrl\_haptic\_mpc.robot\_haptic\_state\_node.RobotHapticStateServer) | hrl\_haptic\_mpc.robot\_haptic\_state\_node.RobotHapticStateServer |  |
| modifyPR2Taxels | hrl\_haptic\_mpc.robot\_haptic\_state\_node.RobotHapticStateServer |  |
| modifyRobotSpecificTaxels | hrl\_haptic\_mpc.robot\_haptic\_state\_node.RobotHapticStateServer |  |
| **msg\_seq** (defined in hrl\_haptic\_mpc.robot\_haptic\_state\_node.RobotHapticStateServer) | hrl\_haptic\_mpc.robot\_haptic\_state\_node.RobotHapticStateServer |  |
| **node\_name** (defined in hrl\_haptic\_mpc.robot\_haptic\_state\_node.RobotHapticStateServer) | hrl\_haptic\_mpc.robot\_haptic\_state\_node.RobotHapticStateServer |  |
| **opt** (defined in hrl\_haptic\_mpc.robot\_haptic\_state\_node.RobotHapticStateServer) | hrl\_haptic\_mpc.robot\_haptic\_state\_node.RobotHapticStateServer |  |
| publishRobotState | hrl\_haptic\_mpc.robot\_haptic\_state\_node.RobotHapticStateServer |  |
| **rate** (defined in hrl\_haptic\_mpc.robot\_haptic\_state\_node.RobotHapticStateServer) | hrl\_haptic\_mpc.robot\_haptic\_state\_node.RobotHapticStateServer |  |
| **robot** (defined in hrl\_haptic\_mpc.robot\_haptic\_state\_node.RobotHapticStateServer) | hrl\_haptic\_mpc.robot\_haptic\_state\_node.RobotHapticStateServer |  |
| **robot\_path** (defined in hrl\_haptic\_mpc.robot\_haptic\_state\_node.RobotHapticStateServer) | hrl\_haptic\_mpc.robot\_haptic\_state\_node.RobotHapticStateServer |  |
| **skin\_client** (defined in hrl\_haptic\_mpc.robot\_haptic\_state\_node.RobotHapticStateServer) | hrl\_haptic\_mpc.robot\_haptic\_state\_node.RobotHapticStateServer |  |
| **skin\_topic\_list** (defined in hrl\_haptic\_mpc.robot\_haptic\_state\_node.RobotHapticStateServer) | hrl\_haptic\_mpc.robot\_haptic\_state\_node.RobotHapticStateServer |  |
| start | hrl\_haptic\_mpc.robot\_haptic\_state\_node.RobotHapticStateServer |  |
| **state\_pub** (defined in hrl\_haptic\_mpc.robot\_haptic\_state\_node.RobotHapticStateServer) | hrl\_haptic\_mpc.robot\_haptic\_state\_node.RobotHapticStateServer |  |
| **tf\_listener** (defined in hrl\_haptic\_mpc.robot\_haptic\_state\_node.RobotHapticStateServer) | hrl\_haptic\_mpc.robot\_haptic\_state\_node.RobotHapticStateServer |  |
| **torso\_frame** (defined in hrl\_haptic\_mpc.robot\_haptic\_state\_node.RobotHapticStateServer) | hrl\_haptic\_mpc.robot\_haptic\_state\_node.RobotHapticStateServer |  |
| **trim\_threshold** (defined in hrl\_haptic\_mpc.robot\_haptic\_state\_node.RobotHapticStateServer) | hrl\_haptic\_mpc.robot\_haptic\_state\_node.RobotHapticStateServer |  |
| updateContactJacobians | hrl\_haptic\_mpc.robot\_haptic\_state\_node.RobotHapticStateServer |  |
| **updateEndEffectorJacobian** (defined in hrl\_haptic\_mpc.robot\_haptic\_state\_node.RobotHapticStateServer) | hrl\_haptic\_mpc.robot\_haptic\_state\_node.RobotHapticStateServer |  |
| **updateEndEffectorPose** (defined in hrl\_haptic\_mpc.robot\_haptic\_state\_node.RobotHapticStateServer) | hrl\_haptic\_mpc.robot\_haptic\_state\_node.RobotHapticStateServer |  |
| **updateJointStates** (defined in hrl\_haptic\_mpc.robot\_haptic\_state\_node.RobotHapticStateServer) | hrl\_haptic\_mpc.robot\_haptic\_state\_node.RobotHapticStateServer |  |
| updateTorsoPose | hrl\_haptic\_mpc.robot\_haptic\_state\_node.RobotHapticStateServer |  |


All Classes Namespaces Functions Variables

---

Generated on Thu Jan 17 2013 16:21:14 for HRL Haptic MPC by  

 1.7.6.1
