# Supplementary material for: In-home and remote use of robotic body surrogates by people with profound motor deficits
Source: PLoS One. 2019 Mar 15;14(3):e0212904. doi: 10.1371/journal.pone.0212904 (PMC6420017; doi:10.1371/journal.pone.0212904)
Supplement: S1 Source Code — This includes all custom code used in the described system and related evaluations. (ZIP) [file pone.0212904.s009.zip › robotic_body_surrogates_source_code/hrl-haptic-manip-pkgs.zip!/hrl-haptic-manip-pkgs/hrl_haptic_mpc/docs/html/classhrl__haptic__mpc_1_1robot__haptic__state__node_1_1_robot_haptic_state_server.html]

HRL Haptic MPC: hrl\_haptic\_mpc.robot\_haptic\_state\_node.RobotHapticStateServer Class Reference


HRL Haptic MPC

A haptic model predictive controller

- Main Page
- Packages
- Classes

- Class List
- Class Index
- Class Members

All Classes Namespaces Functions Variables

- hrl\_haptic\_mpc
- **robot\_haptic\_state\_node**
- RobotHapticStateServer

Public Member Functions |
Public Attributes

hrl\_haptic\_mpc.robot\_haptic\_state\_node.RobotHapticStateServer Class Reference

publisher: publishes all relevant haptic state information on a common interface independent of robot type.
More...

List of all members.

|  |  |
| --- | --- |
| Public Member Functions | |
| def | \_\_init\_\_ |
|  | Constructor for robot haptic state server. |
| def | initRobot |
|  | Initialise all robot specific parameters (skin topics, robot interfaces, etc). |
| def | initPR2 |
|  | Initialise parameters for the state publisher when used on the PR2. |
| def | initCody |
|  | Initialise parameters for the state publisher when used on Cody. |
| def | initSim |
|  | Initialise parameters for the state publisher when used in simulation with the 3DOF arm. |
| def | **initSimCody** |
| def | **initComms** |
| def | **getMessageHeader** |
| def | **updateEndEffectorJacobian** |
| def | updateContactJacobians |
|  | Compute contact Jacobians based on the provided taxel array dictionary. |
| def | updateTorsoPose |
|  | Returns a Pose object for the torso pose in the stated inertial frame. |
| def | **updateJointStates** |
| def | **updateEndEffectorPose** |
| def | modifyPR2Taxels |
|  | Modify taxel data for PR2 specific situations TODO: Survy to implement selective taxel ignoring. |
| def | modifyRobotSpecificTaxels |
|  | Modifies data from the taxel array based on robot specific configurations. |
| def | publishRobotState |
|  | Build and publish the haptic state message. |
| def | start |
|  | Start the state publisher. |
| Public Attributes | |
|  | **opt** |
|  | **node\_name** |
|  | **tf\_listener** |
|  | **state\_pub** |
|  | **rate** |
|  | **msg\_seq** |
|  | **base\_path** |
|  | **skin\_topic\_list** |
|  | **skin\_client** |
|  | **robot** |
|  | joint\_names |
|  | Store latest joint states from the specified robot class. |
|  | **joint\_angles** |
|  | **desired\_joint\_angles** |
|  | **joint\_velocities** |
|  | **joint\_stiffness** |
|  | **joint\_damping** |
|  | **joint\_data\_lock** |
|  | **end\_effector\_position** |
|  | **end\_effector\_orient\_cart** |
|  | **end\_effector\_orient\_quat** |
|  | **Jc** |
|  | **Je** |
|  | **trim\_threshold** |
|  | **ma\_to\_m** |
|  | **robot\_path** |
|  | **torso\_frame** |
|  | **inertial\_frame** |
|  | **joint\_limits\_max** |
|  | **joint\_limits\_min** |
|  | **gripper\_pose\_pub** |

---

## Detailed Description

publisher: publishes all relevant haptic state information on a common interface independent of robot type.

---

## Member Function Documentation

|  |  |  |  |  |  |
| --- | --- | --- | --- | --- | --- |
| def hrl\_haptic\_mpc.robot\_haptic\_state\_node.RobotHapticStateServer.initCody | ( |  | *self* | ) |  |

Initialise parameters for the state publisher when used on Cody.

|  |  |  |  |  |  |
| --- | --- | --- | --- | --- | --- |
| def hrl\_haptic\_mpc.robot\_haptic\_state\_node.RobotHapticStateServer.initPR2 | ( |  | *self* | ) |  |

Initialise parameters for the state publisher when used on the PR2.

|  |  |  |  |
| --- | --- | --- | --- |
| def hrl\_haptic\_mpc.robot\_haptic\_state\_node.RobotHapticStateServer.initRobot | ( |  | *self*, |
|  |  |  | *robot\_type* = `"pr2"` |
|  | ) |  |  |

Initialise all robot specific parameters (skin topics, robot interfaces, etc).

Calls the appropriate init function.

|  |  |  |  |  |  |
| --- | --- | --- | --- | --- | --- |
| def hrl\_haptic\_mpc.robot\_haptic\_state\_node.RobotHapticStateServer.initSim | ( |  | *self* | ) |  |

Initialise parameters for the state publisher when used in simulation with the 3DOF arm.

|  |  |  |  |
| --- | --- | --- | --- |
| def hrl\_haptic\_mpc.robot\_haptic\_state\_node.RobotHapticStateServer.modifyPR2Taxels | ( |  | *self*, |
|  |  |  | *skin\_data* |
|  | ) |  |  |

Modify taxel data for PR2 specific situations TODO: Survy to implement selective taxel ignoring.

**Parameters:**
:   |  |  |
    | --- | --- |
    | skin\_data | Dictionary containing taxel array messages indexed by topic name |

|  |  |  |  |
| --- | --- | --- | --- |
| def hrl\_haptic\_mpc.robot\_haptic\_state\_node.RobotHapticStateServer.modifyRobotSpecificTaxels | ( |  | *self*, |
|  |  |  | *skin\_data* |
|  | ) |  |  |

Modifies data from the taxel array based on robot specific configurations.

An example of this is ignoring the PR2 wrist taxels when the wrist is near its joint limit as the wrist itself will trigger the taxel.

**Parameters:**
:   |  |  |
    | --- | --- |
    | skin\_data | Dict containing taxel array messages indexed by topic name |

**Returns:**
:   skin\_data Modified dictionary containing taxel array messages

|  |  |  |  |  |  |
| --- | --- | --- | --- | --- | --- |
| def hrl\_haptic\_mpc.robot\_haptic\_state\_node.RobotHapticStateServer.publishRobotState | ( |  | *self* | ) |  |

Build and publish the haptic state message.

|  |  |  |  |
| --- | --- | --- | --- |
| def hrl\_haptic\_mpc.robot\_haptic\_state\_node.RobotHapticStateServer.updateContactJacobians | ( |  | *self*, |
|  |  |  | *skin\_data* |
|  | ) |  |  |

Compute contact Jacobians based on the provided taxel array dictionary.

**Parameters:**
:   |  |  |
    | --- | --- |
    | skin\_data | Dictionary containing taxel array messages indexed by topic name |

---

The documentation for this class was generated from the following file:

- /home/jdh/ros\_sandbox/hrl\_haptic\_manipulation\_in\_clutter/hrl\_haptic\_mpc/src/hrl\_haptic\_mpc/robot\_haptic\_state\_node.py


All Classes Namespaces Functions Variables

---

Generated on Thu Jan 17 2013 16:21:14 for HRL Haptic MPC by  

 1.7.6.1
