# Supplementary material for: In-home and remote use of robotic body surrogates by people with profound motor deficits
Source: PLoS One. 2019 Mar 15;14(3):e0212904. doi: 10.1371/journal.pone.0212904 (PMC6420017; doi:10.1371/journal.pone.0212904)
Supplement: S1 Source Code — This includes all custom code used in the described system and related evaluations. (ZIP) [file pone.0212904.s009.zip › robotic_body_surrogates_source_code/hrl-haptic-manip-pkgs.zip!/hrl-haptic-manip-pkgs/hrl_haptic_mpc/docs/html/classhrl__haptic__mpc_1_1skin__client_1_1_taxel_array_client.html]

HRL Haptic MPC: hrl\_haptic\_mpc.skin\_client.TaxelArrayClient Class Reference


HRL Haptic MPC

A haptic model predictive controller

- Main Page
- Packages
- Classes

- Class List
- Class Index
- Class Members

All Classes Namespaces Functions Variables

- hrl\_haptic\_mpc
- **skin\_client**
- TaxelArrayClient

Public Member Functions |
Public Attributes

hrl\_haptic\_mpc.skin\_client.TaxelArrayClient Class Reference

Provides an interface to process TaxelArrays published on multiple topics, including trimming the data and transforming the reference frame.
More...

List of all members.

|  |  |
| --- | --- |
| Public Member Functions | |
| def | \_\_init\_\_ |
|  | Constructor. |
| def | setTrimThreshold |
|  | Set the trim threshold used by the client. |
| def | addSkinTopicCallback |
|  | Callback function which sets the topic. |
| def | removeSkinTopicCallback |
|  | Callback function to removed skin topic. |
| def | addSkinTopic |
|  | Add skin topic to internal data structures. |
| def | removeSkinTopic |
|  | Remove skin topic from internal data structures. |
| def | skinCallback |
|  | Skin Callback. |
| def | transformTaxelArray |
|  | Transform a single taxel array message from one frame to another. |
| def | trimSkinContacts |
|  | Return a trimmed copy of the the skin\_data dictionary. |
| def | trimTaxelArray |
|  | Trim a passed TaxelArray to only incorporate forces of significance. |
| def | getSkinData |
|  | getSkinData accessor function Returns a copy of the skin\_data dictionary |
| def | getTrimmedSkinData |
|  | getTrimmedSkinData accessor function Returns a copy of the trimmed\_skin\_data dictionary |
| def | **getContactLocationsFromTaxelArray** |
| def | getTaxelLocationAndJointList |
|  | Returns a list of taxel locations and list of joint numbers after which the joint torque will have no effect on the contact force, and optionally a time stamp Must be implemented by every robot specific skin client. |
| Public Attributes | |
|  | data\_lock |
|  | Lock for skin data structure. |
|  | topic\_lock |
|  | Lock for skin data topics. |
|  | torso\_frame |
|  | torso\_frame Torso frame ID used as the base frame for the associated arm, eg, "/torso\_lift\_link" |
|  | trim\_threshold |
|  | Threshold used to control how the data is trimmed - should be set by whatever instatiates a TaxelArrayClient. |
|  | skin\_subs |
|  | Dictionary containing the skin subscribers, indexed by topic name. |
|  | skin\_data |
|  | Dictionary containing the raw TaxelArray messages heard by the client, indexed by topic name. |
|  | trimmed\_skin\_data |
|  | Dictionary containing the processed TaxelArray messages heard by the client, indexed by topic name. |
|  | tf\_lstnr |
|  | TF Listener. |
|  | skin\_topic\_list |
|  | List of skin topics used by the client. |
|  | current\_topics\_pub |
|  | Add skin topic subscriber. |

---

## Detailed Description

Provides an interface to process TaxelArrays published on multiple topics, including trimming the data and transforming the reference frame.

---

## Constructor & Destructor Documentation

|  |  |  |  |
| --- | --- | --- | --- |
| def hrl\_haptic\_mpc.skin\_client.TaxelArrayClient.\_\_init\_\_ | ( |  | *self*, |
|  |  |  | *skin\_topic\_list*, |
|  |  |  | *torso\_frame* = `"/torso_lift_link"`, |
|  |  |  | *tf\_listener* = `None` |
|  | ) |  |  |

Constructor.

**Parameters:**
:   |  |  |
    | --- | --- |
    | skin\_topic\_list | List of strings specifying the topics to subscribe to for TaxelArray messages |
    | torso\_frame | String indicating which frame ID is the base frame for the arm |
    | tf\_listener | TF Listener object, if one exists. If this is None, the class will create one. |

---

## Member Function Documentation

|  |  |  |  |
| --- | --- | --- | --- |
| def hrl\_haptic\_mpc.skin\_client.TaxelArrayClient.addSkinTopic | ( |  | *self*, |
|  |  |  | *skin\_topic* |
|  | ) |  |  |

Add skin topic to internal data structures.

**Parameters:**
:   |  |  |
    | --- | --- |
    | skin\_topic | String specifying the topic to be added. |

|  |  |  |  |
| --- | --- | --- | --- |
| def hrl\_haptic\_mpc.skin\_client.TaxelArrayClient.addSkinTopicCallback | ( |  | *self*, |
|  |  |  | *msg* |
|  | ) |  |  |

Callback function which sets the topic.

**Parameters:**
:   |  |  |
    | --- | --- |
    | msg | std\_msgs/String message. |

|  |  |  |  |  |  |
| --- | --- | --- | --- | --- | --- |
| def hrl\_haptic\_mpc.skin\_client.TaxelArrayClient.getTaxelLocationAndJointList | ( |  | *self* | ) |  |

Returns a list of taxel locations and list of joint numbers after which the joint torque will have no effect on the contact force, and optionally a time stamp Must be implemented by every robot specific skin client.

|  |  |  |  |
| --- | --- | --- | --- |
| def hrl\_haptic\_mpc.skin\_client.TaxelArrayClient.removeSkinTopic | ( |  | *self*, |
|  |  |  | *skin\_topic* |
|  | ) |  |  |

Remove skin topic from internal data structures.

**Parameters:**
:   |  |  |
    | --- | --- |
    | skin\_topic | String specifying the topic to be removed. |

|  |  |  |  |
| --- | --- | --- | --- |
| def hrl\_haptic\_mpc.skin\_client.TaxelArrayClient.removeSkinTopicCallback | ( |  | *self*, |
|  |  |  | *msg* |
|  | ) |  |  |

Callback function to removed skin topic.

**Parameters:**
:   |  |  |
    | --- | --- |
    | msg | StringArray message. |

|  |  |  |  |
| --- | --- | --- | --- |
| def hrl\_haptic\_mpc.skin\_client.TaxelArrayClient.setTrimThreshold | ( |  | *self*, |
|  |  |  | *threshold* |
|  | ) |  |  |

Set the trim threshold used by the client.

Should be greater or equal to 0.0.

**Parameters:**
:   |  |  |
    | --- | --- |
    | threshold | Desired threshold. Should be greater than or equal to 0.0. |

|  |  |  |  |
| --- | --- | --- | --- |
| def hrl\_haptic\_mpc.skin\_client.TaxelArrayClient.skinCallback | ( |  | *self*, |
|  |  |  | *msg*, |
|  |  |  | *skin\_topic* |
|  | ) |  |  |

Skin Callback.

Store the message in the data dictionary, indexed by topic. Keeps the raw data in dictionary 'skin\_data' and the transformed, trimmed data in 'trimmed\_skin\_data'

**Parameters:**
:   |  |  |
    | --- | --- |
    | msg | TaxelArray message object |
    | skin\_topic | The topic name triggering the callback. Used to identify what sensor the TaxelArray came from (as there may be multiple publishers running) |

|  |  |  |  |
| --- | --- | --- | --- |
| def hrl\_haptic\_mpc.skin\_client.TaxelArrayClient.transformTaxelArray | ( |  | *self*, |
|  |  |  | *ta\_msg*, |
|  |  |  | *new\_frame* |
|  | ) |  |  |

Transform a single taxel array message from one frame to another.

**Parameters:**
:   |  |  |
    | --- | --- |
    | ta\_msg | TaxelArray message object to be transformed |
    | new\_frame | The desired frame name |

**Returns:**
:   The transformed message with all values in the new coordinate frame.

|  |  |  |  |
| --- | --- | --- | --- |
| def hrl\_haptic\_mpc.skin\_client.TaxelArrayClient.trimSkinContacts | ( |  | *self*, |
|  |  |  | *threshold* |
|  | ) |  |  |

Return a trimmed copy of the the skin\_data dictionary.

Each TaxelArray within the structure will be trimmed.

**Parameters:**
:   |  |  |
    | --- | --- |
    | threshold | Threshold parameter (float greater than 0.0) |

|  |  |  |  |
| --- | --- | --- | --- |
| def hrl\_haptic\_mpc.skin\_client.TaxelArrayClient.trimTaxelArray | ( |  | *self*, |
|  |  |  | *ta\_msg*, |
|  |  |  | *threshold* |
|  | ) |  |  |

Trim a passed TaxelArray to only incorporate forces of significance.

Returns a trimmed TaxelArray message object with forces of magnitude > threshold. The data is otherwise unchanged.

---

## Member Data Documentation

hrl\_haptic\_mpc::skin\_client.TaxelArrayClient::current\_topics\_pub

Add skin topic subscriber.

Remove skin topic subscriber Current topics ROS publisher. Publishes the list of topics on change.

hrl\_haptic\_mpc::skin\_client.TaxelArrayClient::data\_lock

Lock for skin data structure.

hrl\_haptic\_mpc::skin\_client.TaxelArrayClient::topic\_lock

Lock for skin data topics.

hrl\_haptic\_mpc::skin\_client.TaxelArrayClient::trim\_threshold

Threshold used to control how the data is trimmed - should be set by whatever instatiates a TaxelArrayClient.

Default: 0.0 (ie, trim nothing)

---

The documentation for this class was generated from the following file:

- /home/jdh/ros\_sandbox/hrl\_haptic\_manipulation\_in\_clutter/hrl\_haptic\_mpc/src/hrl\_haptic\_mpc/skin\_client.py


All Classes Namespaces Functions Variables

---

Generated on Thu Jan 17 2013 16:21:14 for HRL Haptic MPC by  

 1.7.6.1
