# Supplementary material for: In-home and remote use of robotic body surrogates by people with profound motor deficits
Source: PLoS One. 2019 Mar 15;14(3):e0212904. doi: 10.1371/journal.pone.0212904 (PMC6420017; doi:10.1371/journal.pone.0212904)
Supplement: S1 Source Code — This includes all custom code used in the described system and related evaluations. (ZIP) [file pone.0212904.s009.zip › robotic_body_surrogates_source_code/hrl-haptic-manip-pkgs.zip!/hrl-haptic-manip-pkgs/hrl_haptic_mpc/docs/html/classhrl__haptic__mpc_1_1waypoint__generator_1_1_waypoint_generator-members.html]

HRL Haptic MPC: Member List


HRL Haptic MPC

A haptic model predictive controller

- Main Page
- Packages
- Classes

- Class List
- Class Index
- Class Members

- hrl\_haptic\_mpc
- **waypoint\_generator**
- WaypointGenerator

hrl\_haptic\_mpc.waypoint\_generator.WaypointGenerator Member List

This is the complete list of members for hrl\_haptic\_mpc.waypoint\_generator.WaypointGenerator, including all inherited members.

|  |  |  |
| --- | --- | --- |
| \_\_init\_\_ | hrl\_haptic\_mpc.waypoint\_generator.WaypointGenerator |  |
| angularDistanceBetweenPoses | hrl\_haptic\_mpc.waypoint\_generator.WaypointGenerator |  |
| **at\_waypoint\_ang\_threshold** (defined in hrl\_haptic\_mpc.waypoint\_generator.WaypointGenerator) | hrl\_haptic\_mpc.waypoint\_generator.WaypointGenerator | `[static]` |
| **at\_waypoint\_threshold** (defined in hrl\_haptic\_mpc.waypoint\_generator.WaypointGenerator) | hrl\_haptic\_mpc.waypoint\_generator.WaypointGenerator | `[static]` |
| **base\_path** (defined in hrl\_haptic\_mpc.waypoint\_generator.WaypointGenerator) | hrl\_haptic\_mpc.waypoint\_generator.WaypointGenerator | `[static]` |
| **cody\_param\_path** (defined in hrl\_haptic\_mpc.waypoint\_generator.WaypointGenerator) | hrl\_haptic\_mpc.waypoint\_generator.WaypointGenerator | `[static]` |
| **current\_gripper\_waypoint** (defined in hrl\_haptic\_mpc.waypoint\_generator.WaypointGenerator) | hrl\_haptic\_mpc.waypoint\_generator.WaypointGenerator | `[static]` |
| **current\_pose\_topic** (defined in hrl\_haptic\_mpc.waypoint\_generator.WaypointGenerator) | hrl\_haptic\_mpc.waypoint\_generator.WaypointGenerator | `[static]` |
| **current\_trajectory\_deque** (defined in hrl\_haptic\_mpc.waypoint\_generator.WaypointGenerator) | hrl\_haptic\_mpc.waypoint\_generator.WaypointGenerator | `[static]` |
| **current\_trajectory\_msg** (defined in hrl\_haptic\_mpc.waypoint\_generator.WaypointGenerator) | hrl\_haptic\_mpc.waypoint\_generator.WaypointGenerator |  |
| **default\_eq\_gen\_type** (defined in hrl\_haptic\_mpc.waypoint\_generator.WaypointGenerator) | hrl\_haptic\_mpc.waypoint\_generator.WaypointGenerator | `[static]` |
| distanceBetweenPoses | hrl\_haptic\_mpc.waypoint\_generator.WaypointGenerator |  |
| **epcon** (defined in hrl\_haptic\_mpc.waypoint\_generator.WaypointGenerator) | hrl\_haptic\_mpc.waypoint\_generator.WaypointGenerator | `[static]` |
| **eq\_gen\_type** (defined in hrl\_haptic\_mpc.waypoint\_generator.WaypointGenerator) | hrl\_haptic\_mpc.waypoint\_generator.WaypointGenerator | `[static]` |
| generateWaypoint | hrl\_haptic\_mpc.waypoint\_generator.WaypointGenerator |  |
| getMessageHeader | hrl\_haptic\_mpc.waypoint\_generator.WaypointGenerator |  |
| getOrientationStep | hrl\_haptic\_mpc.waypoint\_generator.WaypointGenerator |  |
| getPositionStep | hrl\_haptic\_mpc.waypoint\_generator.WaypointGenerator |  |
| getWaypointFromTrajectory | hrl\_haptic\_mpc.waypoint\_generator.WaypointGenerator |  |
| **goal\_lock** (defined in hrl\_haptic\_mpc.waypoint\_generator.WaypointGenerator) | hrl\_haptic\_mpc.waypoint\_generator.WaypointGenerator | `[static]` |
| **goal\_pose** (defined in hrl\_haptic\_mpc.waypoint\_generator.WaypointGenerator) | hrl\_haptic\_mpc.waypoint\_generator.WaypointGenerator | `[static]` |
| **goal\_pose\_array\_topic** (defined in hrl\_haptic\_mpc.waypoint\_generator.WaypointGenerator) | hrl\_haptic\_mpc.waypoint\_generator.WaypointGenerator | `[static]` |
| **goal\_pose\_topic** (defined in hrl\_haptic\_mpc.waypoint\_generator.WaypointGenerator) | hrl\_haptic\_mpc.waypoint\_generator.WaypointGenerator | `[static]` |
| goalPoseCallback | hrl\_haptic\_mpc.waypoint\_generator.WaypointGenerator |  |
| **gripper\_pose** (defined in hrl\_haptic\_mpc.waypoint\_generator.WaypointGenerator) | hrl\_haptic\_mpc.waypoint\_generator.WaypointGenerator | `[static]` |
| initCody | hrl\_haptic\_mpc.waypoint\_generator.WaypointGenerator |  |
| initComms | hrl\_haptic\_mpc.waypoint\_generator.WaypointGenerator |  |
| initPR2 | hrl\_haptic\_mpc.waypoint\_generator.WaypointGenerator |  |
| initSim3 | hrl\_haptic\_mpc.waypoint\_generator.WaypointGenerator |  |
| **joint\_angles** (defined in hrl\_haptic\_mpc.waypoint\_generator.WaypointGenerator) | hrl\_haptic\_mpc.waypoint\_generator.WaypointGenerator |  |
| jointTrajectoryCallback | hrl\_haptic\_mpc.waypoint\_generator.WaypointGenerator |  |
| **max\_ang\_step** (defined in hrl\_haptic\_mpc.waypoint\_generator.WaypointGenerator) | hrl\_haptic\_mpc.waypoint\_generator.WaypointGenerator | `[static]` |
| **max\_pos\_step** (defined in hrl\_haptic\_mpc.waypoint\_generator.WaypointGenerator) | hrl\_haptic\_mpc.waypoint\_generator.WaypointGenerator | `[static]` |
| **msg\_seq** (defined in hrl\_haptic\_mpc.waypoint\_generator.WaypointGenerator) | hrl\_haptic\_mpc.waypoint\_generator.WaypointGenerator | `[static]` |
| **node\_name** (defined in hrl\_haptic\_mpc.waypoint\_generator.WaypointGenerator) | hrl\_haptic\_mpc.waypoint\_generator.WaypointGenerator | `[static]` |
| **opt** (defined in hrl\_haptic\_mpc.waypoint\_generator.WaypointGenerator) | hrl\_haptic\_mpc.waypoint\_generator.WaypointGenerator | `[static]` |
| **param\_path** (defined in hrl\_haptic\_mpc.waypoint\_generator.WaypointGenerator) | hrl\_haptic\_mpc.waypoint\_generator.WaypointGenerator | `[static]` |
| **pose\_traj\_viz\_pub** (defined in hrl\_haptic\_mpc.waypoint\_generator.WaypointGenerator) | hrl\_haptic\_mpc.waypoint\_generator.WaypointGenerator |  |
| **pose\_waypoint\_pub** (defined in hrl\_haptic\_mpc.waypoint\_generator.WaypointGenerator) | hrl\_haptic\_mpc.waypoint\_generator.WaypointGenerator |  |
| poseTrajectoryCallback | hrl\_haptic\_mpc.waypoint\_generator.WaypointGenerator |  |
| **pr2\_param\_path** (defined in hrl\_haptic\_mpc.waypoint\_generator.WaypointGenerator) | hrl\_haptic\_mpc.waypoint\_generator.WaypointGenerator | `[static]` |
| **rate** (defined in hrl\_haptic\_mpc.waypoint\_generator.WaypointGenerator) | hrl\_haptic\_mpc.waypoint\_generator.WaypointGenerator | `[static]` |
| **robot** (defined in hrl\_haptic\_mpc.waypoint\_generator.WaypointGenerator) | hrl\_haptic\_mpc.waypoint\_generator.WaypointGenerator | `[static]` |
| **robot\_kinematics** (defined in hrl\_haptic\_mpc.waypoint\_generator.WaypointGenerator) | hrl\_haptic\_mpc.waypoint\_generator.WaypointGenerator |  |
| **robot\_name** (defined in hrl\_haptic\_mpc.waypoint\_generator.WaypointGenerator) | hrl\_haptic\_mpc.waypoint\_generator.WaypointGenerator | `[static]` |
| robotStateCallback | hrl\_haptic\_mpc.waypoint\_generator.WaypointGenerator |  |
| **scl** (defined in hrl\_haptic\_mpc.waypoint\_generator.WaypointGenerator) | hrl\_haptic\_mpc.waypoint\_generator.WaypointGenerator | `[static]` |
| **sensor** (defined in hrl\_haptic\_mpc.waypoint\_generator.WaypointGenerator) | hrl\_haptic\_mpc.waypoint\_generator.WaypointGenerator | `[static]` |
| **sim\_param\_path** (defined in hrl\_haptic\_mpc.waypoint\_generator.WaypointGenerator) | hrl\_haptic\_mpc.waypoint\_generator.WaypointGenerator | `[static]` |
| **simcody\_param\_path** (defined in hrl\_haptic\_mpc.waypoint\_generator.WaypointGenerator) | hrl\_haptic\_mpc.waypoint\_generator.WaypointGenerator | `[static]` |
| **skin\_topic\_list** (defined in hrl\_haptic\_mpc.waypoint\_generator.WaypointGenerator) | hrl\_haptic\_mpc.waypoint\_generator.WaypointGenerator | `[static]` |
| start | hrl\_haptic\_mpc.waypoint\_generator.WaypointGenerator |  |
| **state\_lock** (defined in hrl\_haptic\_mpc.waypoint\_generator.WaypointGenerator) | hrl\_haptic\_mpc.waypoint\_generator.WaypointGenerator | `[static]` |
| straightLineTrajectory | hrl\_haptic\_mpc.waypoint\_generator.WaypointGenerator |  |
| **tf\_listener** (defined in hrl\_haptic\_mpc.waypoint\_generator.WaypointGenerator) | hrl\_haptic\_mpc.waypoint\_generator.WaypointGenerator |  |
| **traj\_lock** (defined in hrl\_haptic\_mpc.waypoint\_generator.WaypointGenerator) | hrl\_haptic\_mpc.waypoint\_generator.WaypointGenerator | `[static]` |
| **waypoint\_pose\_topic** (defined in hrl\_haptic\_mpc.waypoint\_generator.WaypointGenerator) | hrl\_haptic\_mpc.waypoint\_generator.WaypointGenerator | `[static]` |


All Classes Namespaces Functions Variables

---

Generated on Thu Jan 17 2013 16:21:14 for HRL Haptic MPC by  

 1.7.6.1
