# Supplementary material for: In-home and remote use of robotic body surrogates by people with profound motor deficits
Source: PLoS One. 2019 Mar 15;14(3):e0212904. doi: 10.1371/journal.pone.0212904 (PMC6420017; doi:10.1371/journal.pone.0212904)
Supplement: S1 Source Code — This includes all custom code used in the described system and related evaluations. (ZIP) [file pone.0212904.s009.zip › robotic_body_surrogates_source_code/hrl-haptic-manip-pkgs.zip!/hrl-haptic-manip-pkgs/hrl_haptic_mpc/docs/html/classhrl__haptic__mpc_1_1waypoint__generator_1_1_waypoint_generator.html]

HRL Haptic MPC: hrl\_haptic\_mpc.waypoint\_generator.WaypointGenerator Class Reference


HRL Haptic MPC

A haptic model predictive controller

- Main Page
- Packages
- Classes

- Class List
- Class Index
- Class Members

All Classes Namespaces Functions Variables

- hrl\_haptic\_mpc
- **waypoint\_generator**
- WaypointGenerator

Public Member Functions |
Public Attributes |
Static Public Attributes

hrl\_haptic\_mpc.waypoint\_generator.WaypointGenerator Class Reference

takes either a goal pose or a trajectory and passes local goals to the MPC controller to try to make the controller follow it.
More...

List of all members.

|  |  |
| --- | --- |
| Public Member Functions | |
| def | \_\_init\_\_ |
|  | Constructor. |
| def | initCody |
|  | Initialise 3DOF Sim kinematics. |
| def | initPR2 |
|  | Initialise PR2 kinematics. |
| def | initSim3 |
|  | Initialise 3DOF Sim kinematics. |
| def | initComms |
|  | Initialise all publishers/subscribers used by the waypoint generator. |
| def | goalPoseCallback |
|  | Update goal pose. |
| def | robotStateCallback |
|  | Store the current pose from the haptic state publisher. |
| def | poseTrajectoryCallback |
|  | Store a trajectory of poses in the deque. |
| def | jointTrajectoryCallback |
|  | Store a joint angle trajectory in the deque. |
| def | straightLineTrajectory |
|  | Returns the next waypoint along a straight line trajectory from the current gripper pose to the goal pose. |
| def | getPositionStep |
|  | Returns a linearly interpolated step towards the goal pos from the current pos. |
| def | getOrientationStep |
|  | Returns a linearly interpolated step towards the goal orientation from the current orientation (using SLERP) |
| def | getMessageHeader |
|  | Returns a message header (std\_msgs object), populated with the time, frame\_id and sequence number. |
| def | distanceBetweenPoses |
|  | Euclidian distance between two poses. |
| def | angularDistanceBetweenPoses |
|  | Return the angle between two quaternions (axis-angle representation) |
| def | getWaypointFromTrajectory |
|  | Try to get a waypoint pose from the trajectory deque. |
| def | generateWaypoint |
|  | Publishes the next waypoint for the control to try to achieve. |
| def | start |
|  | Start the waypoint generator publishing waypoints. |
| Public Attributes | |
|  | **robot\_kinematics** |
|  | **tf\_listener** |
|  | **pose\_waypoint\_pub** |
|  | **pose\_traj\_viz\_pub** |
|  | **joint\_angles** |
|  | **current\_trajectory\_msg** |
| Static Public Attributes | |
|  | **node\_name** = None |
|  | **opt** = None |
| float | **rate** = 25.0 |
| int | **msg\_seq** = 0 |
| string | **param\_path** = "" |
| string | **base\_path** = "/haptic\_mpc" |
| string | **cody\_param\_path** = "/cody" |
| string | **pr2\_param\_path** = "/pr2" |
| string | **sim\_param\_path** = "/sim3" |
| string | **simcody\_param\_path** = "/simcody" |
| string | **current\_pose\_topic** = "/haptic\_mpc/robot\_state" |
| string | **goal\_pose\_topic** = "/haptic\_mpc/goal\_pose" |
| string | **goal\_pose\_array\_topic** = "/haptic\_mpc/goal\_pose\_array" |
| string | **waypoint\_pose\_topic** = "/haptic\_mpc/traj\_pose" |
|  | **robot** = None |
|  | **robot\_name** = None |
|  | **sensor** = None |
|  | **eq\_gen\_type** = None |
| string | **default\_eq\_gen\_type** = 'mpc\_qs\_1' |
|  | **skin\_topic\_list** = None |
|  | **scl** = None |
|  | **epcon** = None |
| float | **max\_pos\_step** = 0.05 |
| float | **max\_ang\_step** = 0.02 |
| float | **at\_waypoint\_threshold** = 0.02 |
| tuple | **at\_waypoint\_ang\_threshold** = numpy.radians(5.0) |
| tuple | **current\_trajectory\_deque** = collections.deque() |
|  | **gripper\_pose** = None |
|  | **goal\_pose** = None |
|  | **current\_gripper\_waypoint** = None |
| tuple | **traj\_lock** = threading.RLock() |
| tuple | **goal\_lock** = threading.RLock() |
| tuple | **state\_lock** = threading.RLock() |

---

## Detailed Description

takes either a goal pose or a trajectory and passes local goals to the MPC controller to try to make the controller follow it.

---

## Constructor & Destructor Documentation

|  |  |  |  |
| --- | --- | --- | --- |
| def hrl\_haptic\_mpc.waypoint\_generator.WaypointGenerator.\_\_init\_\_ | ( |  | *self*, |
|  |  |  | *node\_name*, |
|  |  |  | *opt* |
|  | ) |  |  |

Constructor.

Calls functions to initialise robot specific parameters, then initialises all publishers/subscribers.

---

## Member Function Documentation

|  |  |  |  |
| --- | --- | --- | --- |
| def hrl\_haptic\_mpc.waypoint\_generator.WaypointGenerator.angularDistanceBetweenPoses | ( |  | *self*, |
|  |  |  | *poseA*, |
|  |  |  | *poseB* |
|  | ) |  |  |

Return the angle between two quaternions (axis-angle representation)

**Parameters:**
:   |  |  |
    | --- | --- |
    | poseA | geometry\_msgs.msg.Pose |
    | poseB | geometry\_msgs.msg.Pose |

**Returns:**
:   The angular distance between the two pose orientations.

|  |  |  |  |
| --- | --- | --- | --- |
| def hrl\_haptic\_mpc.waypoint\_generator.WaypointGenerator.distanceBetweenPoses | ( |  | *self*, |
|  |  |  | *poseA*, |
|  |  |  | *poseB* |
|  | ) |  |  |

Euclidian distance between two poses.

Ignores differences in orientation.

**Parameters:**
:   |  |  |
    | --- | --- |
    | poseA | geometry\_msgs.msg.Pose |
    | poseB | geometry\_msgs.msg.Pose |

**Returns:**
:   The euclidian distance between the two pose positions.

|  |  |  |  |  |  |
| --- | --- | --- | --- | --- | --- |
| def hrl\_haptic\_mpc.waypoint\_generator.WaypointGenerator.generateWaypoint | ( |  | *self* | ) |  |

Publishes the next waypoint for the control to try to achieve.

If we have a valid trajectory, get the next waypoint from that. If not and we have a valid end goal pose instead, move in a straight line towards it. If we have neither, don't publish anything.

|  |  |  |  |
| --- | --- | --- | --- |
| def hrl\_haptic\_mpc.waypoint\_generator.WaypointGenerator.getOrientationStep | ( |  | *self*, |
|  |  |  | *q\_h\_orient*, |
|  |  |  | *q\_g\_orient*, |
|  |  |  | *max\_ang\_step* |
|  | ) |  |  |

Returns a linearly interpolated step towards the goal orientation from the current orientation (using SLERP)

**Parameters:**
:   |  |  |
    | --- | --- |
    | q\_h\_orient | Current hand orientation as a quaternion in numpy array form (assumed to be [x,y,z,w]) |
    | q\_g\_orient | Current goal orientation as a quaternion in numpy array form (assumed to be [x,y,z,w]) |
    | max\_ang\_step | A scalar max step size for orientation (in radians). |

**Returns:**
:   An interpolated step in orientation towards the goal.

|  |  |  |  |
| --- | --- | --- | --- |
| def hrl\_haptic\_mpc.waypoint\_generator.WaypointGenerator.getPositionStep | ( |  | *self*, |
|  |  |  | *current\_pos*, |
|  |  |  | *goal\_pos*, |
|  |  |  | *max\_pos\_step* |
|  | ) |  |  |

Returns a linearly interpolated step towards the goal pos from the current pos.

**Parameters:**
:   |  |  |
    | --- | --- |
    | current\_pos | Current position as a numpy array (assumed to be [x,y,z]) |
    | goal\_pos | Goal position as a numpy array (assumed to be [x,y,z]) |
    | max\_pos\_step | A scalar max step size for position (in metres). |

**Returns:**
:   An interpolated step in position towards the goal.

|  |  |  |  |  |  |
| --- | --- | --- | --- | --- | --- |
| def hrl\_haptic\_mpc.waypoint\_generator.WaypointGenerator.getWaypointFromTrajectory | ( |  | *self* | ) |  |

Try to get a waypoint pose from the trajectory deque.

Also trims the trajectory to reduce the number of waypoint. Scans through the trajectory to find the next pose that is at least max\_pos\_step away (or the last pose in the deque).

**Returns:**
:   A Pose object if there is a currently stored trajectory, otherwise None.

|  |  |  |  |
| --- | --- | --- | --- |
| def hrl\_haptic\_mpc.waypoint\_generator.WaypointGenerator.goalPoseCallback | ( |  | *self*, |
|  |  |  | *msg* |
|  | ) |  |  |

Update goal pose.

**Parameters:**
:   |  |  |
    | --- | --- |
    | msg | A geometry\_msgs.msg.PoseStamped object. |

|  |  |  |  |  |  |
| --- | --- | --- | --- | --- | --- |
| def hrl\_haptic\_mpc.waypoint\_generator.WaypointGenerator.initCody | ( |  | *self* | ) |  |

Initialise 3DOF Sim kinematics.

NB: This doesn't actually do anything and is added mostly for structural consistency.

|  |  |  |  |  |  |
| --- | --- | --- | --- | --- | --- |
| def hrl\_haptic\_mpc.waypoint\_generator.WaypointGenerator.initComms | ( |  | *self* | ) |  |

Initialise all publishers/subscribers used by the waypoint generator.

|  |  |  |  |  |  |
| --- | --- | --- | --- | --- | --- |
| def hrl\_haptic\_mpc.waypoint\_generator.WaypointGenerator.initPR2 | ( |  | *self* | ) |  |

Initialise PR2 kinematics.

NB: Only used for joint limits, will eventually be removed once these are passed with the robot state.

|  |  |  |  |  |  |
| --- | --- | --- | --- | --- | --- |
| def hrl\_haptic\_mpc.waypoint\_generator.WaypointGenerator.initSim3 | ( |  | *self* | ) |  |

Initialise 3DOF Sim kinematics.

NB: This doesn't actually do anything and is added mostly for structural consistency.

|  |  |  |  |
| --- | --- | --- | --- |
| def hrl\_haptic\_mpc.waypoint\_generator.WaypointGenerator.jointTrajectoryCallback | ( |  | *self*, |
|  |  |  | *msg* |
|  | ) |  |  |

Store a joint angle trajectory in the deque.

Performs forward kinematics to convert it to end effector poses in the torso frame.

**Parameters:**
:   |  |  |
    | --- | --- |
    | msg | A trajectory\_msgs.msg.JointTrajectory object. |

|  |  |  |  |
| --- | --- | --- | --- |
| def hrl\_haptic\_mpc.waypoint\_generator.WaypointGenerator.poseTrajectoryCallback | ( |  | *self*, |
|  |  |  | *msg* |
|  | ) |  |  |

Store a trajectory of poses in the deque.

Converts it to the 'torso\_frame' if required.

**Parameters:**
:   |  |  |
    | --- | --- |
    | msg | A geometry\_msgs.msg.PoseArray object |

|  |  |  |  |
| --- | --- | --- | --- |
| def hrl\_haptic\_mpc.waypoint\_generator.WaypointGenerator.robotStateCallback | ( |  | *self*, |
|  |  |  | *msg* |
|  | ) |  |  |

Store the current pose from the haptic state publisher.

**Parameters:**
:   |  |  |
    | --- | --- |
    | msg | RobotHapticState messge object |

|  |  |  |  |  |  |
| --- | --- | --- | --- | --- | --- |
| def hrl\_haptic\_mpc.waypoint\_generator.WaypointGenerator.start | ( |  | *self* | ) |  |

Start the waypoint generator publishing waypoints.

|  |  |  |  |
| --- | --- | --- | --- |
| def hrl\_haptic\_mpc.waypoint\_generator.WaypointGenerator.straightLineTrajectory | ( |  | *self*, |
|  |  |  | *current\_pose*, |
|  |  |  | *goal\_pose*, |
|  |  |  | *max\_pos\_step*, |
|  |  |  | *max\_ang\_step* |
|  | ) |  |  |

Returns the next waypoint along a straight line trajectory from the current gripper pose to the goal pose.

The step size towards the goal is configurable through the parameters passed in.

**Parameters:**
:   |  |  |
    | --- | --- |
    | current\_pose | geometry\_msgs.msg.Pose |
    | goal\_pose | geometry\_msgs.msg.Pose |
    | max\_pos\_step | = scalar float for position step size to take (metres) |
    | max\_ang\_step | = scalar float for orientation step size to take (radians) |

**Returns:**
:   A geometry\_msgs.msg.Pose to send to the MPC

---

The documentation for this class was generated from the following file:

- /home/jdh/ros\_sandbox/hrl\_haptic\_manipulation\_in\_clutter/hrl\_haptic\_mpc/src/hrl\_haptic\_mpc/waypoint\_generator.py


All Classes Namespaces Functions Variables

---

Generated on Thu Jan 17 2013 16:21:14 for HRL Haptic MPC by  

 1.7.6.1
