# Supplementary material for: In-home and remote use of robotic body surrogates by people with profound motor deficits
Source: PLoS One. 2019 Mar 15;14(3):e0212904. doi: 10.1371/journal.pone.0212904 (PMC6420017; doi:10.1371/journal.pone.0212904)
Supplement: S1 Source Code — This includes all custom code used in the described system and related evaluations. (ZIP) [file pone.0212904.s009.zip › robotic_body_surrogates_source_code/hrl-haptic-manip-pkgs.zip!/hrl-haptic-manip-pkgs/hrl_haptic_mpc/docs/html/functions_func.html]

HRL Haptic MPC: Class Members - Functions


HRL Haptic MPC

A haptic model predictive controller

- Main Page
- Packages
- Classes

- Class List
- Class Index
- Class Members

- All
- Functions
- Variables

- \_
- a
- c
- d
- e
- f
- g
- i
- j
- m
- o
- p
- r
- s
- t
- u
- z

### - \_ -

- \_\_init\_\_()
  : hrl\_haptic\_mpc.haptic\_mpc.HapticMPC
  , hrl\_haptic\_mpc.robot\_haptic\_state\_node.RobotHapticStateServer
  , hrl\_haptic\_mpc.waypoint\_generator.WaypointGenerator
  , hrl\_haptic\_mpc.skin\_client.TaxelArrayClient
- \_\_str\_\_()
  : hrl\_haptic\_mpc.haptic\_mpc.MPCData

### - a -

- addSkinTopic()
  : hrl\_haptic\_mpc.skin\_client.TaxelArrayClient
- addSkinTopicCallback()
  : hrl\_haptic\_mpc.skin\_client.TaxelArrayClient
- angularDistanceBetweenPoses()
  : hrl\_haptic\_mpc.waypoint\_generator.WaypointGenerator

### - c -

- closeGripperHandler()
  : hrl\_haptic\_mpc.mpc\_teleop\_rviz.MPCTeleopInteractiveMarkers
- contactForceTransformationMatrix()
  : hrl\_haptic\_mpc.haptic\_mpc.HapticMPC
- contactStiffnessMatrix()
  : hrl\_haptic\_mpc.haptic\_mpc.HapticMPC

### - d -

- deltaForceBounds()
  : hrl\_haptic\_mpc.haptic\_mpc.HapticMPC
- deltaQpJepGen()
  : hrl\_haptic\_mpc.haptic\_mpc.HapticMPC
- disablePps()
  : hrl\_haptic\_mpc.mpc\_teleop\_rviz.MPCTeleopInteractiveMarkers
- distanceBetweenPoses()
  : hrl\_haptic\_mpc.waypoint\_generator.WaypointGenerator

### - e -

- enableHapticMPC()
  : hrl\_haptic\_mpc.haptic\_mpc.HapticMPC
- enablePps()
  : hrl\_haptic\_mpc.mpc\_teleop\_rviz.MPCTeleopInteractiveMarkers

### - f -

- FK\_vanilla()
  : hrl\_haptic\_mpc.gen\_sim\_arms.RobotSimulatorKDL

### - g -

- generateWaypoint()
  : hrl\_haptic\_mpc.waypoint\_generator.WaypointGenerator
- get\_joint\_limits()
  : hrl\_haptic\_mpc.hrl\_arm.HRLArmKinematics
- getDesiredJointAngles()
  : hrl\_haptic\_mpc.haptic\_mpc.HapticMPC
- getJointAngles()
  : hrl\_haptic\_mpc.haptic\_mpc.HapticMPC
- getJointStiffness()
  : hrl\_haptic\_mpc.haptic\_mpc.HapticMPC
- getMessageHeader()
  : hrl\_haptic\_mpc.waypoint\_generator.WaypointGenerator
- getMPCData()
  : hrl\_haptic\_mpc.haptic\_mpc.HapticMPC
- getOrientationStep()
  : hrl\_haptic\_mpc.waypoint\_generator.WaypointGenerator
- getPositionStep()
  : hrl\_haptic\_mpc.waypoint\_generator.WaypointGenerator
- getSkinData()
  : hrl\_haptic\_mpc.haptic\_mpc.HapticMPC
  , hrl\_haptic\_mpc.skin\_client.TaxelArrayClient
- getTaxelLocationAndJointList()
  : hrl\_haptic\_mpc.pr2\_skin\_client.PR2\_SkinClient
  , hrl\_haptic\_mpc.sim\_skin\_client.SimSkinClient
  , hrl\_haptic\_mpc.skin\_client.TaxelArrayClient
- getTrimmedSkinData()
  : hrl\_haptic\_mpc.skin\_client.TaxelArrayClient
- getWaypointFromTrajectory()
  : hrl\_haptic\_mpc.waypoint\_generator.WaypointGenerator
- goalCallback()
  : hrl\_haptic\_mpc.haptic\_mpc.HapticMPC
- goalMotionForHand()
  : hrl\_haptic\_mpc.haptic\_mpc.HapticMPC
- goalOrientationInQuat()
  : hrl\_haptic\_mpc.haptic\_mpc.HapticMPC
- goalPoseCallback()
  : hrl\_haptic\_mpc.waypoint\_generator.WaypointGenerator
- goalPositionHandler()
  : hrl\_haptic\_mpc.mpc\_teleop\_rviz.MPCTeleopInteractiveMarkers
- goalPositionOrientationHandler()
  : hrl\_haptic\_mpc.mpc\_teleop\_rviz.MPCTeleopInteractiveMarkers

### - i -

- IK()
  : hrl\_haptic\_mpc.hrl\_arm.HRLArmKinematics
- initCody()
  : hrl\_haptic\_mpc.robot\_haptic\_state\_node.RobotHapticStateServer
  , hrl\_haptic\_mpc.waypoint\_generator.WaypointGenerator
- initComms()
  : hrl\_haptic\_mpc.haptic\_mpc.HapticMPC
  , hrl\_haptic\_mpc.mpc\_teleop\_rviz.MPCTeleopInteractiveMarkers
  , hrl\_haptic\_mpc.waypoint\_generator.WaypointGenerator
- initControlParametersFromServer()
  : hrl\_haptic\_mpc.haptic\_mpc.HapticMPC
- initMarkers()
  : hrl\_haptic\_mpc.mpc\_teleop\_rviz.MPCTeleopInteractiveMarkers
- initMenu()
  : hrl\_haptic\_mpc.mpc\_teleop\_rviz.MPCTeleopInteractiveMarkers
- initMPCData()
  : hrl\_haptic\_mpc.haptic\_mpc.HapticMPC
- initPR2()
  : hrl\_haptic\_mpc.robot\_haptic\_state\_node.RobotHapticStateServer
  , hrl\_haptic\_mpc.waypoint\_generator.WaypointGenerator
- initRobot()
  : hrl\_haptic\_mpc.robot\_haptic\_state\_node.RobotHapticStateServer
  , hrl\_haptic\_mpc.haptic\_mpc.HapticMPC
- initSim()
  : hrl\_haptic\_mpc.robot\_haptic\_state\_node.RobotHapticStateServer
- initSim3()
  : hrl\_haptic\_mpc.waypoint\_generator.WaypointGenerator
- interactiveMarkerLocationCallback()
  : hrl\_haptic\_mpc.mpc\_teleop\_rviz.MPCTeleopInteractiveMarkers

### - j -

- jacobian()
  : hrl\_haptic\_mpc.gen\_sim\_arms.RobotSimulatorKDL
  , hrl\_haptic\_mpc.hrl\_arm.HRLArmKinematics
- joint\_states\_cb()
  : hrl\_haptic\_mpc.urdf\_arm\_darpa\_m3.URDFArm
- jointTrajectoryCallback()
  : hrl\_haptic\_mpc.waypoint\_generator.WaypointGenerator

### - m -

- matrixListToMultiarray()
  : hrl\_haptic\_mpc.multiarray\_to\_matrix.MultiArrayConverter
- modifyPR2Taxels()
  : hrl\_haptic\_mpc.robot\_haptic\_state\_node.RobotHapticStateServer
- modifyRobotSpecificTaxels()
  : hrl\_haptic\_mpc.robot\_haptic\_state\_node.RobotHapticStateServer
- mpcMonitorCallback()
  : hrl\_haptic\_mpc.haptic\_mpc.HapticMPC
- multiArrayToMatrixList()
  : hrl\_haptic\_mpc.multiarray\_to\_matrix.MultiArrayConverter

### - o -

- openGripperHandler()
  : hrl\_haptic\_mpc.mpc\_teleop\_rviz.MPCTeleopInteractiveMarkers

### - p -

- poseTrajectoryCallback()
  : hrl\_haptic\_mpc.waypoint\_generator.WaypointGenerator
- publishDeltaControlValues()
  : hrl\_haptic\_mpc.haptic\_mpc.HapticMPC
- publishDesiredJointAngles()
  : hrl\_haptic\_mpc.haptic\_mpc.HapticMPC
- publishRobotState()
  : hrl\_haptic\_mpc.robot\_haptic\_state\_node.RobotHapticStateServer

### - r -

- removeSkinTopic()
  : hrl\_haptic\_mpc.skin\_client.TaxelArrayClient
- removeSkinTopicCallback()
  : hrl\_haptic\_mpc.skin\_client.TaxelArrayClient
- robotStateCallback()
  : hrl\_haptic\_mpc.haptic\_mpc.HapticMPC
  , hrl\_haptic\_mpc.waypoint\_generator.WaypointGenerator

### - s -

- set\_tooltip()
  : hrl\_haptic\_mpc.hrl\_arm.HRLArmKinematics
- setTrimThreshold()
  : hrl\_haptic\_mpc.skin\_client.TaxelArrayClient
- skinCallback()
  : hrl\_haptic\_mpc.skin\_client.TaxelArrayClient
- start()
  : hrl\_haptic\_mpc.waypoint\_generator.WaypointGenerator
  , hrl\_haptic\_mpc.haptic\_mpc.HapticMPC
  , hrl\_haptic\_mpc.mpc\_teleop\_rviz.MPCTeleopInteractiveMarkers
  , hrl\_haptic\_mpc.robot\_haptic\_state\_node.RobotHapticStateServer
- stopArmHandler()
  : hrl\_haptic\_mpc.mpc\_teleop\_rviz.MPCTeleopInteractiveMarkers
- straightLineTrajectory()
  : hrl\_haptic\_mpc.waypoint\_generator.WaypointGenerator

### - t -

- transformTaxelArray()
  : hrl\_haptic\_mpc.skin\_client.TaxelArrayClient
- trimSkinContacts()
  : hrl\_haptic\_mpc.skin\_client.TaxelArrayClient
- trimTaxelArray()
  : hrl\_haptic\_mpc.skin\_client.TaxelArrayClient

### - u -

- updateContactJacobians()
  : hrl\_haptic\_mpc.robot\_haptic\_state\_node.RobotHapticStateServer
- updateController()
  : hrl\_haptic\_mpc.haptic\_mpc.HapticMPC
- updateTorsoPose()
  : hrl\_haptic\_mpc.robot\_haptic\_state\_node.RobotHapticStateServer
- updateWeightsCallback()
  : hrl\_haptic\_mpc.haptic\_mpc.HapticMPC

### - z -

- zeroSkinHandler()
  : hrl\_haptic\_mpc.mpc\_teleop\_rviz.MPCTeleopInteractiveMarkers


All Classes Namespaces Functions Variables

---

Generated on Thu Jan 17 2013 16:21:14 for HRL Haptic MPC by  

 1.7.6.1
