# Supplementary material for: In-home and remote use of robotic body surrogates by people with profound motor deficits
Source: PLoS One. 2019 Mar 15;14(3):e0212904. doi: 10.1371/journal.pone.0212904 (PMC6420017; doi:10.1371/journal.pone.0212904)
Supplement: S1 Source Code — This includes all custom code used in the described system and related evaluations. (ZIP) [file pone.0212904.s009.zip › robotic_body_surrogates_source_code/hrl-haptic-manip-pkgs.zip!/hrl-haptic-manip-pkgs/hrl_haptic_mpc/docs/html/functions_vars.html]

HRL Haptic MPC: Class Members - Variables


HRL Haptic MPC

A haptic model predictive controller

- Main Page
- Packages
- Classes

- Class List
- Class Index
- Class Members

- All
- Functions
- Variables

- current\_topics\_pub
  : hrl\_haptic\_mpc.skin\_client.TaxelArrayClient
- data\_lock
  : hrl\_haptic\_mpc.skin\_client.TaxelArrayClient
- gain\_lock
  : hrl\_haptic\_mpc.haptic\_mpc.HapticMPC
- goal\_lock
  : hrl\_haptic\_mpc.haptic\_mpc.HapticMPC
- joint\_names
  : hrl\_haptic\_mpc.haptic\_mpc.HapticMPC
  , hrl\_haptic\_mpc.robot\_haptic\_state\_node.RobotHapticStateServer
- last\_msg\_time
  : hrl\_haptic\_mpc.haptic\_mpc.HapticMPC
- monitor\_lock
  : hrl\_haptic\_mpc.haptic\_mpc.HapticMPC
- mpc\_data
  : hrl\_haptic\_mpc.haptic\_mpc.HapticMPC
- mpc\_error
  : hrl\_haptic\_mpc.haptic\_mpc.HapticMPC
- msg
  : hrl\_haptic\_mpc.haptic\_mpc.HapticMPC
- skin\_data
  : hrl\_haptic\_mpc.skin\_client.TaxelArrayClient
- skin\_subs
  : hrl\_haptic\_mpc.skin\_client.TaxelArrayClient
- skin\_topic\_list
  : hrl\_haptic\_mpc.skin\_client.TaxelArrayClient
- tf\_lstnr
  : hrl\_haptic\_mpc.skin\_client.TaxelArrayClient
- topic\_lock
  : hrl\_haptic\_mpc.skin\_client.TaxelArrayClient
- torso\_frame
  : hrl\_haptic\_mpc.skin\_client.TaxelArrayClient
- trim\_threshold
  : hrl\_haptic\_mpc.skin\_client.TaxelArrayClient
- trimmed\_skin\_data
  : hrl\_haptic\_mpc.skin\_client.TaxelArrayClient


All Classes Namespaces Functions Variables

---

Generated on Thu Jan 17 2013 16:21:14 for HRL Haptic MPC by  

 1.7.6.1
