# Supplementary material for: In-home and remote use of robotic body surrogates by people with profound motor deficits
Source: PLoS One. 2019 Mar 15;14(3):e0212904. doi: 10.1371/journal.pone.0212904 (PMC6420017; doi:10.1371/journal.pone.0212904)
Supplement: S1 Source Code — This includes all custom code used in the described system and related evaluations. (ZIP) [file pone.0212904.s009.zip › robotic_body_surrogates_source_code/hrl-haptic-manip-pkgs.zip!/hrl-haptic-manip-pkgs/hrl_haptic_mpc/docs/html/namespacehrl__haptic__mpc.html]

HRL Haptic MPC: hrl\_haptic\_mpc Namespace Reference


HRL Haptic MPC

A haptic model predictive controller

- Main Page
- Packages
- Classes

- Packages

hrl\_haptic\_mpc Namespace Reference

---

## Detailed Description

**Author:**
:   C Kemp
:   A Jain
:   M Killpack

**Version:**
:   0.1

**Copyright:**
:   Simplified BSD Licence

This module contains the core mathematics used for the Haptic MPC.

**Author:**
:   Jeff Hawke jhawke@gatech.edu

**Version:**
:   0.1

**Copyright:**
:   Simplified BSD Licence


All Classes Namespaces Functions Variables

---

Generated on Thu Jan 17 2013 16:21:14 for HRL Haptic MPC by  

 1.7.6.1
