# Supplementary material for: In-home and remote use of robotic body surrogates by people with profound motor deficits
Source: PLoS One. 2019 Mar 15;14(3):e0212904. doi: 10.1371/journal.pone.0212904 (PMC6420017; doi:10.1371/journal.pone.0212904)
Supplement: S1 Source Code — This includes all custom code used in the described system and related evaluations. (ZIP) [file pone.0212904.s009.zip › robotic_body_surrogates_source_code/hrl-assistive-pkgs.zip!/hrl-assistive-pkgs/assistive_teleop/vci-www/tab_interface.html]

Robots for Humanity Web Page


Your browser does not support HTML5 canvas

|  |  |
| --- | --- |
| On Image Click: |  |

---


---

- Default Controls
- AR Servoing
- Traj. Playback
- Ellipsoidal Control
- TTS

|  |  |
| --- | --- |
| Control Head  Control Left Hand  Control Right Hand  Control Base | |
| **Movement Frame:** | Torso   Hand |

|  |  |  |  |
| --- | --- | --- | --- |
|  |  |  |  |
|  |  |  |  |
|  |  |  |  |

|  |  |  |  |
| --- | --- | --- | --- |
|  |  |  |  |
|  |  |  |  |
|  |  | | |  |

1. Activity

2. Arm

Right
Left

3. Trajectory

Play

Pause

Stop

Move to Setup

Execute Traj.

Setup AND Execute

|  |
| --- |
| Register Head |||  |
| --- |
| Shaving || Ellipsoid Controller |
| Adjust Mirror |
||  |
| --- |
| Start/Stop Razor |
| Move to Selection |
| Stop |

| Translate | | | |
| --- | --- | --- | --- |
|  |  |  |  |
|  |  |  |  |
|  |  |  |  |
|  |  |  |  |

| Rotate | | | |
| --- | --- | --- | --- |
|  |  |  |  |
|  |  |  |  |
|  |  |  |  |
|  | Reset | | |  |

Grippers | | Torso || Left | Right | U/D |
| Open | Open | Max |
|  |  |  |
| Close | Close | Min |
