# Supplementary material for: In-home and remote use of robotic body surrogates by people with profound motor deficits
Source: PLoS One. 2019 Mar 15;14(3):e0212904. doi: 10.1371/journal.pone.0212904 (PMC6420017; doi:10.1371/journal.pone.0212904)
Supplement: S1 Source Code — This includes all custom code used in the described system and related evaluations. (ZIP) [file pone.0212904.s009.zip › robotic_body_surrogates_source_code/hrl-assistive-pkgs.zip!/hrl-assistive-pkgs/assistive_teleop/vci-www/vci.html]

Video-centric Web Interface


### Main Menu

Look


Spine


Left Hand


Right Hand


Drive

### Controls

Zoom In

Zoom Out

Tuck Arms

Re-zero Bumper

#### Click on video to drive.

#### Step Size

XS

S

M

L

#### Position/Rotation

Hand Position

Wrist Rotation

3D Peek

Move Aside

Move to Setup

Re-zero Skin

#### Position/Rotation

Hand Position

Wrist Rotation

3D Peek

Move Aside

Move to Setup

Re-zero Skin

Toggle Shaver

### Task Menu

Select a Planned Task

Approach
Move Object
Get Tool
Select arm

Left Arm
Right Arm
Select a Tool

Scratcher
Towel
Toothbrush
Shaver
Lotion Applicator

Start Task

Undo

?

RUN-STOP

Turn Left

Turn Right


Release on contact

Release on contact
