# Supplementary material for: In-home and remote use of robotic body surrogates by people with profound motor deficits
Source: PLoS One. 2019 Mar 15;14(3):e0212904. doi: 10.1371/journal.pone.0212904 (PMC6420017; doi:10.1371/journal.pone.0212904)
Supplement: S1 Source Code — This includes all custom code used in the described system and related evaluations. (ZIP) [file pone.0212904.s009.zip › robotic_body_surrogates_source_code/hrl-assistive-pkgs.zip!/hrl-assistive-pkgs/assistive_teleop/www/calib_tab_interface.html]

**Adjust a feeding position** \| \| In-Out \| Left-Right \| Up-Down; 2cm 3cm 4cm 5cm 6cm 7cm 8cm 9cm \| -3cm -2cm -1cm 0cm 1cm 2cm 3cm \| -5cm -4cm -3cm -2cm -1cm 0cm 1cm |  | **Select a task** \|; Feeding \| **Reset parameters** \|; Reset Position \|; Reset System \| |
