# Supplementary material for: In-home and remote use of robotic body surrogates by people with profound motor deficits
Source: PLoS One. 2019 Mar 15;14(3):e0212904. doi: 10.1371/journal.pone.0212904 (PMC6420017; doi:10.1371/journal.pone.0212904)
Supplement: S1 Source Code — This includes all custom code used in the described system and related evaluations. (ZIP) [file pone.0212904.s009.zip › robotic_body_surrogates_source_code/hrl-assistive-pkgs.zip!/hrl-assistive-pkgs/assistive_teleop/www/hz_tab_interface.html]

Robots for Humanity Web Page


Please provide feedback

|  |  |  |  |  |  |  |
| --- | --- | --- | --- | --- | --- | --- |
| Did the robot scoop yogurt? | | | | | yes | no |
| Did any unexpected collisions occur? | | | | | yes | no |
| Was there unexpected noise during scooping?   (excluding noise from the robot) | | | | | yes | no |

|  |  |  |  |  |  |  |
| --- | --- | --- | --- | --- | --- | --- |
| Did the robot feed you? | | | | | yes | no |
| Did the robot generate a beep (anomaly detection)? | | | | | yes | no |
| If the robot generated a beep, was there any unexpected collision, noise, uncomfortable contact, etc? | | | | | yes | no |

|  |  |
| --- | --- |
| SEND | SKIP |

Press anywhere to stop

|  |  |  |
| --- | --- | --- |
|  |  |  |

|  |  |  |  |  |  |
| --- | --- | --- | --- | --- | --- |
|  |  |  |  |  |  |
| ---     --- | | | | | |
|  | | | | | |

- Default Controls
- FeedingTask
- Stabbing Task
- Calibration

|  |  |
| --- | --- |
| Control Head  Control Left Hand  Control Right Hand  Control Base | |
| **Movement Frame:** | Auto Torso Hand |

|  |  |  |  |
| --- | --- | --- | --- |
|  |  |  |  |
|  |  |  |  |
|  |  |  |  |

|  |  |  |  |
| --- | --- | --- | --- |
|  |  |  |  |
|  |  |  |  |
|  |  |  |  |

**Select a Task**

Scooping / Stabbing

Clean Spoon

Feeding

|  |  |
| --- | --- |
| Detected Anomaly: | Classifying |
| No anomaly detected | |

Advanced Options

Feeding Anomaly Detection Sensitivity

|  |  |  |
| --- | --- | --- |
| Down |  | Up |

|  |  |
| --- | --- |
| Accuracy with   most recent 10 data | :  0.0% |

|  |  |  |  |  |  |
| --- | --- | --- | --- | --- | --- |
| **Left Gripper** | Left Close |  | | | Left Open |
| **Right Gripper** | Right Close |  | | | Right Open |
| **Torso** | Down |  | | | Up |
