# Supplementary material for: In-home and remote use of robotic body surrogates by people with profound motor deficits
Source: PLoS One. 2019 Mar 15;14(3):e0212904. doi: 10.1371/journal.pone.0212904 (PMC6420017; doi:10.1371/journal.pone.0212904)
Supplement: S1 Source Code — This includes all custom code used in the described system and related evaluations. (ZIP) [file pone.0212904.s009.zip › robotic_body_surrogates_source_code/hrl-assistive-pkgs.zip!/hrl-assistive-pkgs/assistive_teleop/www/js/keyboard/calculator.html]

Virtual Keyboard Basic Scientific Calculator Demo


Main Demo
Basic
Mobile
Layouts
Navigate
Calculator
Playground
Documentation
Download
Issues

# Virtual Keyboard Scientific Calculator

#### This is a very basic demo of a scientific calculator.\*
