# Supplementary material for: In-home and remote use of robotic body surrogates by people with profound motor deficits
Source: PLoS One. 2019 Mar 15;14(3):e0212904. doi: 10.1371/journal.pone.0212904 (PMC6420017; doi:10.1371/journal.pone.0212904)
Supplement: S1 Source Code — This includes all custom code used in the described system and related evaluations. (ZIP) [file pone.0212904.s009.zip › robotic_body_surrogates_source_code/hrl-assistive-pkgs.zip!/hrl-assistive-pkgs/assistive_teleop/www/js/keyboard/index.html]

Virtual Keyboard


Main Demo
Basic
Mobile
Layouts
Navigate
Calculator
Playground
Documentation
Download
Issues

# Virtual Keyboard

#### Original by Jeremy Satterfield, updated & maintained by Rob Garrison

- ...
- ...
- ...
- ...

### Click on the keyboard title, then scroll down to see its code

## QWERTY Text

  

\* Placeholder (watermark).  
\* Autocomplete.

```
HTML
=======
<input class="qwerty" type="text" placeholder="Enter something...">

Script
=======
// Autocomplete demo
var availableTags = ["ActionScript", "AppleScript", "Asp", "BASIC", "C", "C++", "Clojure",
 "COBOL", "ColdFusion", "Erlang", "Fortran", "Groovy", "Haskell", "Java", "JavaScript",
 "Lisp", "Perl", "PHP", "Python", "Ruby", "Scala", "Scheme" ];

$('.qwerty:eq(0)')
 .keyboard({ layout: 'qwerty' })
 .autocomplete({
  source: availableTags
 })
 .addAutocomplete()
 .addTyping();
```

## QWERTY Password

```
HTML
=======
<input class="qwerty" type="password">

Script
=======
$('.qwerty:eq(1)')
 .keyboard({ 
  openOn : null,
  stayOpen : true,
  layout : 'qwerty'
 })
 .addTyping();
$('#passwd').click(function(){
 $('.qwerty:eq(1)').getkeyboard().reveal();
});
```

  

\* Click icon to open keyboard.  
\* Stay open until accept/cancel.  
\* Key hover disabled in this type.

## QWERTY Text Area

  

\* Locked input (no manual input).  
\* Position the hidden caret!  
\* Known readonly bug in Safari.

```
HTML
=======
<textarea class="qwerty"></textarea>

Script
=======
$('.qwerty:eq(2)')
 .keyboard({
  layout   : 'qwerty',
  lockInput: true // prevent manual keyboard entry
 })
 .addTyping();
```

## International

```
HTML
=======
<textarea id="inter"></textarea>

Script
=======
$('#inter')
 .keyboard({ layout: 'international' })
 .addTyping();

Script - typing extension
=======
$('#inter').getkeyboard().reveal().typeIn('\tHello \b\n\tWorld', 500);
```

## Alphabetical

```
HTML
=======
<textarea id="alpha"></textarea>

Script
=======
$('#alpha')
 .keyboard({ layout: 'alpha' })
 .addTyping();
```

## Dvorak

```
HTML
=======
<textarea id="dvorak"></textarea>

Script
=======
$('#dvorak')
 .keyboard({ layout: 'dvorak' })
 .addTyping();
```

## Num Pad

```
HTML
=======
<input id="num" class="alignRight" type="text">

Script
=======
$('#num')
 .keyboard({
  layout : 'num',
  restrictInput : true, // Prevent keys not in the displayed keyboard from being typed in
  preventPaste : true,  // prevent ctrl-v and right click
  autoAccept : true
 })
 .addTyping();
```

  

\* Input restricted.  
\* Pasting (ctrl-v) not allowed.  
\* Auto accept content.

## Custom: Hex

```
HTML
=======
<input id="hex" type="text">

Script
=======
$('#hex')
 .keyboard({
  layout: 'custom',
  customLayout: {
   'default' : [
    'C D E F',
    '8 9 A B',
    '4 5 6 7',
    '0 1 2 3',
    '{bksp} {a} {c}'
   ]
  },
  maxLength : 6,
  restrictInput : true, // Prevent keys not in the displayed keyboard from being typed in
  useCombos : false // don't want A+E to become a ligature
 })
 .addTyping();
```

  

\* maxLength = 6.  
\* Input restricted.

## Custom: Meta Sets

```
HTML
=======
<textarea id="meta"></textarea>

Script
=======
$('#meta')
 .keyboard({
  layout: 'custom',
  display: {
   'alt'    : 'AltGr:It\'s all Greek to me',
   'meta1'  : '\u2666:end of alphabet', // Diamond with label that shows in the title (spaces are okay here)
   'meta2'  : '\u2665:Russian',         // Heart
   'meta3'  : '\u2663:zodiac',          // Club
   'meta99' : '\u2660:numbers'          // Spade
  },
  customLayout: {
   'default' : [
    // Add labels using a ":" after the key's name and replace spaces with "_"
    // without the labels this line is just 'a b c d e f g'
    'a:a_letter,_that_sounds_like_"ey" b:a_bug_that_makes_honey c:is_when_I_look_around d:a_grade,_I_never_got e:is_what_girls_say_when_they_run_away_from_me f:u,_is_what_I_say_to_those_screaming_girls! g:gee,_is_that_the_end_of_my_wittiness?',
    '{shift} {alt} {meta1} {meta2} {meta3} {meta99}',
    '{bksp} {sp:1} {accept} {cancel}'
   ],
   'shift' : [
    'A B C D E F G',
    '{shift} {alt} {meta1} {meta2} {meta3} {meta99}',
    '{bksp} {sp:1} {accept} {cancel}'
   ],
   'alt' : [ 
    '\u03b1 \u03b2 \u03b3 \u03b4 \u03b5 \u03b6 \u03b7', // lower case Greek
    '{shift} {alt} {meta1} {meta2} {meta3} {meta99}',
    '{bksp} {sp:1} {accept} {cancel}'
   ],
   'alt-shift' : [
    '\u0391 \u0392 \u0393 \u0394 \u0395 \u0396 \u0397', // upper case Greek
    '{shift} {alt} {meta1} {meta2} {meta3} {meta99}',
    '{bksp} {sp:1} {accept} {cancel}'
   ],
   'meta1' : [
    't u v w x y z', // lower case end of alphabet
    '{shift} {alt} {meta1} {meta2} {meta3} {meta99}',
    '{bksp} {sp:1} {accept} {cancel}'
   ],
   'meta1-shift' : [
    'T U V W X Y Z', // upper case
    '{shift} {alt} {meta1} {meta2} {meta3} {meta99}',
    '{bksp} {sp:1} {accept} {cancel}'
   ],
   'meta1-alt' : [
    '0 9 8 7 6 5 4', // numbers
    '{shift} {alt} {meta1} {meta2} {meta3} {meta99}',
    '{bksp} {sp:1} {accept} {cancel}'
   ],
   'meta1-alt-shift' : [
    ') ( * & ^ % $', // shifted numbers
    '{shift} {alt} {meta1} {meta2} {meta3} {meta99}',
    '{bksp} {sp:1} {accept} {cancel}'
   ],
   'meta2' : [
    '\u0430 \u0431 \u0432 \u0433 \u0434 \u0435 \u0436', // lower case Russian
    '{shift} {alt} {meta1} {meta2} {meta3} {meta99}',
    '{bksp} {sp:1} {accept} {cancel}'
   ],
   'meta2-shift' : [
    '\u0410 \u0411 \u0412 \u0413 \u0414 \u0415 \u0416', // upper case Russian
    '{shift} {alt} {meta1} {meta2} {meta3} {meta99}',
    '{bksp} {sp:1} {accept} {cancel}'
   ],
   'meta3' : [
    '\u2648 \u2649 \u264A \u264B \u264C \u264D \u264E', // Zodiac
    '{shift} {alt} {meta1} {meta2} {meta3} {meta99}',
    '{bksp} {sp:1} {accept} {cancel}'
   ],
   'meta99' : [
    '1 2 3 4 5 6 7', // only because I ran out of ideas
    '{shift} {alt} {meta1} {meta2} {meta3} {meta99}',
    '{bksp} {sp:1} {accept} {cancel}'
   ]
  }
 })
 .addTyping();

Script - typing extension
=======
var meta = $('#meta').getkeyboard();
meta.reveal().typeIn('aBcD1112389\u2648\u2649', 700, function(){ meta.accept(); alert('all done!'); });
```

## Custom: Junk

```
HTML
=======
<textarea id="junk"></textarea>

Script
=======
$('#junk')
 .keyboard({
  layout: 'custom',
  customLayout: {
   'default' : [
    'a e i o u y c',
    '` \' " ~ {sp:1} {dec}',
    '{tab} {enter} {bksp}',
    '{space}',
    '{accept} {cancel} {shift}'
   ],
   'shift' : [
    'A E I O U Y C',
    '` \' " ~ {sp:1} {dec}',
    '{t} {sp:1} {e} {sp:1} {b}',
    '{space}',
    '{a} {sp:1} {c} {sp:1} {s}'
   ]
  },
  // Added here as an example on how to add combos
  combos : {
   'a' : { e: '\u00e6' },
   'A' : { E: '\u00c6' }
  },
  // example callback function
  accepted : function(e, keyboard, el){ alert('The content "' + el.value + '" was accepted!'); }
 })
 .addTyping();
```

  

\* Use shift key - see action key sizes.  
\* Use the decimal - only one allowed.  
\* Type in 'ae' and other combo keys.  
\* Accept content to see the callback.

## Custom: Mapped Keys

```
HTML
=======
<textarea id="map"></textarea>

Script
=======
// *** Mapped keys ***
$('#map').keyboard({
 layout: 'custom',
 customLayout: {
  'default' : [ 
   // "n(a):title_or_tooltip"; n = new key, (a) = actual key, ":label" = title_or_tooltip (use an underscore "_" in place of a space " ")
   '\u03b1(a):lower_case_alpha_(type_a) \u03b2(b):lower_case_beta_(type_b) \u03b3(c):lower_case_gamma_(type_c) \u03b4(d):lower_case_delta_(type_d) \u03b5(e):lower_case_epsilon_(type_e) \u03b6(f):lower_case_zeta_(type_f) \u03b7(g):lower_case_eta_(type_g)', // lower case Greek
   '{shift} {accept} {cancel}'
  ],
  'shift' : [
   '\u0391(A) \u0392(B) \u0393(C) \u0394(D) \u0395(E) \u0396(F) \u0397(G)', // upper case Greek
   '{shift} {accept} {cancel}'
  ]
 },
 usePreview: false // no preveiw
});
```

  

\* No preview window.  
\* Type in a-g.  
\* Type in A-G (shifted).

## Custom: Hidden

Hidden input


```
HTML
=======
<a href="#" class="hiddenInput">Hidden input</a>
<!-- DON'T use type="hidden" because IE doesn't like hidden inputs -->
<input id="hidden" type="text" style="display:none;">

Script
=======
// *** Hidden input example ***
// click on a link - add focus to hidden input
$('.hiddenInput').click(function(){
 $('#hidden').trigger('focus.keyboard');
 return false;
});
// Initialize keyboard script on hidden input
// set "position.of" to the same link as above
$('#hidden')
 .keyboard({ 
  layout   : 'qwerty',
  position : {
   of : $('.hiddenInput'),
   my : 'center top',
   at : 'center top'
  }
 })
 .addTyping();
```
