# Supplementary material for: In-home and remote use of robotic body surrogates by people with profound motor deficits
Source: PLoS One. 2019 Mar 15;14(3):e0212904. doi: 10.1371/journal.pone.0212904 (PMC6420017; doi:10.1371/journal.pone.0212904)
Supplement: S1 Source Code — This includes all custom code used in the described system and related evaluations. (ZIP) [file pone.0212904.s009.zip › robotic_body_surrogates_source_code/hrl-assistive-pkgs.zip!/hrl-assistive-pkgs/assistive_teleop/www/js/keyboard/layouts.html]

Virtual Keyboard Language Layouts


Main Demo
Basic
Mobile
Layouts
Navigate
Calculator
Playground
Documentation
Download
Issues

# Virtual Keyboard Language Layouts

#### This is a work in progress.

  

Albanian (qwerty)
Albanian (qwertz)
Arabic (azerty)
Arabic (qwerty-1)
Arabic (qwerty-2)
Arabic (qwerty-3)
Arabic (qwerty-4)
Belarusian (Belarus)
Bengali (qwerty-1)
Bengali (qwerty-2)
Bulgarian (qwerty)
Catalan (qwerty)
Danish (qwerty)
French (azerty-1)
French (azerty-2)
German (qwertz-1)
German (qwertz-2)
Magyar (qwertz-1) utf8
Magyar (qwertz-2) utf8
Magyar (qwertz-1) ansi
Magyar (qwertz-2) ansi
Russian (qwerty)
Swedish (qwerty)
Swedish (dvorak)
Turkish (q)
Turkish (f)
