# Supplementary material for: In-home and remote use of robotic body surrogates by people with profound motor deficits
Source: PLoS One. 2019 Mar 15;14(3):e0212904. doi: 10.1371/journal.pone.0212904 (PMC6420017; doi:10.1371/journal.pone.0212904)
Supplement: S1 Source Code — This includes all custom code used in the described system and related evaluations. (ZIP) [file pone.0212904.s009.zip › robotic_body_surrogates_source_code/hrl-assistive-pkgs.zip!/hrl-assistive-pkgs/assistive_teleop/www/js/keyboard/mobile.html]

Virtual Keyboard Mobile Demo


Main Demo
Basic
Mobile
Layouts
Navigate
Calculator
Playground
Documentation
Download
Issues

# Virtual Keyboard jQuery Mobile Themes

#### Until the jQurey Mobile Theme Roller is complete, use the color selections below to change the keyboard


Container  
(background)
A
B
C
D
E

Regular Buttons  
(non-action)
A
B
C
D
E

Hovered Buttons  
(all)
A
B
C
D
E

Action Buttons  
(Enter)
A
B
C
D
E

Active Buttons  
(Shift down)
A
B
C
D
E

\* Note \* The "Active Buttons" setting is limited in that if the swatch letter of the Regular buttons is greater than the swatch letter of the Active Buttons, the theme doesn't apply properly. For example, Regular button swatch B with Active Button swatch A won't work because of the way the css is set up.
